# Network Structure within the Cerebellar Input Layer Enables Lossless Sparse Encoding

**DOI:** 10.1016/j.neuron.2014.07.020

**Published:** 2014-08-20

**Authors:** Guy Billings, Eugenio Piasini, Andrea Lőrincz, Zoltan Nusser, R. Angus Silver

**Affiliations:** 1 Department of Neuroscience, Physiology and Pharmacology, University College London, London, WC1E 6BT UK; 2 Institute of Experimental Medicine, Hungarian Academy of Sciences, H-1083 Budapest, Hungary

## Abstract

The synaptic connectivity within neuronal networks is thought to determine the information processing they perform, yet network structure-function relationships remain poorly understood. By combining quantitative anatomy of the cerebellar input layer and information theoretic analysis of network models, we investigated how synaptic connectivity affects information transmission and processing. Simplified binary models revealed that the synaptic connectivity within feedforward networks determines the trade-off between information transmission and sparse encoding. Networks with few synaptic connections per neuron and network-activity-dependent threshold were optimal for lossless sparse encoding over the widest range of input activities. Biologically detailed spiking network models with experimentally constrained synaptic conductances and inhibition confirmed our analytical predictions. Our results establish that the synaptic connectivity within the cerebellar input layer enables efficient lossless sparse encoding. Moreover, they provide a functional explanation for why granule cells have approximately four dendrites, a feature that has been evolutionarily conserved since the appearance of fish.

## Highlights

• Network connectivity sets trade-off between informative and sparse encoding
• Feedforward networks with few connections are optimal for lossless, sparse encoding
• Best-performing networks match synaptic connectivity in the cerebellar input layer
• Explanation for why cerebellar granule cells have approximately 4 dendrites

## Teaser

Synaptic connectivity is thought to determine information processing in neuronal networks, yet network structure-function relationships are poorly understood. Billings et al. show that synaptic connectivity within feedforward networks determines the trade-off between information transmission and sparse encoding.

## Introduction

Different regions of the brain exhibit distinct anatomical structures, cell morphologies, and synaptic connectivities and perform specific computational tasks. However, linking the structure to function (Honey et al., 2007) or dysfunction (Dyhrfjeld-Johnsen et al., 2007) has proved difficult, because the synaptic connectivity, neuronal properties, and the computations performed are usually poorly defined. Some notable exceptions exist in circuits where the function is clear. In the retina, asymmetric spatial patterns of synaptic input onto starburst amacrine cells contribute to direction selectivity (Briggman et al., 2011). In mouse primary visual cortex, neurons with similar orientation selectivity have been shown to be preferentially connected (Ko et al., 2011). In pattern generator circuits within the spinal cord, distinct neuronal subtypes compute different gaits during locomotion (Talpalar et al., 2013). Despite these advances, the contribution that synaptic connectivity makes to information processing remains unclear in most brain regions.

The cerebellar cortex is particularly well suited to network structure-function analysis due to its relatively simple three layer structure, few neuronal cell types, and its well-established role in motor control (Eccles et al., 1967). Moreover, there is wide consensus that the cerebellar input layer, or granule cell layer (GCL), transforms mossy fiber (MF) inputs, conveying sensory and efferent copy information, into a higher dimensional, sparser code (Marr, 1969). This increases the separation between the patterns (Olshausen and Field, 2004), thereby enabling downstream cerebellar circuits to perform more effective associative learning (Albus, 1971, D’Angelo and De Zeeuw, 2009, Marr, 1969, Medina and Mauk, 2000, Schweighofer et al., 2001, Tyrrell and Willshaw, 1992), adaptive filtering (Fujita, 1982), and binary addressing (Kanerva, 1988). Three basic properties are required for divergent feedforward networks to perform effective pattern separation: (1) information is conserved, (2) the dimensionality of the output coding is larger than that of the input, and (3) the output code is sparse. However, the contribution that synaptic connectivity makes to these functions remains poorly understood.

To investigate how the network structure of the cerebellar input layer affects its function, we first quantified specific anatomical properties of the network. We then developed a simplified model of the GCL that was analytically tractable, allowing us to quantify information transmission and sparse encoding in networks with different synaptic connectivities. Finally, we tested predictions from our analytical approach on the relationship between network structure and function using biologically detailed network models of spiking neurons, whose parameters were constrained by experimental measurements. Our results show that the synaptic connectivity within the cerebellar input layer, where GCs receive an average of approximately four excitatory MF inputs, is well suited for performing sparse encoding without loss of information.

## Results

### Quantification of the Cerebellar Input Layer Structure and Development of a 3D Model of Excitatory Network Connectivity

**Figure 1:**
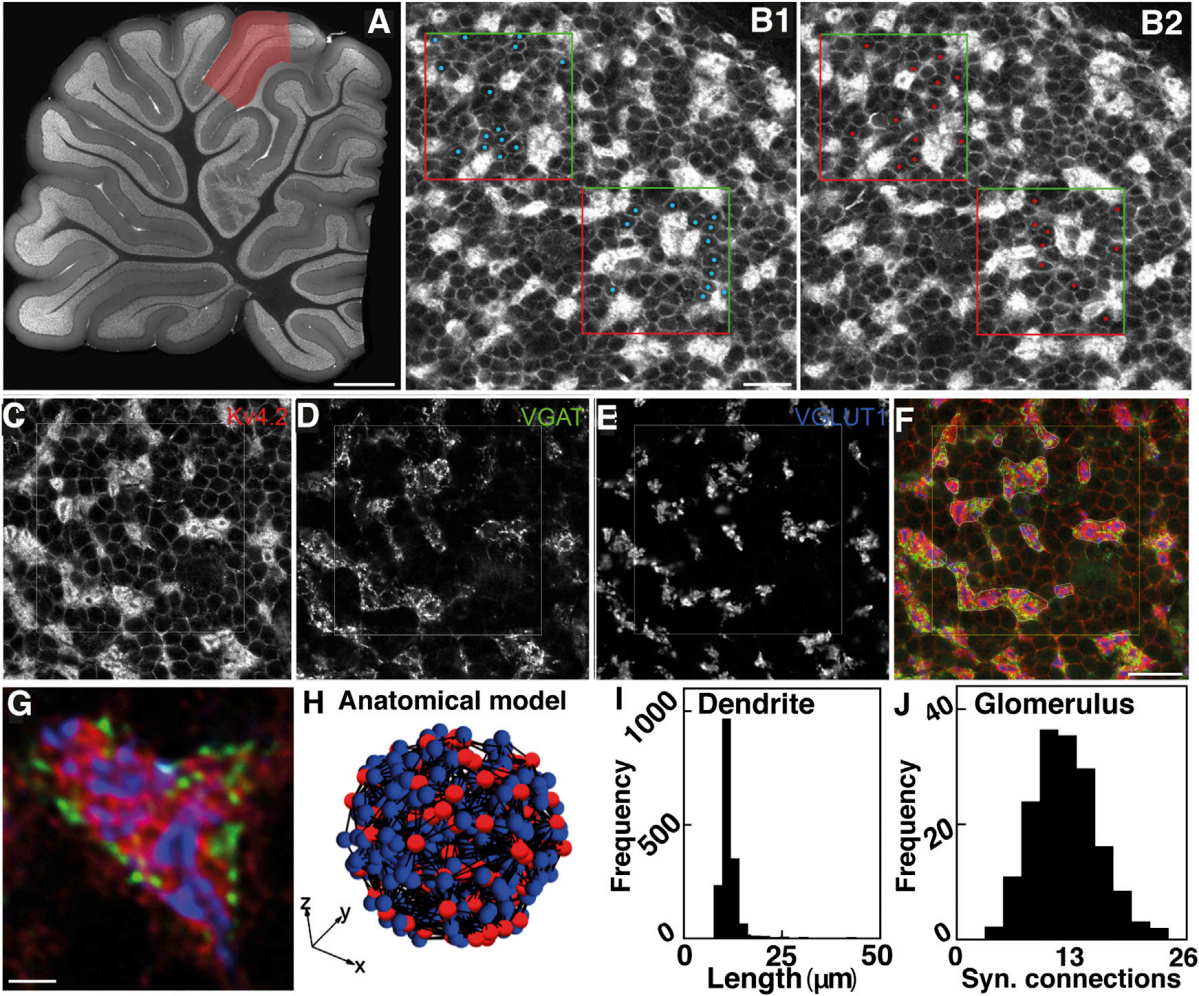
Granule Cell and Glomerular Density in the Rat Cerebellum and Construction of a Local Granule Cell Layer Model (A) Measurements performed in lobule VIa (red area) of a parasagittal slice of cerebellar vermis. (B) Regions of granule cell layer (GCL) with pairs of sections (left and right) and frames used for the unbiased counting method (cells on green edges are counted and on red edges excluded). (C–E) Area of GCL immunolabeled for Kv4.2 (C) showing circular GC somatic outlines, VGAT (D), and VGLUT1 (E). (F) Overlay of immunolabels for Kv4.2 (red), VGAT (green), and VGLUT1 (blue). (G) Colabeling of the three markers used to demarcate a glomerulus. (H) 3D anatomically constrained model of the local GCL network, consisting of a 40-μm-radius ball of glomeruli (red) and GCs (blue) with four dendrites per GC (black lines). (I) Distribution of GC dendrite length in the local GCL network model. (J) Distribution of the number of GC dendrites per MF rosette. (C)–(F) are at the same magnification, with scale bar on (F) applying to all panels. Scales, 1 mm in (A), 20 μm in (B)–(F), and 2 μm in G.

Cerebellar MFs form large en passant presynaptic structures called rosettes that form the core of each synaptic glomerulus, which also consists of Golgi cell axons, GC and Golgi cell dendrites, and a glial coat. While quantitative anatomical data are available on several cellular components across species (Harvey and Napper, 1988), the rosette-to-GC expansion ratio remains uncertain. To address this, we combined high-resolution confocal microscopy, multicolor immunofluorescence labeling, and an unbiased counting method to study the properties of the cerebellar GC layer in rat (Figures 1A and 1B). Immunolabeling for Kv4.2 delineated somatic plasma membranes and the dendrites of GCs (Figure 1C). GCs had a mean diameter of 6.72 ± 0.13 μm (n = 24) and mean density of 1.9 ± 0.14 × 10^6^ mm^−3^, similar to that previously reported (Harvey and Napper, 1988). Golgi cell axons and MF rosettes were identified with VGAT and VGlut1 immunolabeling, respectively (Figures 1D and 1E). Colabeling for all three molecules was used to identify glomeruli (Figures 1F and 1G), which occupied 28.8% ± 2.3% of the input layer volume and occurred at a density of 6.6 ± 1.5 × 10^5^ mm^−3^ (Figure S1 available online; Tables S1 and S2). The local glomeruli-to-GC and thus rosette-to-GC ratio is therefore 1:2.9.

We examined the likely spatial extent of a local GC layer network by building a 3D anatomical model of MF-GC connectivity (Figure 1H), using our measured parameters together with existing measurements of MF rosette spacing (20 μm parasagittal and 60 μm mediolateral; Sultan, 2001). The claw-like ending of each GC dendrite contacts a single MF rosette. GC dendrites rarely exceed 30 μm (e.g., 4% in the cat; Palay and Chan-Palay, 1974, Palkovits et al., 1972), making the likelihood of a GC being innervated by two or more rosettes from the same MF low (Livet et al., 2007). The number of GC dendrites is therefore equal to the number of MF synaptic connections per GC (*d*). Moreover, the MFs that converge onto a GC typically arise from multiple precerebellar nuclei (Huang et al., 2013). To recreate these conditions, model GCs were placed at random in a sphere at the measured anatomical density within a central subfield of MFs to minimize edge effects (Figure 1H). Each model GC made synaptic connections to randomly selected MF rosettes, with the constraint that the dendritic length should be close to 15 μm. In practice, they rarely exceeded 20 μm (Figure 1I), as observed experimentally. Since the maximum distance between two GCs that could share the same MF input was ∼40 μm, the largest ball of tissue that could be expected to have independent inputs was 80 μm in diameter, which is comparable to the thickness of the GC layer in rodents. Our anatomically constrained model of this “local GCL network” contained 176 MF synaptic rosettes and 509 GCs (Figure 1H). For an average of four dendrites per GC (Eccles et al., 1967), a single MF rosette made synaptic connections with an average of 12 different GCs (Figure 1J), consistent with estimates of 15–20 in monkey and cat (Eccles et al., 1967).

### Uniform Binary Network Model for Computing Information Transmission

**Figure 2:**
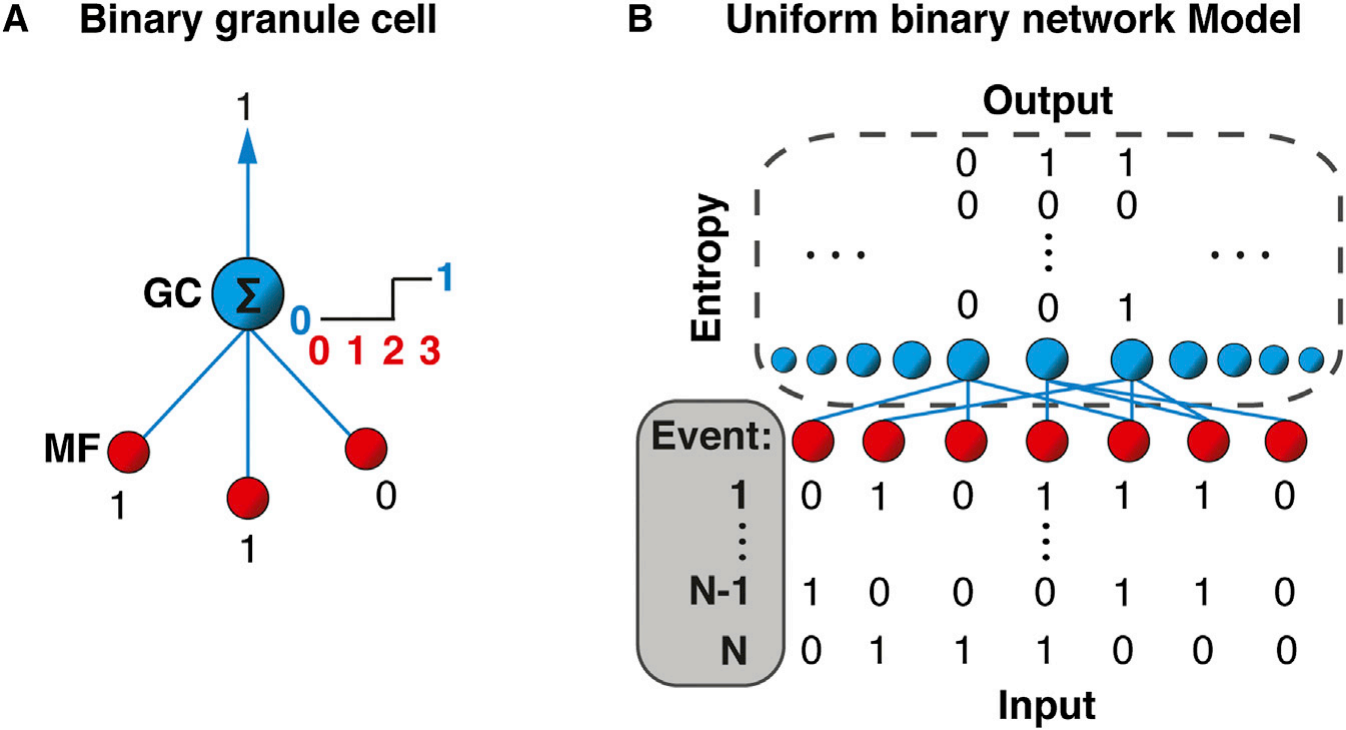
Schematic Representation of the Uniform Binary Network Model (A) Schematic diagram showing binary mossy fiber (MF) synaptic inputs (red) and a linear thresholding binary granule cell (GC, blue) with a single MF synaptic connection per dendrite (blue lines). GC output is 1 if the sum of its MF input values is equal to the threshold or greater and 0 otherwise. (B) Uniform binary network model is a random bipartite graph consisting of binary MFs and linear threshold GC units. Network with 3 MF synaptic connections per GC (*d* = 3), shown only for the three central GCs for clarity. N events, encoded as binary MF input patterns, are transformed into a binary GC output patterns. GC population entropy is calculated from the distribution of output patterns.

Direct calculation of information transmission for all possible configurations of MF input drive and network structure is computationally intractable. We therefore simplified our local GCL network model to permit mathematical analysis by removing the spatial dependences in the synaptic connectivity but conserving the random nature of the connectivity, the MF rosette-to-GC expansion ratio, and the number of MF synaptic connections per GC (Supplemental Information; Figure S2). In addition, we reduced rate-coded signals in MFs and GCs to binary representations, where 0 and 1 represent quiescence and activity, respectively. When a spatial pattern of binary MF activity is presented, each GC sums its equally weighted inputs and compares the sum to its threshold value. The GC output is 1 if the sum exceeds the threshold and 0 otherwise (Figure 2A). We refer to these binary MF and GC networks as the “uniform binary network model” or UBN model. Figure 2B shows a schematic illustration of such a model with three synaptic connections per GC. Sensory-motor “events” are represented as random binary MF activity patterns. Each one is thresholded by the GCs and transformed into a binary GC output pattern, from which the information encoded by the GC population can be calculated.

Although the simplifying assumptions required for calculating information in this manner are substantial, the cerebellar GCL is particularly amenable to this approach. Since the dimensionality of sensory-motor sample space is vast, we considered raw input as being nonrepeating from event-to-event, with each sensory-motor event being directly mapped to an MF input pattern. Real MF activity patterns encoding sensory-motor events consist of two main stochastic components: the subset of MFs in the network that were activated and the trial-to-trial variability in the spiking, synaptic transmission, and membrane noise present in an individual connection. In the UBN model, we consider the simplified case where the variance is dominated by the MF patterns themselves and the stochasticity of transmission is negligible, because fluctuations are averaged over multiple release sites and are integrated by GCs. Indeed, GCs have intrinsic and synaptic properties that make them well-suited to a binary representation because: (1) their soma and dendrites form a single electrical compartment, thereby acting as a point neuron (Silver et al., 1992), (2) much of their excitatory drive is composed of slow spillover-mediated AMPAR and NMDAR conductances that build up over time during rate-coded MF input (Arenz et al., 2008, DiGregorio et al., 2002, Schwartz et al., 2012), (3) GCs have only two to seven excitatory MF inputs, and (4) multiple MF inputs are typically required to reach spike threshold (Jörntell and Ekerot, 2006, Schwartz et al., 2012). Thus, GC activity reflects a thresholded version of a few active MF inputs, making the simplification to a thresholded binary representation (Figure 2A) more reasonable than for cells with larger numbers of inputs.

To quantify information transmission across UBN models, we developed an analytical method for rapidly calculating Shannon Information (Shannon, 1948), which corresponded to the entropy in the GC population under noise-free conditions (Figure 2B; Supplemental Information: Appendix, Equation 29). This allowed us to explore how information transmission depends on the synaptic connectivity of randomly connected networks, GC threshold, and the fraction of MFs active. In the limited cases where direct calculation of entropy was possible, it gave similar results to our analytical method (Figure S3). Although our analytical method can be used to calculate information transmission for the full set of MF input patterns (i.e., 2^176^ ≈10^53^), we restricted the number of patterns to the maximum number of events a local GCL network could encode in the lifetime of a small rodent in the wild. This is approximately one billion, if we assume that events are integrated in ∼30 ms windows (Figure S4B; Schwartz et al., 2012, van Beugen et al., 2013) for a lifetime of 1 year.

### Effect of Synaptic Connectivity and Neuronal Threshold on the Transmission and Transformation of Activity Patterns in Uniform Binary Network Models

**Figure 3:**
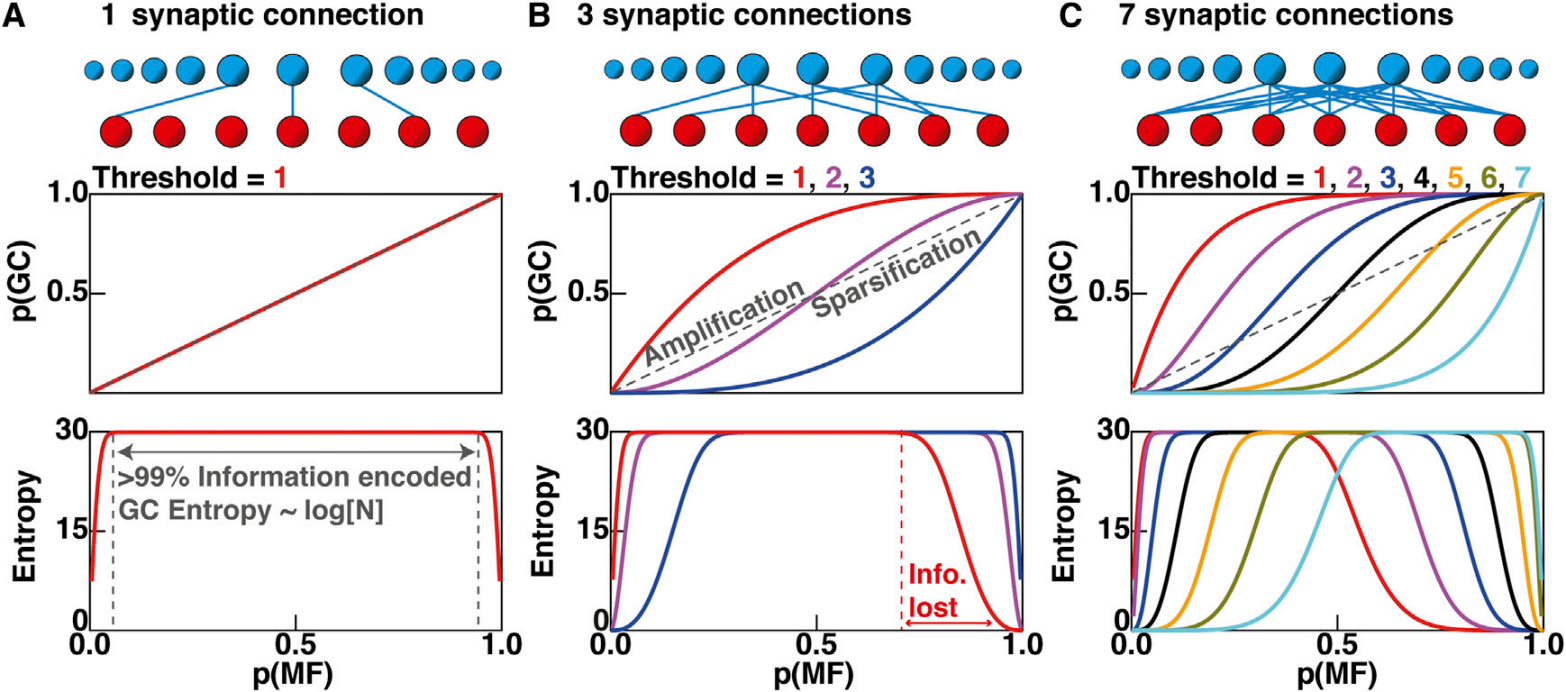
Number of Synaptic Connections per Neuron and Threshold Determine the Transmission and Transformation of Information in a Uniform Binary Network Model (A) Top: schematic illustration of mossy fibers (MF, red) and granule cells (GCs) (blue) for a binary network with one MF synaptic connection per GC (*d* = 1; blue lines, shown for 3 GCs only). Middle: GC activation probability (*p*(*GC*)) as a function of MF activation probability (*p*(*MF*)), red line for a threshold of 1. Gray dashed line indicates *p(GC)* = *p(MF)*. Bottom: information (entropy) in GC population as a function of *p*(*MF*) for one billion events. Vertical dashed lines indicate range of *p*(*MF*) where >99% of the information is encoded by the GC population. (B) Same as for (A) but for a network with *d* = 3 and all possible threshold values (1-3 red-blue). (C) Same as for (B) but for *d* = 7 (1-7 red-cyan).

We first analyzed the functional properties of the simplest configuration of the UBN model, with one synaptic connection per GC and a threshold of 1. Since GCs are 3-fold more numerous than MF synaptic rosettes, the absolute number of activated GC was higher than the number of MF inputs. Despite this expansion, the fraction of active MFs (or MF activation probability [*p(MF)*]) was equal to the fraction of active GCs (or GC activation probability [*p(GC)*]) (Figure 3A, middle), as expected for a simple relay network. The relationship between GC entropy and *p(MF)* had a truncated flat top because the information contained in the GC output patterns (Figure 3A) was limited by the maximum one billion unique MF input patterns and thus had a maximal value of 29.9 bits (i.e., log_2_(10^9^)). We defined such flat, saturated sections of the entropy versus *p*(*MF*) curve (Figure 3A, bottom) as full information transmission or lossless encoding regions (i.e., >99% of the event information). These calculations show that randomly connected feedforward networks with unitary synaptic connectivity can transmit all MF event information to GCs over a wide range of input activity. This is possible because the network operates far below the maximum transmission capacity of either its input or output (179 bits and 509 bits, respectively). However, this network does not perform sparse encoding.

For networks with more than one MF input per GC, the number of inputs required to reach threshold was a key variable, since it determined the fraction of GCs activated. Networks with three synaptic connections per GC had three possible threshold settings and thus three mappings from MF to GC activity (Figure 3B). For a threshold of 1, the fraction of active neuronal elements used to represent information was larger for the GC output than the MF input (*p*(*GC*) > *p*(*MF*)) over the whole MF input activity range (red line in Figure 3B, middle). Increasing the threshold to 2 produced GC activity that approximately matched the MF input activity, while increasing the threshold to 3 reduced the GC activity below that of the MFs across the whole range of input activity. Such sparsening of MF activity (i.e., *p*(*GC*) < *p*(*MF*)) is a key proposed function of the GCL (Albus, 1971, Marr, 1969).

At low threshold, full transmission of event information was achieved at low and intermediate MF input activities, but information was lost at high MF activities (Figure 3B, bottom, red). In contrast, at the highest threshold the lossless encoding range was shifted to higher MF activities (Figure 3B, bottom, blue). An intermediate threshold of 2 produced lossless encoding over nearly the whole MF activity range but did not perform effective sparsification. Setting the number of MF inputs per GC to seven increased the number of possible mappings between MFs and GCs (Figure 3C). Moreover, at high thresholds, sparsification became highly pronounced (i.e., *p(GC)* < < *p(MF)*), but the range of MF activation over which lossless encoding occurred was markedly reduced (Figure 3C, bottom). These results show that both synaptic connectivity and thresholding within the networks have a big impact on both the transmission and transformation of information. The inability of some networks to transmit information in particular *p*(*MF*) regions is likely to be highly disadvantageous, because in vivo recordings show that MFs exhibit a wide range of activity levels (Arenz et al., 2008, Rancz et al., 2007, van Kan et al., 1993). On the other hand, full information transmission without sparsification is also problematic, because the GCL circuit then fails to perform its main function.

### Trade-Off between Information Transmission and Sparsification in Uniform Binary Network Models with Fixed GC Threshold

**Figure 4:**
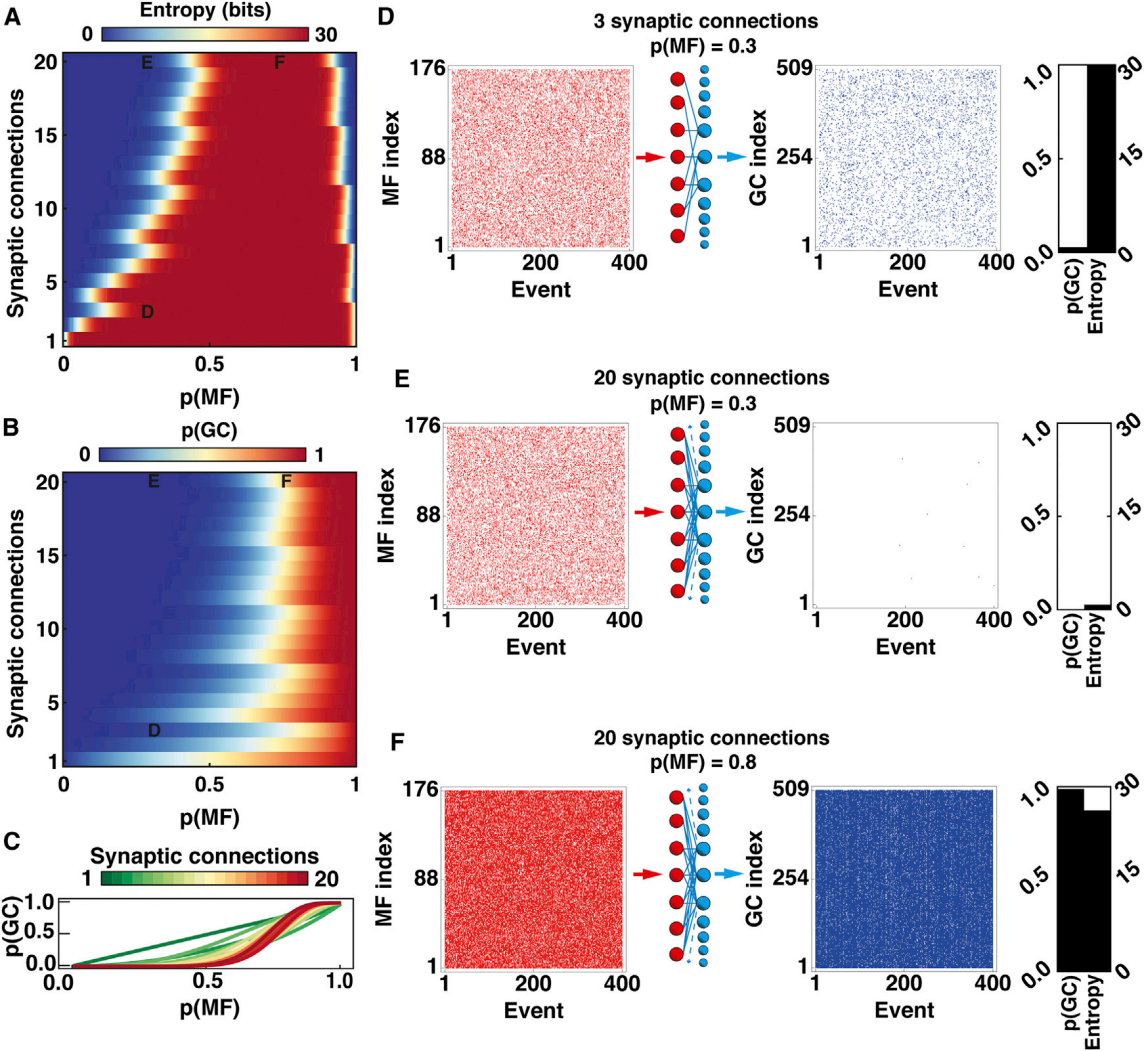
Effect of Synaptic Connectivity on Information Transmission and Population Activity in Uniform Binary Network Models with a Fixed Relative Threshold (A) Quantity of event information (entropy) encoded by the granule cell (GC) population across the full range of mossy fiber (MF) activation probability *p*(*MF*) for uniform binary network models with different numbers of synaptic connections per GC (*d*) and a fixed relative threshold φ = *ceiling [0.75* × *d]*. (B) GC activation probability *p(GC)* for the same network configurations as in (A). (C) Same as for (B) but visualized as a line plot to show the relationship between *p(GC)* and *p(MF)* for different models. (D) From left to right: a sample of 400 MF input patterns (events) with *p(MF)* = 0.3, where active MFs are red and inactive MFs are white, schematic network representation and GC output activity patterns (blue raster plot) for a network with *d* = 3 (see label D in panels A and B). Bar graph indicates *p(GC)* and entropy for one billion patterns, as for (A). (E) Same as for (D) but for *d* = 20 (see label E in panels A and B). (F) Same as for (D) but for *d* = 20 and *p(MF)* = 0.8 (see label F in panels A and B).

To investigate further how network connectivity affects information transmission and sparsification, we examined the case of a relatively high fixed threshold, since GCs typically require activation of three of their four MF inputs to fire (Jörntell and Ekerot, 2006, Schwartz et al., 2012) and spike threshold is dominated by the presence of a large tonic inhibitory conductance (Brickley et al., 1996, Duguid et al., 2012). To do this, we set the threshold to 75% of the number of synaptic connections per neuron or as close to it as discretisation allowed (using a ceiling function). For such fixed threshold networks, the lossless encoding range decreased with increasing numbers of inputs (Figure 4A).

In contrast to information transmission, GC sparseness tended to increase with the number of synaptic connections per GC, particularly at low-to-intermediate levels of MF activation (Figures 4B and 4C). Figures 4D and 4E illustrate how two networks with different numbers of synaptic connections per GC transform MF input at *p*(*MF*) = 0.3. While the GC activity of the network with 20 synaptic connections was substantially lower than that with 3, the highly connected network was unable to transmit information at low-to-intermediate values of *p(MF)*. When *p*(*MF*) was increased to 0.8, the high connectivity network became effective at transmitting information but did not encode the MF input more sparsely (Figure 4F). These results clearly demonstrate a trade-off between transmission and sparsening of input representations in simple feedforward networks and that synaptic connectivity and threshold determine the balance between these two competing functions.

To gain further insight into how the transmission-sparsification trade-off arises, we examined the relationship between the mean activity of the GC population and the mean activity of the MF population for different network connectivities (Figure 4C). For a single MF connection per GC and a threshold of 1, the network transfer function was linear, but as the number of connections increased the relationship became increasingly nonlinear. This lowered *p(GC)* across a wide range of *p(MF)*, resulting in greater sparsification. However, at these low activation levels, the GC population could not encode all the MF patterns and thus information was lost.

### Extension of Uniform Binary Network Models to Include Network-Activity-Dependent Thresholds

**Figure 5:**
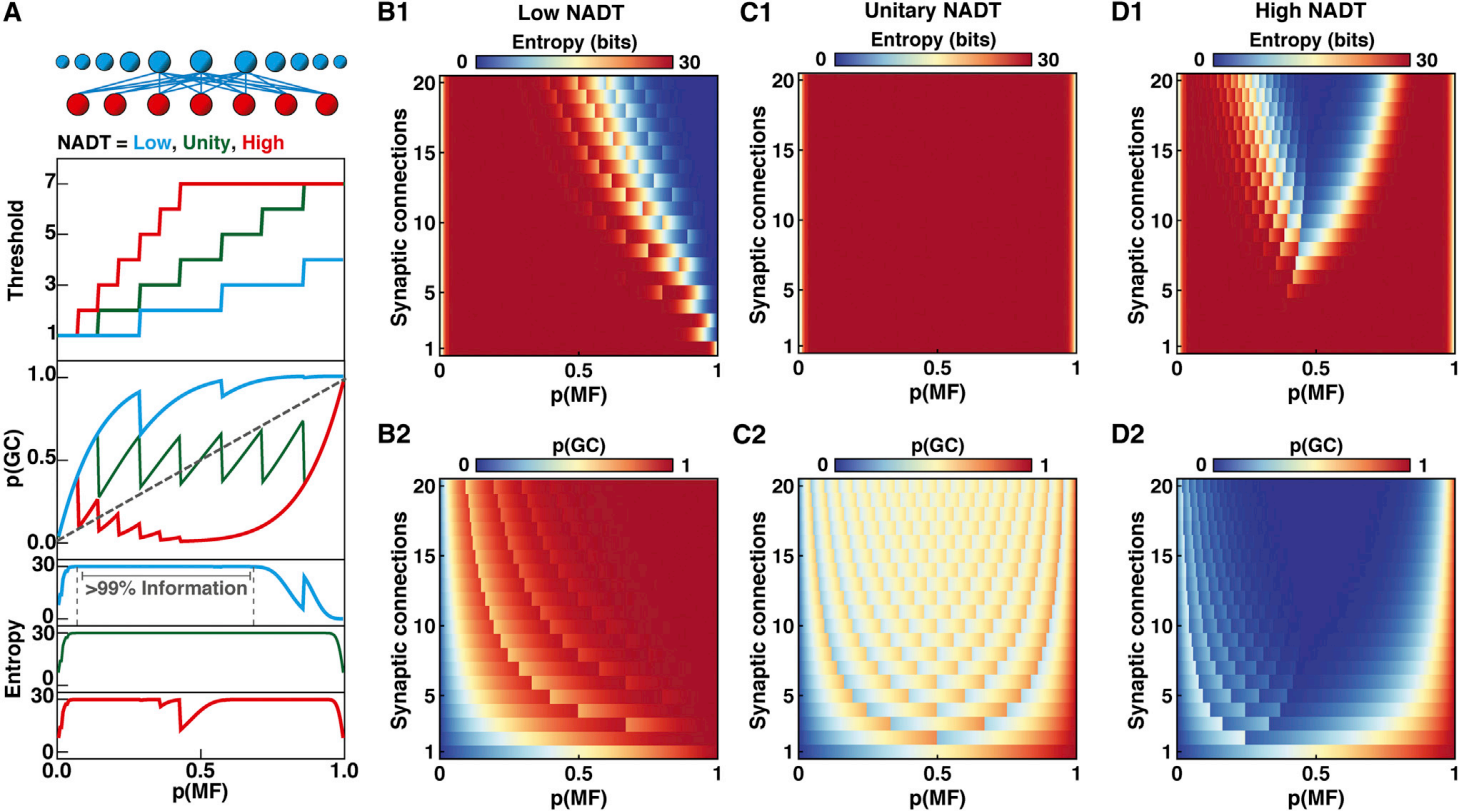
Effect of Activity-Dependent Threshold Regulation on the Trade-Off between Information Transmission and Sparsification (A) Top: uniform binary network model schematic with mossy fibers (MFs) in red and granule cells (GCs) in blue (top); GC network-activity-dependent threshold (NADT) for low (0.5, blue), unity (1.0, green), and high (2.0, red) NADT, for a network with seven synaptic connections per GC (*d* = 7; connections for center 3 GCs shown for clarity). Middle: GC activation probability (*p*(*GC*)) versus MF activation probability (*p*(*MF*)) for the NADT functions above. Bottom: information encoded by GCs for each threshold function. (B1 and B2) Information encoded by GCs and *p(GC)*, respectively, for low NADT networks with different *d*. (C1 and C2 and D1 and D2) Same as for (B1 and B2) for unity NADT and high NADT, respectively.

Golgi cells provide network-activity-dependent inhibition of GCs, via feedforward (Kanichay and Silver, 2008) and feedback inhibition (Cesana et al., 2013), although the impact of this phasic and spillover-mediated component is much weaker than tonic inhibition (Duguid et al., 2012). To mimic this activity-dependent change in inhibition in the UBN model, we implemented a network-activity-dependent threshold (NADT), which scaled the GC threshold in proportion to *p*(*MF*) (Experimental Procedures). For a network with seven synaptic inputs per GC (Figure 5A, top), a low NADT and an initial threshold of 1 resulted in an amplification (i.e., *p*(*GC*) > *p*(*MF*)) and only permitted lossless encoding over the lower portion of *p*(*MF*) (Figure 5A, cyan). Similar behavior was observed across all network connectivities, except those with few connections, which transmitted information across a wider range of *p*(*MF*) (Figure 5B1). However, none of the networks with low NADT performed sparsification, when averaged across *p(MF)* (Figure 5B2).

An NADT value of 1 enabled lossless transmission over nearly the entire MF input range for all networks tested, irrespective of the number of synaptic connections per GC (Figure 5C1). This was achieved by maintaining the GC activity around 0.5 (Figure 5C2), thereby maximizing encoding capacity. However, this strategy compromised sparsification. Increasing the NADT to high levels sparsened the GC representation (Figure 5D2) but introduced a lossy region for intermediate levels of MF activity for networks with more than 5 MF inputs per GC (Figure 5D1). These results show that unity NADT can enhance information transmission through feedforward networks with large numbers of synaptic connections per GC, but higher levels of NADT are required to sparsen input activity. However, only networks with small numbers of connections can perform lossless sparse encoding with high NADT, due to the network connectivity-dependent trade-off between information transmission and sparsification.

### Identification of Optimal Network Connectivities for Robust Lossless Sparse Encoding

**Figure 6:**
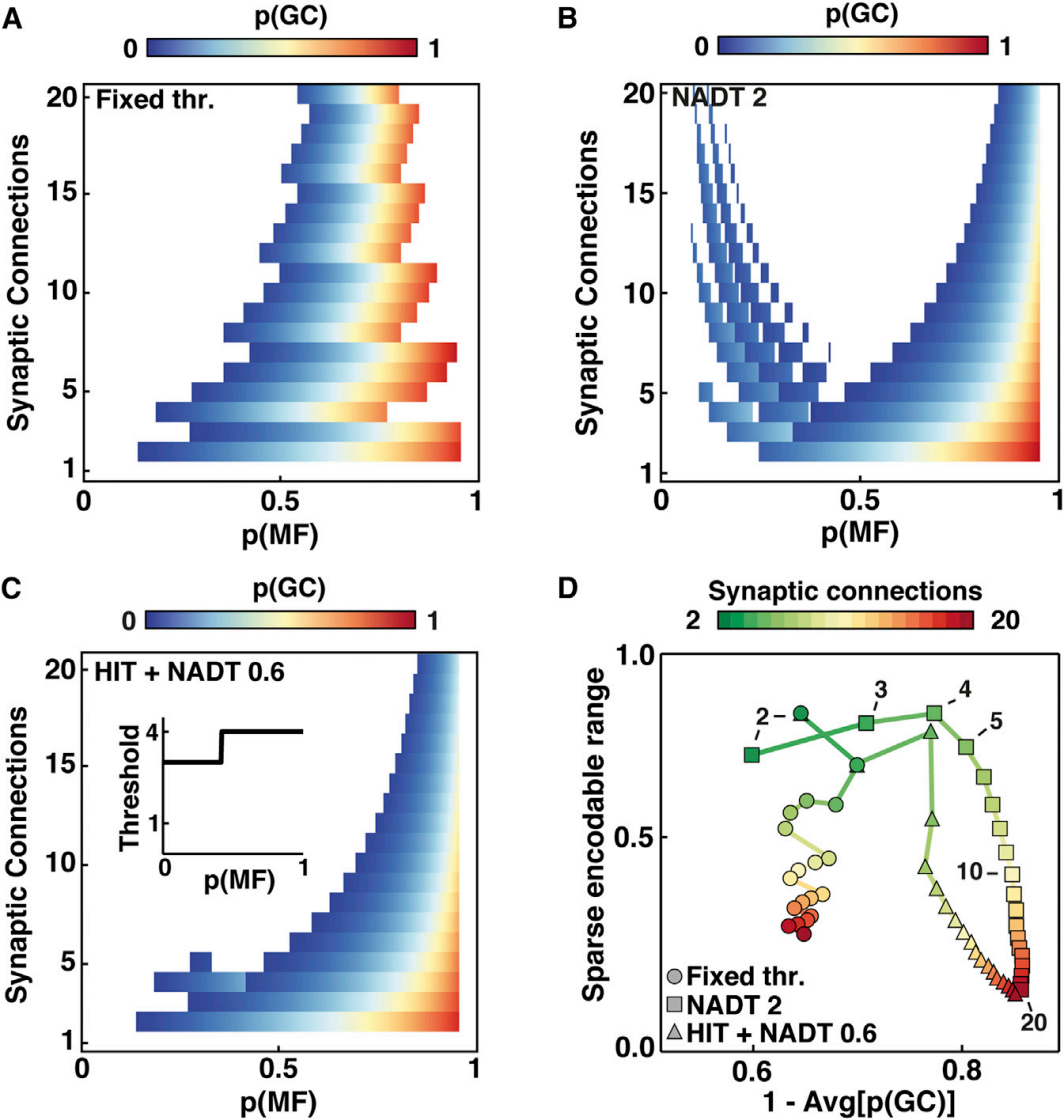
Networks that Best Perform Lossless Sparse Encoding Have Few Synaptic Connections per Neuron (A) Granule cell activation probability (*p*(*GC*)) versus mossy fiber activation probability (*p*(*MF*)) with different numbers of synaptic connections per GC (*d*) for a fixed relative threshold (φ = *ceiling[0.75* × *d]*). Colored regions indicate sparse encodable range, where >99% of information was encoded and *p(GC)* < *p(MF)*. (B and C) Same as for (A) but for network-activity-dependent threshold (NADT) = 2 and a high initial threshold (HIT) combined with NADT = 0.6, respectively. Inset in (C) shows threshold function for networks with *d* = 4. (D) Relationship between size of sparse encodable range and output sparseness (1-Avg[*p(GC)*] averaged across all values of *p(MF)*). Color code indicates *d* and circle, square, and triangle symbols show different threshold functions in (A), (B), and (C), respectively.

Since both information transmission and sparsification are essential for cerebellar operation, we investigated which feedforward network connectivity and threshold settings provided the best trade-off between these two competing functions. To do this, we analyzed our data set to find all network configurations that transmitted information without loss over the widest range of *p(MF)* and transformed MF patterns into a sparser GC representation. Figure 6A shows the range of *p(MF)* over which networks with different numbers of connections can transmit information losslessly and sparsify it for the fixed relative threshold case (75% of inputs). A comparable pattern was observed for networks with high NADT, but in these cases sparsening of the encoding was more effective at high *p(MF)* (Figure 6B). Combining a high initial threshold and NADT provided an effective strategy for sparse lossless encoding in networks with few synaptic connections (Figure 6C). To identify the best-performing networks, we found those networks where sparsification could not be improved without detriment to the range of *p(MF)* over which full information transmission was achieved and, conversely, the lossless *p(MF)* range could not be improved without detriment to sparsification. For the fixed threshold case, networks with two to three MF inputs per GCs performed sparse encoding over the widest range of *p(MF)* without loss of information (Figure 6D). As the number of synaptic connections per GC increased, the sparse encodable range declined due to information loss, with little improvement in average sparsification. Networks with NADT = 2, or with a high initial threshold combined with NADT, that best performed lossless sparse encoding over the largest *p(MF)* range also had few synaptic connections, with four being particularly effective (Figure 6D). As the number of dendrites increased further, the sparse encodable range fell steeply indicating that encoding became lossy.

Our analysis of UBN models predict that few synaptic connections per GC provide the best trade-off between information transmission and sparse encoding over a wide range of MF input activity. However, the simplifications required to develop our analytical treatment raise the question of whether these predictions are valid for biological networks, where sensory-motor signals are encoded by MF firing rate and synaptic conductances, and GC spike threshold is set by a tonic inhibitory conductance.

### A Biologically Detailed Network Model of the Cerebellar Input Layer

**Figure 7:**
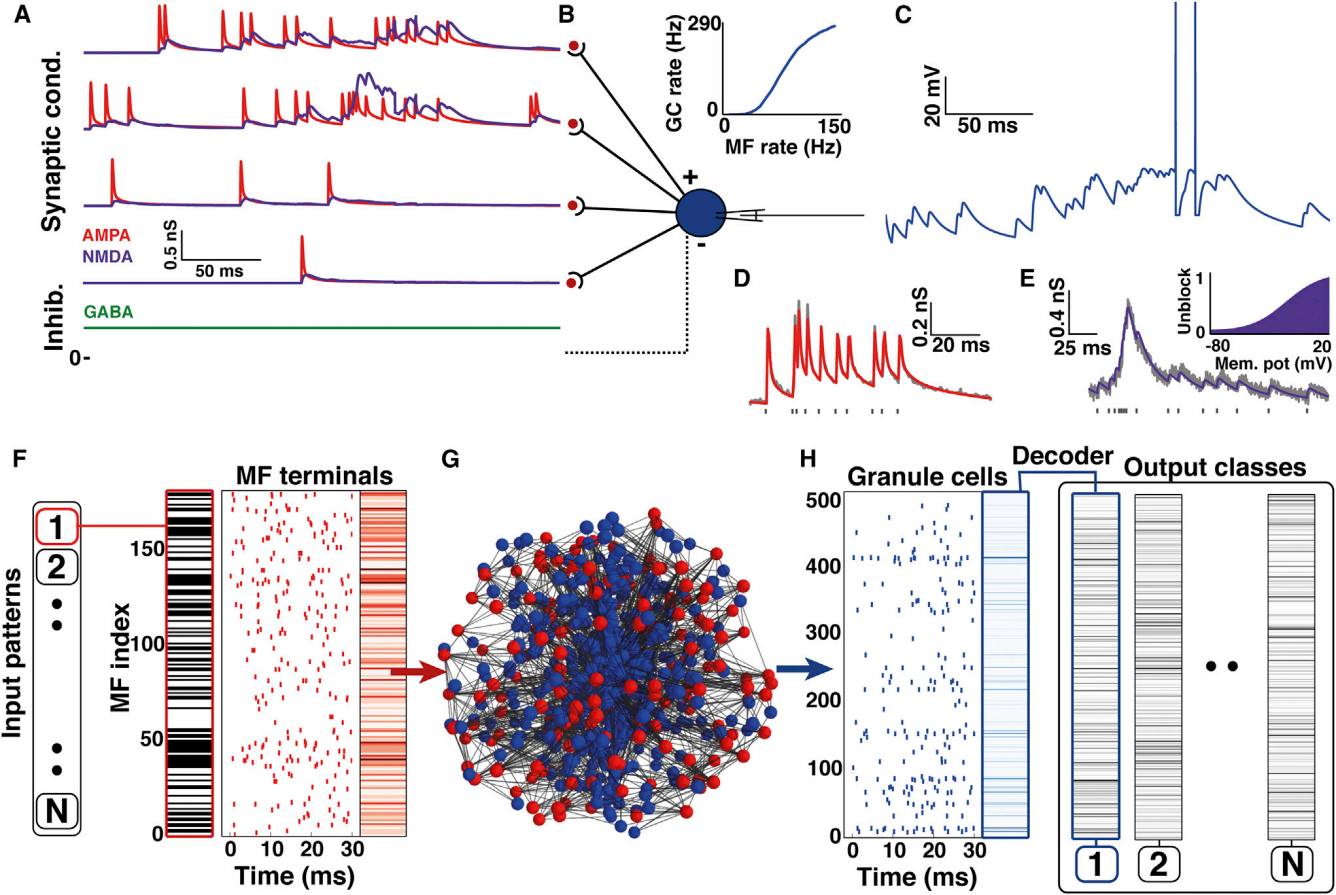
Construction and Analysis of an Experimentally Constrained Spiking Model of the Local Granule Cell Layer Network Incorporating Synaptic Mechanisms and Tonic Inhibition (A) Excitatory AMPAR (red) and NMDAR (purple) synaptic conductances for four independent mossy fiber (MF) inputs injected into a model granule cell (GC). Top two traces: active MFs with excitatory conductance driven by independent Poisson spike trains firing at 80 Hz. Lower two traces: inactive MF firing at 10 Hz. Bottom trace: tonic inhibitory GABA_A_R conductance (green). (B) Model GC with action potential firing rate-coded input-output relationship (above) for four synaptic inputs. (C) Membrane potential of model GC during synaptic input in (A). (D) Fit of the short-term plasticity model (red) of the AMPAR component to an experimental recording of a 100 Hz synaptic conductance train (gray). (E) Same as for (D) but for the NMDAR component and an 80 Hz conductance train. Inset: voltage dependence of NMDAR conductance. (F) A binary stimulus pattern was randomly selected from a set of N patterns (black active and white inactive on barcode). A Poisson spike train was generated for each MF input (80 Hz active, 10 Hz inactive; red raster plot), thereby setting the timing of synaptic conductances (as in A). Red barcode indicates spike counts for the given realization of the spike trains. (G) 3D view of the anatomically constrained local GCL network model with 176 MFs in red and 509 GCs in blue. (H) Raster plot of GC firing activity in response to the input. Blue barcode indicates GC spike count vector (measured over a 30 ms window), which was assigned to one of N output classes (black bar codes) defined using the k-means algorithm on a separate data set.

To test the validity of the predictions from our simple analytical model, we constructed a biologically detailed network model where each parameter was constrained by experimental measurements. We used the anatomically constrained local GC layer network model (Figure 1H), which captured the measured densities of MF synaptic rosettes and GCs and the spatial dependence of synaptic connectivity imposed by the finite length of GC dendrites. For each network configuration, we used a fixed instantiation of the randomly generated connectivity. GCs in the model were conductance based integrate-and-fire neurons with a capacitance (3.22 pF), input resistance (0.94 GΩ), and resting potential (−79.9 mV) set to the mean value obtained from GCs recorded at physiological temperature (Rothman et al., 2009, Schwartz et al., 2012). Information was represented in each MF input by the rate of an independent Poisson spike train (Arenz et al., 2008, van Kan et al., 1993) and each spike triggered excitatory synaptic responses in connected GCs. GCs received a fixed number of MF synaptic inputs (depending on the network configuration) in the form of trains of AMPAR- and NMDAR-mediated conductances (Figures 7A and 7B), producing trains of EPSPs and spikes (Figure 7C).

Since the GC input-output (I-O) relationship is determined predominantly by the properties of the MF synaptic conductances, which include slow glutamate spillover-mediated components (DiGregorio et al., 2002) and short-term plasticity (STP) (Saviane and Silver, 2006), we converted existing experimental measurements of Poisson trains of synaptic currents to conductances (Rothman et al., 2009, Schwartz et al., 2012) and used them to constrain STP models of synaptic AMPAR and NMDAR components (Figures 7D and 7E, respectively). Moreover, we used an NMDAR model that captured the measured voltage dependence of NMDARs in GCs (Figure 7E, inset; Schwartz et al., 2012). Lastly, inhibition was implemented with a tonic GABA_A_R-mediated inhibition, with a conductance of 438 pS and a reversal potential of −79.1 mV (Rothman et al., 2009, Seja et al., 2012; Figure 7A, green line). With these experimentally constrained settings, the I-O relationship of the model GC (Figure 7B) was similar to that observed in real GCs (Rothman et al., 2009, Schwartz et al., 2012). For networks with different numbers of synaptic inputs per GC the AMPAR- and NMDAR-mediated conductance amplitudes were scaled to conserve the total excitatory conductance (Supplemental Information). Biologically detailed networks were implemented using neuroConstruct (Gleeson et al., 2007) and simulated with NEURON (Carnevale and Hines, 2006).

### Quantification of Transmission and Transformation of Information in Biologically Detailed Spiking Network Models

We quantified the I-O relationships of biologically detailed networks using sets of MF input patterns (Figures 7F, 7G, and 7H). Each input pattern was generated by randomly selecting a subset of the 176 MF inputs and designating them to be active (black lines in barcode, Figure 7F), while the remainder were inactive. During the simulation, active MFs fired random Poisson trains with a mean rate of 80 Hz and inactive MFs fired at 10 Hz (red raster plot in Figure 7F), reflecting the properties of real MF rate-coded inputs (Arenz et al., 2008, van Kan et al., 1993). This resulted in individual GCs receiving both high- and low-frequency trains of synaptic input conductances (e.g., top two and bottom two traces in Figure 7A, respectively). The output spiking of the 509 GCs in the network (blue spheres Figure 7G) was recorded over a 30 ms time window, corresponding to the synaptic integration time of GCs (Figure S4B; Schwartz et al., 2012). For each MF input pattern, the number of spikes was calculated for each GC, and this was expressed as a vector for the GC population (blue barcode in Figure 7H). To quantify network performance, we used sets of N = 1,024 MF input patterns, which was the largest number achievable with the computational resources available.

Since direct calculation of the Shannon information between input and output spike trains was computationally intractable, we reduced the dimensionality of the output space by cascading the network with a classifier (called the decoder), which labeled the output spike count vectors as belonging to one of N classes (Experimental Procedures). The network and decoder constituted a communication channel that mapped N input patterns to N output classes, for which we calculated the mutual information (MI) assuming a flat prior over inputs. This fixed the maximum MI achievable as log_2_(1,024) = 10 bits. Unlike for the UBN model, Shannon information in the biologically detailed model does not correspond to the GC population entropy because of the noise introduced by the random spike trains. The network transformation was quantified by calculating the GC population sparseness (Vinje and Gallant, 2000). This measure is analogous to 1-*p(GC)* for the binary case, enabling comparison of the binary and spiking models. The average output sparseness provided a single measure of GC population sparseness across *p(MF)*.

### Best-Performing Synaptic Connectivity for Lossless Sparse Encoding in Biologically Detailed Spiking Network Models

**Figure 8:**
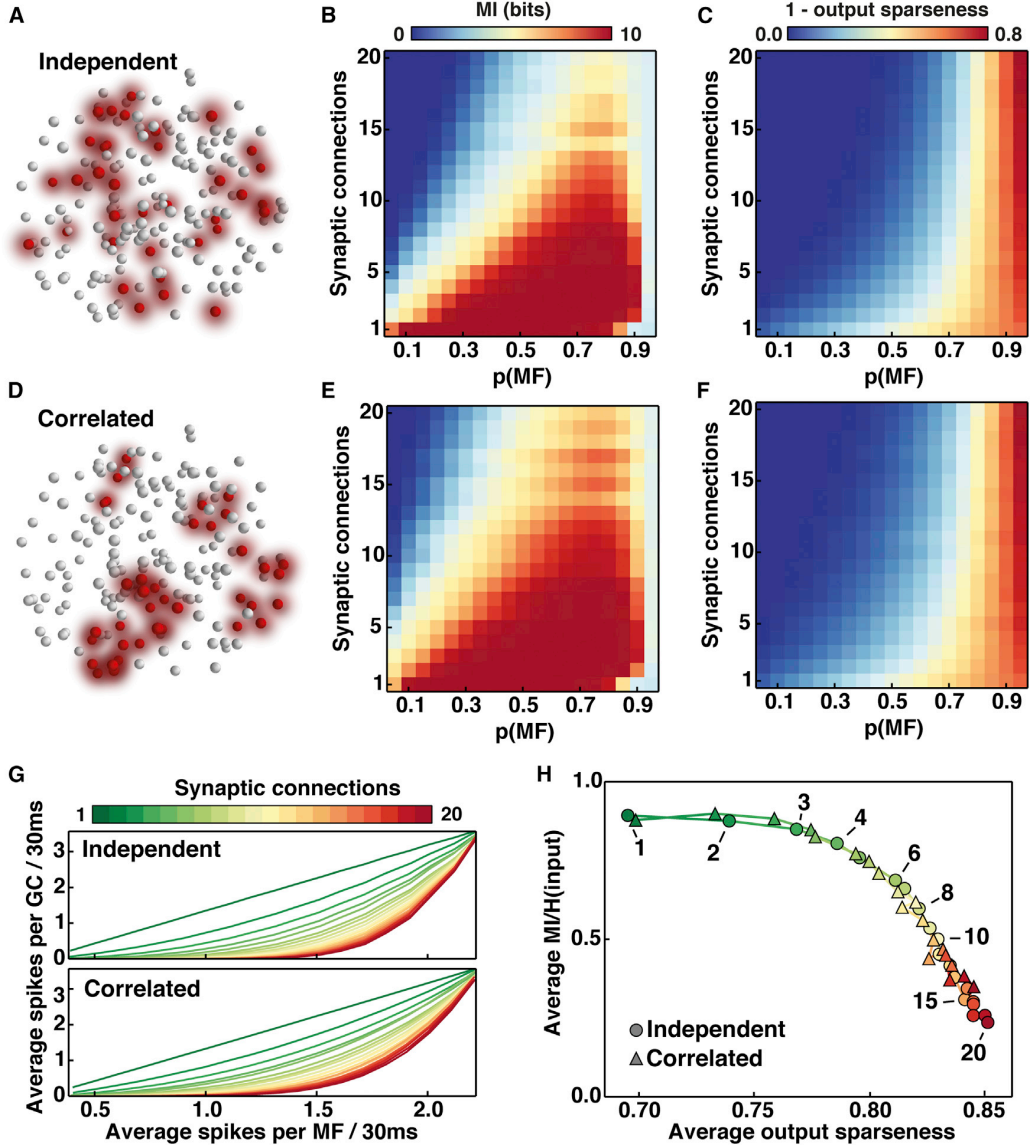
Sparse Encoding in Biologically Detailed Spiking Network Models with Different Numbers of Synaptic Connections per Granule Cell (A) Visualization of independent mossy fiber (MF) inputs in the local granule cell (GC) layer network model with active MFs in red and inactive MFs in white, for an example random activation pattern. (B) Mutual information (MI) encoded by the GC population for 1,024 uncorrelated input patterns across the full range of MF activation probability *p*(*MF*) in biologically detailed spiking networks with different numbers of synaptic connections per GC (*d*). (C) Same as for (B) but for 1-average output sparseness (analogous to *p(GC)* in UBN model). (D, E, and F) Same as for (A), (B), and (C) but for a set of 1,024 spatially correlated patterns, where neighboring MF inputs were activated in groups of five. (G) Same as for (B) and (E) but visualized as a line plot to show the relationship between average spikes per GC and average spikes per MF in a 30 ms window across all values of *p(MF)*, for networks with different *d*. (H) Relationship between average MI (normalized by the MF input entropy) and average output sparseness (across all values of *p(MF)*) for spiking networks with different *d* (color code) for independent (circles) and spatially correlated (triangles) inputs.

We first examined how effectively spiking network models with different synaptic connectivity transmitted independent MF patterns (Figure 8A). Networks with few synaptic connections per neuron were most effective at transmitting information across the widest range of MF input activity, defined as the fraction of MF inputs active (*p(MF)*) (Figure 8B). Indeed, networks with few inputs recovered almost all of the maximum of ten bits, which is remarkable, given the noisy nature of the encoding and that GC spikes were only decoded over 30 ms. However, for networks with larger numbers of synaptic connections per neuron information transmission performance decreased across large regions of *p(MF)*. Reducing or extending the window over which spikes were decoded or altering the firing rates of active MFs shifted the dependence of MI on connectivity, but the overall relationship remained the same (Figures S4 and S5). These results show that biologically detailed spiking networks with few synaptic connections per neuron are most effective at transmitting information, as predicted from the UBN model.

The sparseness of the GC population, averaged over *p(MF)*, increased as the number of MF inputs per GC increased (Figure 8C). Thus, the network connectivity-dependent trade-off between information transmission and sparsification is also present in spiking models. This arose because as the number of synaptic connections per GC increased, the network I-O relationship became highly nonlinear, reducing the average number of spikes per GC to levels well below the average numbers of spikes per MF (Figure 8G). Although encoding in this region was sparse, so few GCs were activated that information transmission was compromised. To find the best-performing biological network configurations, we plotted the relationship between the average fraction of information recovered by the GCs versus average sparsification performed by each network (Figure 8H). Networks with two to seven synaptic connections per GC provided the best solution for performing sparse lossless encoding, with four inputs performing particularly well, supporting the predictions from our simplified analytical approach.

Spatial correlation in MF input activity is likely to occur in real cerebellar networks and may vary from region to region, due to variations in the numbers of MFs arising from different origins (Huang et al., 2013). Since one of the central assumptions in our simplified analytical approach was that all MF inputs were independent, we examined how spatial correlations in the MF activity patterns affected information transmission and sparsification in spiking networks. Introduction of a pronounced spatial correlation in the input, where groups of five neighboring MFs are activated (Figure 8D), marginally increased the information transmission performance over all networks (c.f. Figures 8B and 8E). This is due to GCs in a particular region receiving a larger fraction of active MF inputs and thus having a greater chance of generating a spike. Output sparseness of the GC population was little affected by spatial correlations in the MF input activity (cf. Figures 8C and 8F). Thus, spatial correlations in MF activity only subtly shift the trade-off between information transmission and sparsification in biologically detailed spiking networks.

Finally, we tested whether adding network-activity-dependent inhibition to the physiological level of tonic inhibition improved performance of our spiking networks, as predicted from the UBN model. To do this, we scaled the tonic inhibitory conductance as a function of *p(MF)*, from the experimentally measured tonic level. This reduced information transmission in networks with larger numbers of synaptic connections (Figure S6A). However, for networks with few synaptic connections per neuron, information transmission was preserved and GC activity was further sparsened. Biologically detailed networks with three to five synaptic connections, tonic inhibition, and a modest network-activity-dependent inhibition (NADT = 0.3) performed lossless sparse encoding better than tonic inhibition alone (Figure S6C), again supporting the predictions of our binary model. Interestingly, all three of these features are characteristic properties of the cerebellar GCL, suggesting that both the connectivity and the inhibition properties are tuned to enable robust lossless sparse encoding over the widest range of MF excitatory drive.

## Discussion

We have explored the relationship between the structure of feedforward networks and their ability to transmit information and transform it into a sparse representation, which are both essential for pattern separation. By combining quantitative anatomy of the cerebellar input layer and a full information theoretic treatment of uniform binary network models, we show that the extent of the synaptic connectivity in feedforward networks sets the trade-off between information transmission and sparse encoding. Networks with two to seven synaptic connections per output neuron perform lossless sparse coding over the widest range of input activity. Structurally and functionally detailed spiking network models with synaptic inputs, neuronal properties, and tonic inhibition constrained to experimentally measured values confirmed that the properties of the cerebellar input layer are particularly well suited for performing lossless sparse encoding. Our results therefore provide a computational explanation of why the most numerous neuron in the brain of vertebrates receives an average of four excitatory synaptic inputs.

### The Relationship between Feedforward Network Structure and Function

To understand how synaptic connectivity affects network function, it is first necessary to understand why information transmission and sparse encoding are competing functions. Although a local network of 500 GCs could potentially encode an astronomical number of MF input patterns (i.e., 2^500^ for a binary network), when GC activity is reduced to low levels the capacity of the network to encode patterns shrinks considerably. Our results show that the balance between information transmission and the sparseness of the encoding is set by the extent of synaptic connectivity. Increasing the number of synaptic inputs required to reach firing threshold provides a sparser output representation, but if the probability of GC activation becomes too low, GCL encoding capacity falls and information is lost. By contrast, if the GC network capacity is much larger than the number of MF patterns to be encoded, the GC population activity could be reduced to a sparser representation, thereby saving energy (Attwell and Laughlin, 2001) and improving pattern separation (Tyrrell and Willshaw, 1992).

Results from our biologically constrained spiking networks confirmed that few excitatory synaptic connections per neuron and a high level of inhibition provide a highly effective trade-off between information transmission and sparse encoding. The fact that the synaptic connectivity of the best performing networks match that found in the cerebellar input layer suggests that the GCL structure is optimized for transforming MF input patterns into a higher dimensional sparser code, without information loss. Our results extend classical work on the relationship between cerebellar structure and function (Albus, 1971, Kanerva, 1988, Marr, 1969), by showing that the synaptic connectivity between MFs and GCs is a major determinant of information transmission and sparse encoding in this brain region.

### Relationship between Mossy Fiber Activity and Granule Cell Layer Properties

Since the cerebellum receives dynamic patterns of sensory-motor inputs via the MF system, the ability to perform lossless sparse encoding over a wide range of MF excitatory drive is likely to be crucial. Indeed, in vivo recordings show that MFs exhibit a wide range of activity, with those signaling rapid discrete sensory events exhibiting high-frequency bursts and relatively quiescent periods (Rancz et al., 2007), while those that convey slower continuous sensory variables, such as joint angle and head velocity, typically fire continuously at 10–100 Hz (Arenz et al., 2008, van Kan et al., 1993). The spatial patterns of MF activation are also likely to be highly diverse. Although MF innervations of the GCL exhibit a large-scale fractured map topology (Shambes et al., 1978), the MFs that innervate an individual GC typically arise from different precerebellar nuclei (Huang et al., 2013), suggesting that MF activation is rather spatially independent and that a key function of the GCL is to combine information from different modalities. However, MF inputs onto individual GCs in vermal areas that encode limb movement carry highly correlated information (Jörntell and Ekerot, 2006). Our results show that the synaptic connectivity found within the GCL can losslessly transform a wide range of MF excitatory drive into a sparse GC population code, even when spatial correlations in MF activity are present. Moreover, the broad bandwidth of MF-GC signaling (Saviane and Silver, 2006) also enables certain sensory stimuli, such as whisker deflection, which generate high-frequency MF bursts (e.g., 700 Hz), to be relayed through the GCL (Rancz et al., 2007). Thus, our results suggest that the GCL acts as a general purpose sparse encoder of rate-coded MF inputs that has the flexibility to respond rapidly to urgent stimuli.

### Determinants of Network Encoding Capacity

Our results show that the encoding capacity of noise-free binary networks is sufficiently large to encode all patterns that an animal could possibly encounter during its lifetime. On the other hand, we show that noisy biologically detailed spiking networks can comfortably encode 1,024 rate-coded MF input patterns, assuming GC spikes are integrated over 30 ms. While the number of patterns that a real local GCL network encodes falls between these two values, it may vary widely across cerebellar regions because encoding capacity depends on MF firing rates (Figure S5), correlations in MF activity, the properties of inhibition (Figure S6), and the time window over which GC firing is integrated (Figure S4). These considerations suggest that the encoding capacity of a local GCL network will depend strongly on the properties of the MF inputs it receives.

Another way to increase the encoding capacity is to increase the number of local networks engaged. In vivo recordings from GCs in mouse vestibular cerebellum indicate that ∼400 MF-GC synapses are required to encode head velocity at the precision observed in man (Arenz et al., 2008), suggesting that multiple local GCL networks are involved. Indeed, MF axons, which form ∼20 en passant synaptic rosettes (Eccles et al., 1967, Sultan, 2001), enable GCs in neighboring local networks to sample the same MF signals. The idea that many GCs are required for sensory representations is supported by the finding that markedly reducing the number of functional GCs induces deficits in consolidation of motor learning (but concomitant changes in long-term plasticity could also contribute to these effects; Galliano et al., 2013). These observations are consistent with the notion that multiple local GCL networks are involved in certain sensory-motor tasks and that the large MF to GC divergence found in the cerebellum is required for efficient encoding.

### The Properties of Inhibition and Encoding Capacity

Our results show that the physiological level of tonic GABA_A_R-mediated inhibition (Brickley et al., 1996) provides a robust solution for performing lossless sparse encoding. Recent in vivo recordings show that tonic inhibition dominates other forms of inhibition in GCs, accounting for 98% of the inhibitory charge (Duguid et al., 2012). This sets a relatively high threshold so that simultaneous activity from three or more rate-coded MF inputs are typically required to reach GC firing threshold (Jörntell and Ekerot, 2006, Schwartz et al., 2012). The importance of a high GC spike threshold to cerebellar function is reinforced by the finding that when tonic inhibition was eliminated in GABA_A_α_6_ knockout mice, two-pore K^+^ channels were upregulated, thereby maintaining threshold at a high level (Brickley et al., 2001). However, knocking out transporters has been more effective in modulating GC threshold. Deletion of the GABA transporter GAT1 increased tonic inhibition in GCs by 4-fold and was associated with tremor and ataxia (Chiu et al., 2005). Our results suggest that information loss could have contributed to these behavioral effects. Lowering GC spike threshold by selectively deleting the KCC2 chloride transporter in GCs, which our results would suggest reduces the sparseness of encoding and thus pattern separation, impairs learning consolidation (Seja et al., 2012). Thus, our models of GCL function provide insights into how alterations in the level of tonic inhibition could impair cerebellar function and why GC spike threshold is tightly regulated by homeostatic mechanisms.

When network-activity-dependent inhibition was added to tonic inhibition, it further sparsified GC encoding without loss of information. Our results therefore support previous proposals that Golgi cells aid sparse coding by controlling the gain to the GCL (Albus, 1971, Marr, 1969, Schweighofer et al., 2001). Although weaker, the phasic and spillover components of Golgi cell-mediated inhibition (Rossi et al., 2003) may also contribute to temporal pattering, which could perform temporal sparsening of GC spikes, time slicing (D’Angelo and De Zeeuw, 2009) and introduce delays that are important for learning temporal operations such as eyeblink conditioning (Medina and Mauk, 2000) and signal cancellation (Kennedy et al., 2014). However, the longer temporal delays during signal cancellation are mediated by unipolar brush cells (Kennedy et al., 2014), which form short-range intrinsic MFs that could increase spatial correlations in the vestibular cerebellum where they are more numerous. Indeed, regional variations in the origin of MF inputs, the presence of UBCs and synaptic plasticity within the MF-GC-Golgi cell circuit could tune the spatiotemporal transformation that specific GCL “modules” perform. Our results show that few synaptic connections per GCs provide a robust structural framework that enables a wide range of MF activity patterns to be transmitted and sparsified efficiently.

### Synaptic Connectivity of the Cerebellar Input Layer Is Evolutionarily Conserved

The cerebellum is an ancient brain structure that arose in the early vertebrates. In terms of numbers, cerebellar GCs dominate the vertebrate CNS, making up more than half of all the neurons in the human brain (Williams and Herrup, 1988). Remarkably, the morphology of cerebellar GCs is conserved across a wide range of species including fish, amphibians, reptiles, and mammals (Llinás, 1969, Wittenberg and Wang, 2007), demonstrating that it has been evolutionarily conserved. In mammals, the observed range is two to seven dendrites (and thus MF inputs) per GC (Palkovits et al., 1972) with four per cell being the most common configuration. The strikingly similarity between the synaptic connectivity in the cerebellar GCL and the feedforward networks that provide the best trade-off between information transmission and sparsification provides a functional explanation for why the characteristic dendritic morphology of cerebellar GCs has been conserved for hundreds of millions of years.

### Comparison of the Structure of the GCL to Other Networks

The cerebellar GCL is not the only example of a network that performs sparsification and has few synaptic connections per neuron. GCs in the dorsal cochlear nucleus and deep GC in the electrosensory lobe (ELL) of the electric fish have one to four synaptic inputs, averaging to three in the ELL (Kennedy et al., 2014, Mugnaini et al., 1980, Zhang et al., 2007). Kenyon cells in the mushroom body of the fly receive an average of seven synaptic inputs from olfactory projection neurons (Caron et al., 2013). Indeed, expansion recoding in the insect mushroom body has other similarities to the cerebellar input layer (Laurent, 2002), including random connectivity (Caron et al., 2013), and inhibitory interneurons that facilitate sparsification (Papadopoulou et al., 2011), enhancing pattern separation and enabling the discrimination of similar odors (Lin et al., 2014). These examples suggest that other brain structures may have converged on a similar feedforward network structure for performing lossless sparse encoding.

If few synaptic connections provide an evolutionary advantage, then why don’t other input layers exhibit a similar structure to the cerebellar GCL? Although we cannot provide a definitive answer, the distinct functions performed by different brain regions provide some hints. Spiny stellate cells in layer 4 of neocortex receive many more synaptic inputs than GCs, but these are predominantly recurrent excitatory connections, which amplify thalamic synaptic input that display strong short-term depression (Lien and Scanziani, 2013). Moreover, nonlinear NMDAR spikes in the dendrites of spiny stellate cells also amplify synaptic inputs (Lavzin et al., 2012). Recurrent connections introduce loops that can support attractor states, intrinsic activity, and complex nonlinear dynamics (Buonomano and Maass, 2009) and aid receptive field formation and feature extraction (Somers et al., 1995). These synaptic, cellular, and network properties appear tuned to detect features and amplify novel stimuli, producing gradually fading memory traces that enable sensory input to be combined with recent experience (Buonomano and Maass, 2009). These sophisticated operations may explain why the structure of the neocortical input layer is more complex than cerebellar input layer, which lacks recurrent excitatory connections.

### Experimentally Testable Predictions

Our results make a number of predictions that could potentially be tested experimentally: (1) GC population activity is sparser than sustained MF activity within a local region; (2) information is conserved within local GCL networks; (3) reduction of tonic inhibition impairs pattern separation, while elevation impairs information transmission; (4) network-activity-dependent inhibition improves lossless sparse encoding; and (5) increasing the number of MFs inputs per GC aids sparsification but impairs information transmission. However, experimental validation is complicated by the need to measure spike trains from local populations of MFs and GC in awake behaving animals and potential compensatory effects associated with genetic changes. Nevertheless, work combining genetic manipulations and motor learning consolidation (Galliano et al., 2013, Seja et al., 2012) and developments in fast 3D imaging technologies (Fernández-Alfonso et al., 2014) look encouraging.

## Experimental Procedures

### Measurement of Granule Cell and Glomerular Density

Four 30-day-old Sprague-Dawley rats were deeply anesthetized and perfused with 4% paraformaldehyde in 0.1 M phosphate buffer and 40-μm-thick sagittal sections of cerebellum were prepared. Cerebellar glomeruli were labeled with anti-Kv4.2, anti-VGAT and anti-VGLUT1, or anti-GLAST primary antibodies and Alexa 488, Cy5, or CY3 labeled secondary antibodies (Supplemental Information) and visualized with a confocal scanning microscope. The two-way dissector method was used to determine GC density within GCL and tissue shrinkage was taken into account. Density of glomeruli was calculated from the mean volume of glomeruli and the mean volume of GCL occupied by glomeruli.

### Construction of the Uniform Binary Network Model

The UBN model was constructed with connectivity statistics as close as possible to the anatomically constrained local GCL network model. The UBN model is formally equivalent to a random bipartite graph consisting of two disjoint sets of nodes (inputs, representing MF rosettes, and outputs, representing GCs), with each output node connected to a fixed number of randomly chosen input nodes (representing the number of synaptic connections per GC). The relative fixed binary threshold was implemented across networks with different numbers of synaptic connections per GC (*d*) by setting the minimum GC threshold to *ceiling[0.75* × *d]*, where ceiling rounds noninteger values up to the nearest integer. NADT was modeled as a dependence between the threshold of the GCs and *p*(*MF*), using a piecewise constant function, monotonically increasing from 1 to *d* over a fraction of the total *p(MF)* range given by 1/NADT (e.g., raising from 1 to *d* between *p(MF)* = 0 and *p(MF)* = 1/2 for NADT = 2).

### Information Theoretical Analysis of the Uniform Binary Network Model

Because the UBN model is noise-free the Shannon Information between the events and the state of the GC population is the GC population entropy (*H*),

(1) $$H=-\sum_{k=1}^{G}p\left(k\right)\log_{2}\left[p\left(k\right)\right]$$

where *p*(*k*) is the probability of the *k*^th^ of *G* unique GC patterns caused by events. We developed a mathematical technique that enabled us to directly calculate average entropy across network instantiations (see derivation of Equation 29; Appendix, Supplemental Information) even for large numbers of GC patterns. Computation time using our analytical method is independent of the number of events.

### Biologically Detailed Spiking Network Simulations

For spiking networks, we used the anatomically constrained local GCL network model. GCs were modeled using a conductance-based integrate-and-fire model whose parameters were set to previously published experimental averages (Rothman et al., 2009, Schwartz et al., 2012; Table S3). AMPAR- and NMDAR-mediated synaptic conductances were fitted using swarm intelligence techniques (Supplemental Information) to measured EPSCs (Rothman et al., 2009), with short-term plasticity modeled as in Tsodyks et al. (1998). We used 1,024 MF patterns, since this was the maximum possible with the computational resources available: simulations and analysis of Figures 8, S5, and S6 required more than one million 2 GHz core hours. Cell and synaptic models in NeuroML2/LEMS format (Gleeson et al., 2010) and links to the simulation and data management code are available on the Open Source Brain (http://www.opensourcebrain.org/projects/granule-cell-layer-piasini-2014).

### Analysis of Spiking Network Data

Mutual information was calculated between the set of N input patterns and N output network activity classes, obtained by performing an appropriate tessellation of the output space, as this was the smallest number of classes that allowed for full recovery of information (Supplemental Information). Undersampling bias in the MI estimate (Treves and Panzeri, 1995) was accounted and corrected for. Population sparseness was defined as

$$S=\left(C-\frac{{\left(\sum_{i=1}^{c}r_{i}\right)}^{2}}{\sum_{i=1}^{c}r_{i}^{2}}\right)/\left(C-1\right)$$

(Vinje and Gallant, 2000) where *C* is the number of cells and *r*_*i*_ is the spike count of cell *i*.

## Author Contributions

G.B. developed the UBN model and analytical methods, with refinements from E.P. and R.A.S. E.P. built the spiking model and software to manage the numerical simulations and performed analysis. A.L. and Z.N. performed experiments and the quantitative anatomical analysis. R.A.S. conceived and supervised the project and wrote the manuscript, with contributions from all authors.

## Supplemental Information

Document S1. Supplemental Experimental Procedures, Figures S1–S6, and Tables S1 and S2

Document S2. Article plus Supplemental Information
