# Supplementary material for: Network Structure within the Cerebellar Input Layer Enables Lossless Sparse Encoding
Source: Neuron. 2014 Aug 20;83(4):960–74. doi: 10.1016/j.neuron.2014.07.020 (PMC4148198; doi:10.1016/j.neuron.2014.07.020)
Supplement: Document S1. Supplemental Experimental Procedures, Figures S1–S6, and Tables S1 and S2 [file mmc1.pdf]

Neuron, Volume *83*

Supplemental Information

Network Structure within the Cerebellar Input Layer Enables Lossless Sparse Encoding

Guy Billings, Eugenio Piasini, Andrea Lőrincz, Zoltan Nusser, and R. Angus Silver

1 Supplemental data

Figure S1, related to Figure 1

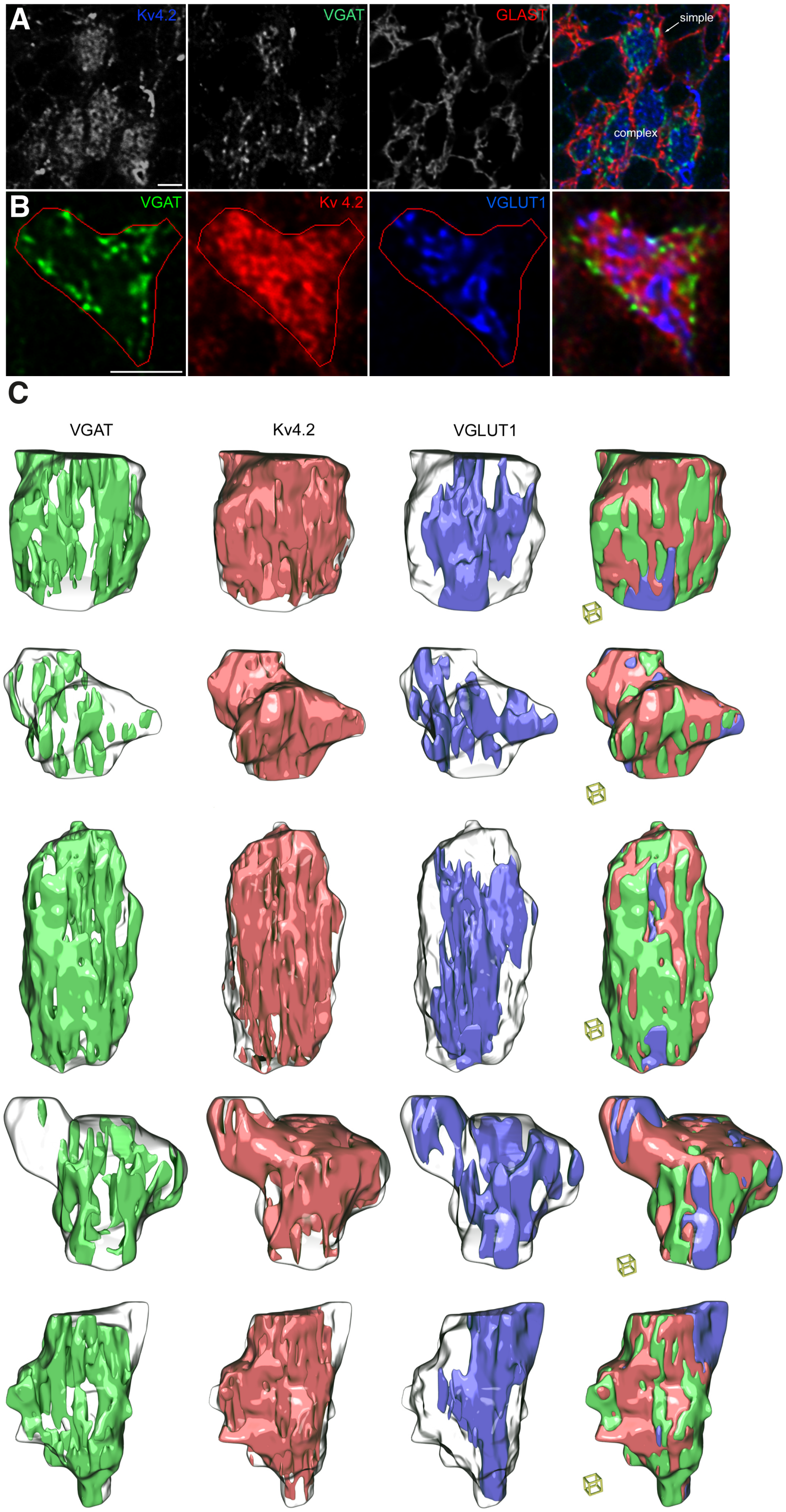

Figure S1, related to Figure 1: Molecular identification and reconstruction of cerebellar glomeruli. A: Immunolabelling for Kv4.2, VGAT and GLAST reveals a complex structure assembled from several simple glomeruli. B: A single confocal section of a simple glomerulus. Within the glomerulus a single VGLUT1 immunopositive mossy fiber synaptic rosette (blue) is present in the central position and it is surrounded by Kv4.2 subunit-immunolabeled granule cell dendrites (red) and by VGAT immunoreactive Golgi cell axon terminals (green). Scale bars: 5 µm. C: Five representative glomeruli reconstructed from confocal image stacks. Inside the glomerulus VGAT immunoreactive Golgi cell axon terminals (green) and Kv4.2 subunit-immunolabeled granule cell dendrites (red) enclose a single VGLUT1 immunopositive mossy fiber synaptic rosette (blue). Each edge of the cube: 1 μm.

Figure S2, related to Figure 2

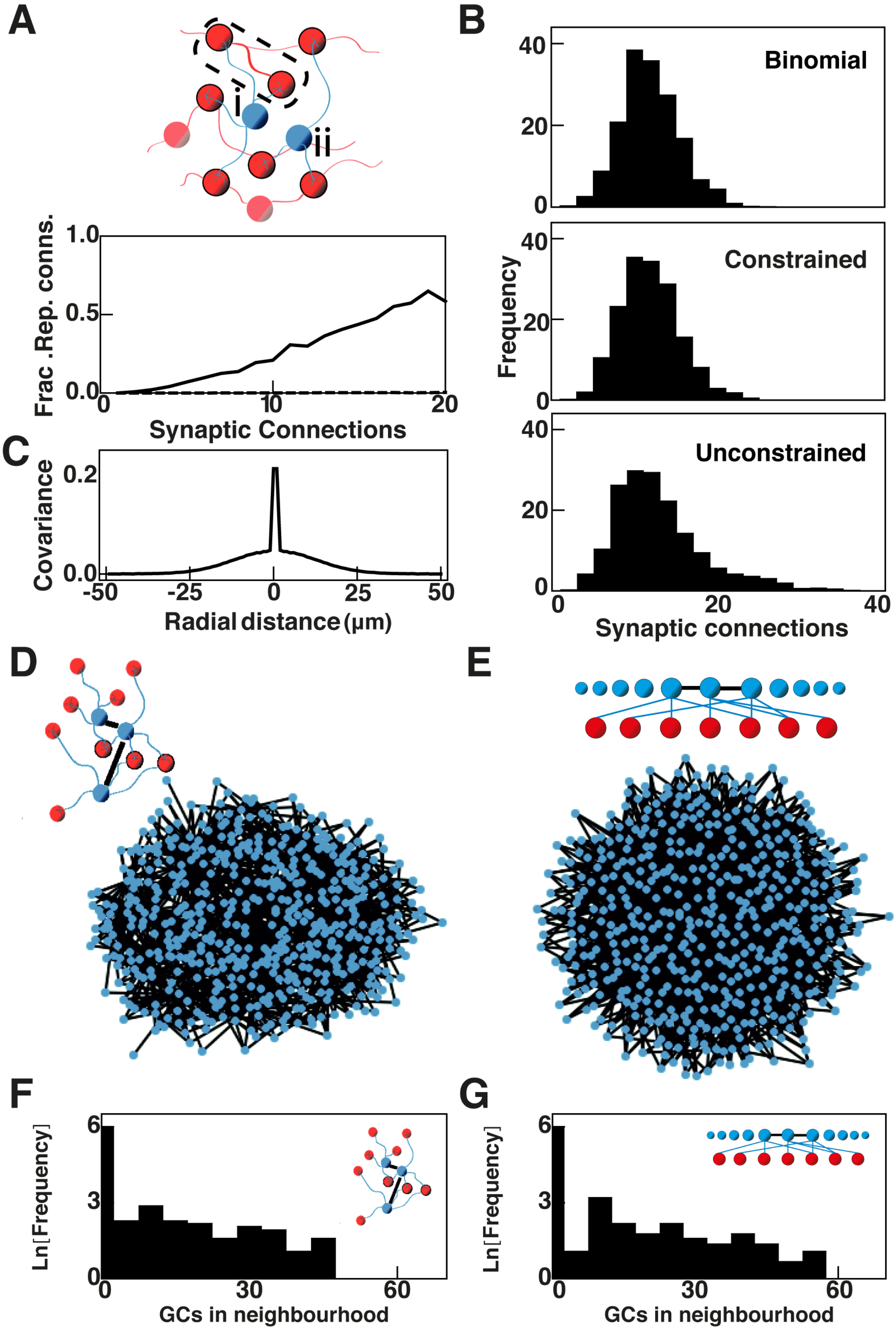

Figure S2, related to Figure 2: Connectivity constraints in the anatomical model produce a binomial distribution of the number of synaptic connections per mossy fiber rosette and biologically plausible dendrite lengths. A: Top: schematic diagram illustrating how multiple synaptic rosettes (glomeruli; red balls) are associated with each mossy fibers (MF; red lines) and distributed in 3D space. These rosettes/glomeruli may in principle be on the same fiber (eg. enclosed with dashed box) or on independent fibers. GCs (blue balls) connect to a subset of these (highlighted balls) within range of their dendrites (blue lines, known to be $\sim 15\mu m$ long on average). For each GC, there are several ways in which connectivity can give rise to non-uniform numbers of connections with distinct MFs including the case where two dendrites contact a single MF synaptic rosette. Here we illustrate i) A GC (blue) with $d = 4$ synaptic inputs, each made onto a different dendrite, but connects to a single MF via two separate synaptic rosettes and ii) Given some connectivity constraint (e.g. dendrite length limit) GCs can fail to connect all dendrites to d glomeruli. Bottom: Fraction of the GCs that do not have d independent inputs as a function of the number of synaptic connections per GC in a model with random connectivity (solid black line) and the same fraction in the model having connectivity constraints (see Supplemental Experimental Procedures) so as to avoid these effects (dashed black line) B: Binomial distribution with a mean of 12, which matches the expected number of synaptic connections per MF in the anatomical model (top), distribution of number of synaptic connections per MF synaptic rosette in anatomical model with constrained random connections (middle), distribution of number of synaptic connections per MF in anatomical model with unconstrained random connections (bottom). C: Mean covariance of GC activity versus distance from the center of the sphere in a *binary local GCL model* (where GCs were represented by binary linear threshold units, and connectivity was anatomically constrained) with 4 MF connections per GC. D: The neighborhood graph, formed by taking GCs as nodes (blue circles) and adding edges (black lines) between those GCs when the GCs share any MF inputs (red circles). The neighborhood graph for the anatomically constrained local GCL network is shown below. E: As D but for the uniform binary

model. More uniform distribution of nodes in this graph indicates a more even sharing of inputs in the uniform binary model. F: The distribution of the natural log of the number of neighborhood sizes indicates the number of other GCs that each GC directly shares inputs with. G: As F but for the bipartite graph. In the uniform binary model, GCs tend to have a larger local neighborhood.

Figure S3, related to Figure 2

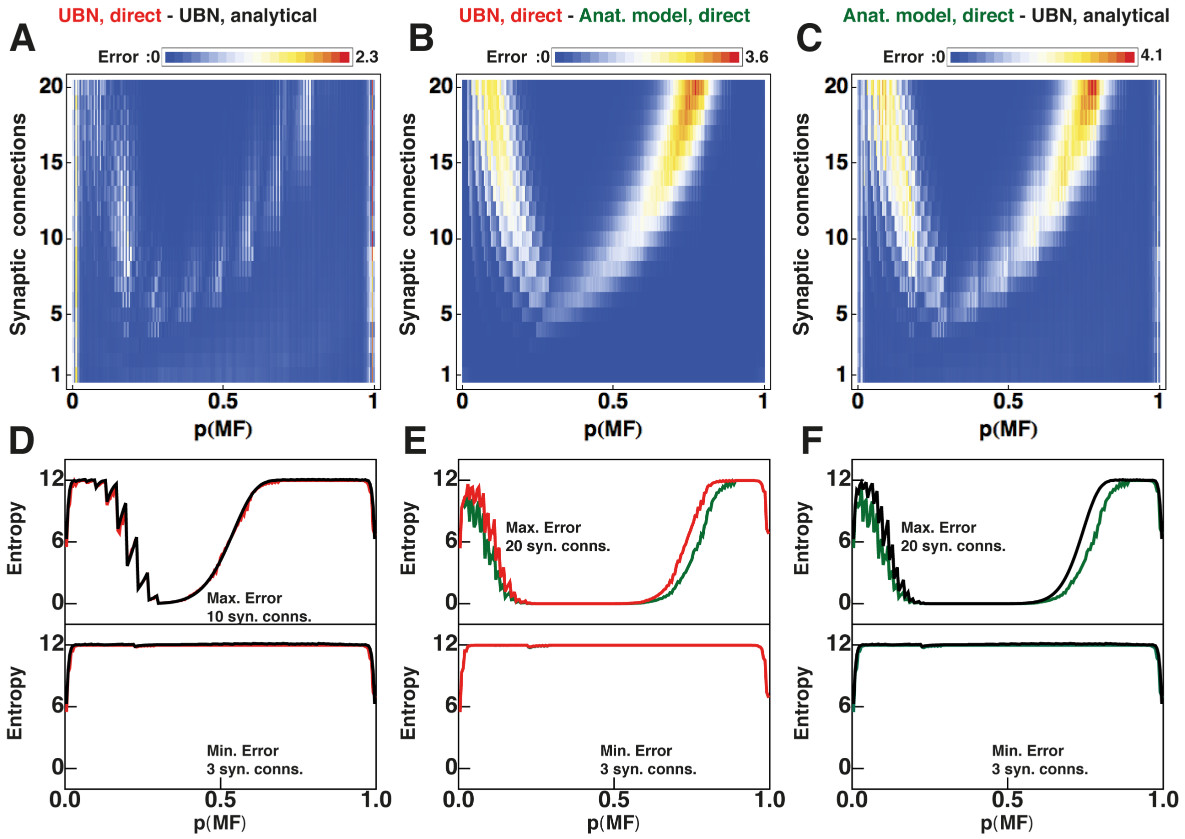

Figure S3, related to Figure 2: Comparison of analytical method for estimating granule cell entropy to direct numerical evaluation. A: Absolute difference ($\langle \Delta H \rangle$, labeled 'Error') of the direct and analytical estimates for the granule cell (GC) entropy encoded from 4000 events in the uniform binary network (UBN) model. Plot shows difference as a function of the number of synaptic connections per GC and the mossy fiber (MF) activity level $p(MF)$. B: As A but for the discrepancy in the direct estimate for the 4000 event entropy between the UBN and the anatomical local GCL model. C: Discrepancy between the direct estimate for the anatomical model and the analytical calculation for the UBN. Note different color scales. D: Top: Entropy encoded after 4000 events for case with 10 synaptic connections, which has the maximum error in A. Black line is entropy determined by our analytical method, red line is data for explicitly simulated UBN. Bottom: Comparison for lowest error case when the network has 3 GC dendrites. E: As D but for comparison of the direct estimate in the UBN (red) and the anatomical model (green); maximum and minimum error in B occurring for 20 GC dendrites

(top) and (respectively) 3 GC dendrites (bottom). F: As E but for comparison of the direct estimate in the anatomical local GCL model (green) and the analytical calculation for the UBN (black) These results show that the errors in our approach are modest with the largest error arising from the simplification of the anatomical local GCL model to a bipartite graph. Data shown is for networks with network activity dependent GC threshold (NADT) = 3.

Figure S4, related to Figure 7

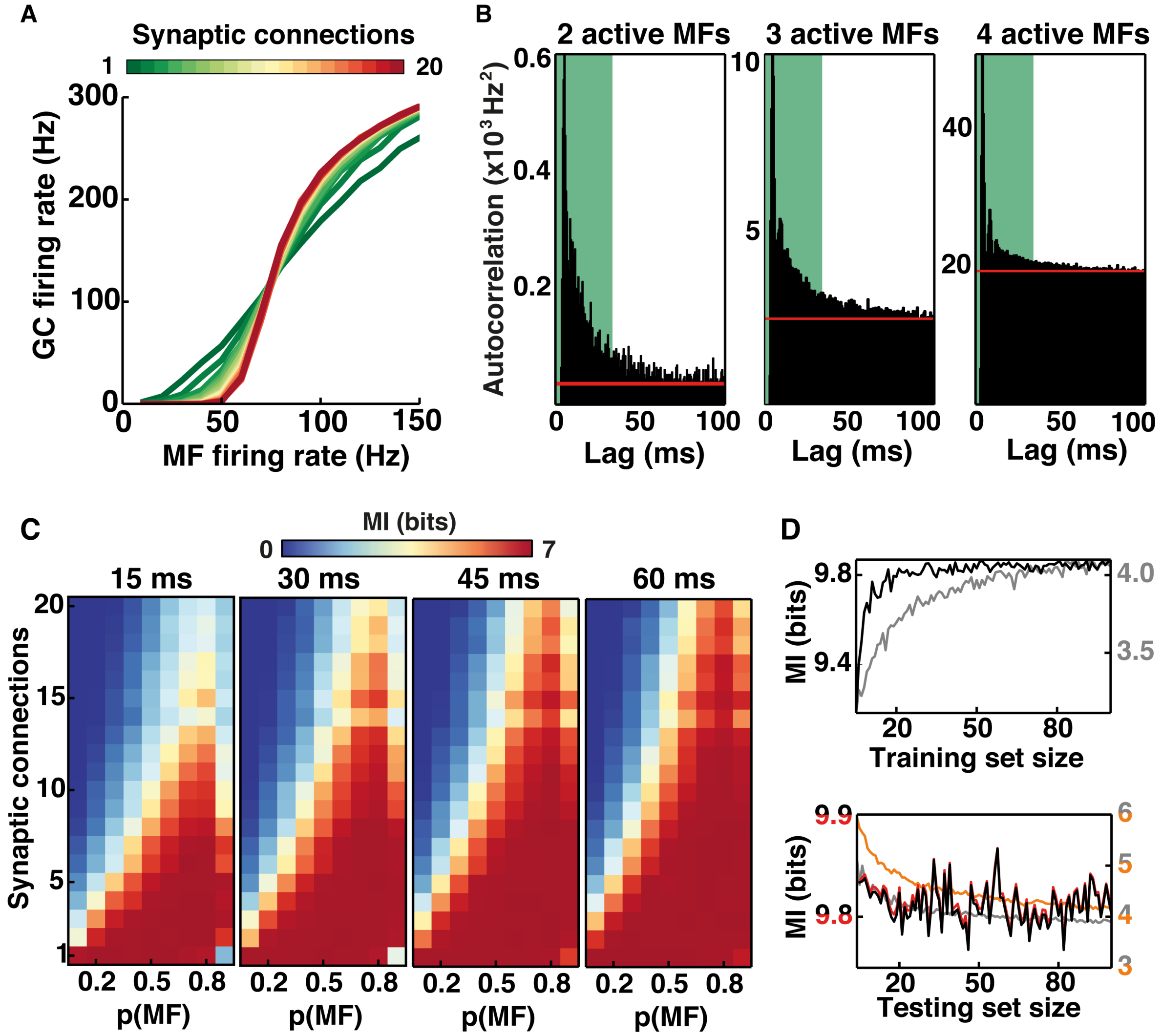

Figure S4, related to Figure 7: Effect of changing the number of synaptic connections on the granule cell input-output relationship, time window of synaptic integration in granule cells and the dependence of encoding on the integration window of the decoder. A: rate-coded input-output curve for the spiking granule cell (GC) model with a variable number of mossy fiber (MF) synaptic inputs per GC. B: autocorrelation of the spiking GC model output (with 4 synaptic inputs) for 2, 3 or 4 active inputs (the case of 1 active input is not shown as the resulting firing rate is very low). Red line: constant value of the autocorrelation of a Poisson point process with the same mean intensity. Green area: 30ms time window. C: Mutual information (MI) for 128 patterns, estimated for different lengths of the time window over which network activity is recorded.

D: Top: MI as a function of the number of repetitions per pattern used to train the decoder, for 1024 MF patterns, 4 synaptic connections and $p(MF)$=0.1 (gray) or $p(MF)$=0.5 (black). Note the different scales. The network performs poorly for $p(MF)$=0.1, transmitting only a fraction of the information present in the input, so this is a particularly stringent test for the performance of the decoder. Bottom: MI as a function of the number of repetitions per pattern used to estimate it, showing the magnitude of undersampling bias and the effect of our chosen bias correction technique. Grey and orange: 4 synaptic connections, $p(MF)$=0.1, quadratic extrapolation and no undersampling bias correction, respectively. Black and red: 4 synaptic connections, $p(MF)$=0.5, quadratic extrapolation and no undersampling bias correction, respectively.

Figure S5, related to Figure 8

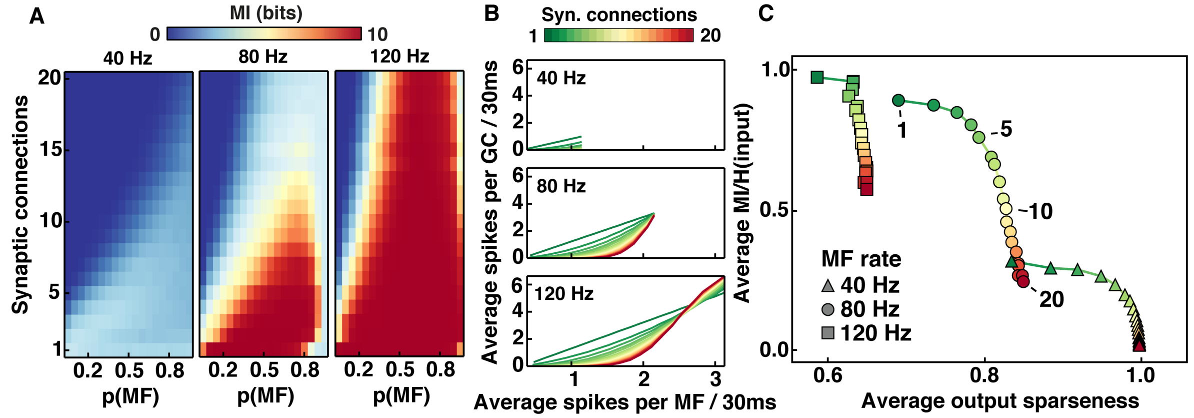

Figure S5, related to Figure 8: Effect of different mossy fiber input rates on spiking granule cell layer network performance. A: Mutual information (MI) for 1024 input patterns for active mossy fibers (MFs) firing at different rates, with tonic inhibition kept fixed at the average experimental value. B: average spikes per granule cell (GC) versus average spikes per MF within a 30 ms window, across all patterns and all values of the probability of MFs being active ($p(MF)$). Different color lines show relationships for networks with different numbers of synaptic connections. C: Average MI, normalized by the input entropy, versus average output sparseness (with averages taken across all values of $p(MF)$) for different active MF rates, parametrized by the number of synaptic connections. Note how, as shown in at the bottom of panel B, there exists a threshold level of MF activity above which the relationship between number of synaptic connections and GC activity is reversed, and the most active networks are those with more connections. This is reflected in panel C, as the networks with many synaptic connections lose the sparsification advantage they have when the inputs are encoded at lower rates, leaving those with fewer connections as an optimal choice for lossless sparse encoding, even though the maximum sparsification attainable is lower.

Figure S6, related to Figure 8

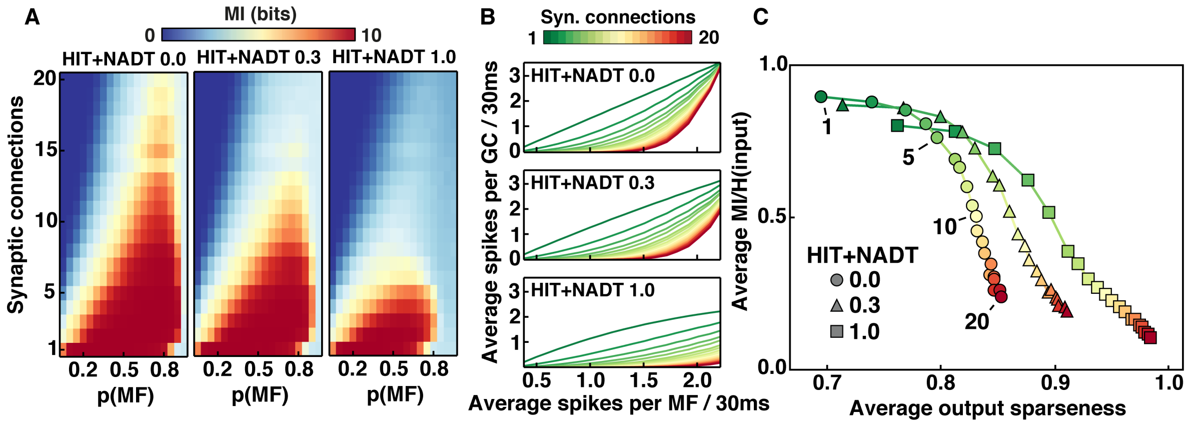

Figure S6, related to Figure 8: Effect of network activity-dependent scaling of tonic inhibition. A: Mutual information (MI) for 1024 patterns of different active mossy fibers (MFs) with different levels of scaling of tonic inhibition in granule cells (GCs) with network activity (analogous to NADT for the uniform binary model) added on top of the physiological baseline (indicated as HIT for consistency with Figure 6 in the main text); heatmap on the left (NADT=0) is included for comparison and represents the baseline case. In these simulations an extra amount of GABA$_A$R-mediated conductance, proportional to the fraction of active MFs ($p(MF)$), was introduced. The NADT parameter is a scaling coefficient for this proportionality relation. For example, for NADT=1 and $p(MF)$=0.6 the GABA$_A$R-mediated conductance will be increased by 60%, whereas for the same $p(MF)$, but NADT=0.3 the increase will be of 20%. B: average spikes per GC versus average spikes per MF across all patterns and all values of the probability of MFs being active ($p(MF)$), parametrized by the number of synaptic connections for the different levels of NADT. C: Average MI, normalized by input entropy, versus average output sparseness (with averages taken across all values of $p(MF)$) for different values of NADT parametrized by the number of synaptic connections. The value of NADT=0.3, which is our rough estimate of how the time-averaged GABA$_A$R conductance contributed by Golgi cells varies with network activity (Ward, Piasini and Silver, unpublished observations), increased the performance for networks with few synaptic connections.

Table S1, related to Figure 1: Size of the cerebellar glomeruli and their components

	GC dendrites (Kv4.2)		Golgi axons (VGAT)		Mossy fiber synaptic rosette (VGLUT1)		Glomerulus		
	Mean ± SD	Range	Mean ± SD	Range	Mean ± SD	Range	Mean ± SD	Range	
Rats	(μm^3)	(μm^3)	(μm^3)	(μm^3)	(μm^3)	(μm^3)	(μm^3)	(μm^3)	N
#5	184.5 ± 37.9	$108.7 - 231.8$	122.2 ± 42.7	$61.1 - 190.3$	96.2 ± 33.7	$45.6 - 144.3$	402.8 ± 106.8	$215.3 - 549.4$	10
#6	250.3 ± 144.8	$123.8 - 496.6$	148.6 ± 68.1	$44.4 - 228.4$	148.5 ± 58.8	$67.6 - 214.4$	547.4 ± 256.8	$235.8 - 939.3$	5
#1	152.6 ± 50.2	$92.9 - 228.2$	112.2 ± 44.4	$36.5 - 181.5$	88.1 ± 20.9	$46.0 - 109.6$	352.9 ± 92.3	$200.7 - 480.2$	9
Mean	195.8 ± 49.8		127.7 ± 18.8		110.9 ± 32.8		434.4 ± 100.9		

Table S2, related to Figure 1: Density of mossy fiber glomeruli

	Rats			
	#5	#6	#1	Mean
GCL occupied by glomeruli (%)	30.6 ± 5.5	26.3 ± 4.9	29.5 ± 4.8	28.8 ± 2.3
Density of glomeruli ($1/mm^3$)	7.5×10^5	4.9×10^5	8.5×10^5	$6.6 \pm 1.5 \times 10^5$

2 Supplemental experimental procedures

2.1 Measurement of granule cell and glomerular density

2.1.1 Tissue preparation and fluorescence immunocytochemistry

Four thirty-day old Sprague-Dawley rats were deeply anesthetized with Halothane and 7% Chloral hydrate (0.4ml), then perfused through the aorta first with saline then with 4% paraformaldehyde in 0.1M phosphate buffer (PB, pH = 7.3) for 12 min. The brains were immediately removed and blocks were cut out from the cerebellum. 40μm thick sagittal sections were cut with a Vibratome (VT1000S, Leica Microsystems, Vienna, Austria) and washed overnight in 0.1M PB. Normal goat serum (NGS, 10%) in Tris-buffered saline (TBS, pH =7.4) was used for blocking, followed by incubations in primary antibodies. Sections for labeling cerebellar glomeruli were incubated in the mixture of guinea pig anti-VGAT (1:500; Calbiochem, Darmstadt, Germany), mouse anti-Kv4.2 (1:1000; NeuroMab, UC Davis, CA), and rabbit anti-VGLUT1 (1:500; Synaptic Systems, Goettingen, Germany) or rabbit anti-GLAST (1:500; gift from Prof. Shigemoto) antibodies diluted in TBS containing 2% NGS and 0.05% Triton X-100. Following several washes, sections were further incubated in the mixture of Alexa 488 goat anti-guinea pig (1:500; Molecular Probes, Leiden, The Netherlands), Cy5 conjugated goat anti-rabbit IgG (1:500, Jackson ImmunoResearch, West Grove, PA) and Cy3 conjugated goat anti-mouse IgG (1:500, Jackson ImmunoResearch) for 2 hours. Sections were mounted on slides in Vectashield (Vector Laboratories, Burlingame, CA).

2.1.2 Image acquisition

Images were taken from parasagittal vermis sections, from the proximal part of lobule VI (lobule VIa). Immunofluorescence labeling was visualized by a confocal scanning microscope (FV1000, Olympus Europe, Hamburg, Germany). Auto-

mated sequential acquisition of multiple channels was used for multi-color images to avoid spectral crosstalk between channels. Series of 25-37 confocal images (800 X 800 voxels) spaced at 0.5, 1 or 3 μm apart were acquired using either a 40X (NA=0.85) or a 60X (NA=1.3) objective lens.

2.1.3 Calculation of the density of granule cells

Granule cells (GCs) were identified by the Kv4.2 immunolabeling, which outlines the somato-dendritic plasma membranes of GCs. The two-way dissector method (Gundersen et al., 1988) was used to determine GC density within GC layer (GCL). The distance between the sections was chosen to be about 30% of the average height of the objects to be counted. The mean diameter of GCs was found to be 6.7 μm, resulting in a real dissector distance of 2 μm. A weak fixation was used to achieve maximal penetration of the antibodies into the tissue. However, such a mild fixation resulted in considerable tissue shrinkage and thickness of the section when mounted on a glass slide and covered with a coverslip. Accordingly, the axial diameter of the GCs was reduced, thus the dissector distance had to be corrected for shrinkage. To evaluate the level of tissue shrinkage we calculated a shrinkage factor (0.57 ± 0.09, n= 3 rats, 3 slices per rat) for individual slices by dividing the thickness of the slice after processing with the slice thickness before processing (40 μm). Indeed, the height of GCs collapsed to 57-59% along the Z-axis. We did not correct for XY direction, as the tissue XY shrinkage was negligible (< 2%).

Pairs of reference and look-up optical sections were randomly selected from the recorded image stacks. Areas for counting were sampled by counting frames (300x300px, 3546.2μm^2) positioned on the images (Figure 1B1 & B2). Cells cut by the acceptance line (green), but not by the forbidden line (red) were included in counting. A cell was counted if the transect from the cell was seen on the reference image but not on the corresponding look-up section. Two counting frames per section (in two-way) and 3 slices per animal were used for calculating

the numerical density of GCs (ρ_{GC}),

$$\rho_{GC} = \frac{\text{total cells counted}}{\text{total area of frames} \times \text{real dissector distance}}.$$

2.1.4 Measurement of the density of cerebellar glomeruli

Mossy fiber (MF) synaptic rosettes, GC dendrites and Golgi cell axon terminals forming a cerebellar glomerulus were identified by, respectively, VGLUT1, Kv4.2 and VGAT immunolabeling (Figure 1C-G; Figure S1). A MF synaptic rosette, which is typically en passant, forms the core of each simple glomerulus. This is surrounded by GC dendrites and by Golgi cell axon terminals. The whole glomerulus is ensheathed by glial processes immunopositive for the glutamate transporter GLAST, Figure S1. Some glomeruli were clumped together forming complex glomeruli. Visualization of the different components showed that they were formed from 2-5 simple glomeruli, Figure S1. Confocal image stacks of simple and in some cases complex glomeruli were acquired at 0.2-0.4 μm axial resolution and were de-convolved by the maximal likelihood estimation algorithm implemented in the Cell P software (Olympus Soft Imaging System, Munster, Germany). Color channels representing VGLUT1 (blue), Kv4.2 (red) and VGAT (green) immunolabeling were binarised and volume calculations were performed in Matlab (MathWorks Inc., Natick, USA-MA). Within the glomerulus the volume of each voxel was weighted by the intensity of corresponding RGB values and volume fractions for the three profiles were calculated, Figure S1. Density of glomeruli was calculated from the mean volume of glomeruli and the mean volume of GCL occupied by glomeruli.

2.2 Anatomically constrained local granule cell layer model

Given the measured ratio of glomeruli to GCs and the number of MF synaptic connections per GC, then the mean number of synaptic connections per MF rosette is fixed. However, in practice the MF rosette degree distribution (i.e. the distribution of the number of synaptic connections per rosette) depends upon the shape of the 3D volume used to construct the model. This occurs because GC soma within the defined volume of the model can connect to glomeruli located outside by virtue of the finite length of GC dendrites. This changes the effective glomerular to GC ratio and also causes the glomeruli outside the GC cell body volume to be sampled less frequently than those within it, affecting the connectivity statistics of the local network. We found that confining the whole network, including all dendrites, within a sphere minimized these effects (e.g. Figure S2). Another problem with constructing networks was that randomly connecting MF rosettes and GCs resulted in some GCs sampling the same rosette twice or the same MF more than once via differing glomeruli (Figure S2A, top). For a network having only 4 dendrites this effect was small (~ 4%) and consistent with experimental observations, but this problem grew as the number of synaptic connections per GC was increased, causing the number of independent MF inputs to depend upon the number of dendrites. We overcame these problems by imposing a constraint on the GC dendrite lengths which, while allowed in principle to assume any value, are as close as possible to $15\mu m$, and by explicitly forcing GCs to have all dendrites connecting to independent MFs (Figure S2A, bottom). This enabled us to generate a spherical network model with a MF rosette degree distribution that was binomial in shape, rather than one with a tail (Figure S2B), as well as a dendrite length distribution having mean $17\mu m$ and mode 15μm, Figure S2C. Indeed, most dendrite lengths were $< 20\mu m$ for 4 dendrites per GC (Figure S2B), as found experimentally (Eccles et al., 1967; Palkovits et al., 1972).

2.3 Uniform binary network model

The structure of the uniform binary network model is formally equivalent to a random bipartite graph consisting of two disjoint sets of nodes with inputs representing MF rosettes and outputs representing GCs, connected by randomly placed edges (synaptic connections), such that each edge joins an input node to an output node. No edges are permitted between two inputs or between two outputs. We applied the added constraint that each output node can only connect to a fixed number of edges, representing the number of synaptic connections per GC. In this manner we constructed a random uniform binary model with connectivity statistics as close as possible to the 3D anatomically constrained local granule cell network model. While the degree distribution of MF rosettes in the random bipartite graph matched the anatomically constrained model, the spatial structure of the connectivity statistics was lost. Because the GCs in the anatomically constrained local GCL model connect by favoring dendrites of around 15μm, they tend to share MFs with nearby GCs, introducing a spatial dependence that does not exist in the more abstract uniform binary network model. In the bipartite graph connections are not influenced by the dimensions of the network, since a particular GC can connect to any MF with equal probability. This is an important assumption in the method of calculating the entropy of the GCs in the bipartite graph (see Appendix below). This can be more clearly seen when we compare connectivity in the anatomically constrained model and bipartite graph by means of their respective neighborhood graphs (Figure S2D,E). The GCL neighborhood graph consists of GCs as nodes with edges between any GCs that mutually connect to at least one MF. Hence it depicts the tendency of GCs to share MFs. The neighborhood graphs were plotted with spring-electric embedding (using the integrated function 'GraphPlot' in Mathematica (Wolfram Research, Champaign, USA-IL), such that each node repels other nodes in a Coulombic manner while exerting an attractive force on its neighbors in the manner of Hooke's law. The graph plotted is the equilibrium configuration of this system.

In 'spring-electric embedding' representations of networks the edges of the graph

are more evenly distributed the more evenly separated the nodes of the graphs are, such that all nodes have an approximately isotropic attraction/repulsion to other nodes. The neighborhood graph for the anatomically constrained local granule cell network model exhibited a flattened spherical shape due to clustering of GCs, caused by the restricted dendrite lengths (Figure S2D). In contrast, the neighborhood graph of the bipartite network had a spherical shape due to the spatially uniform connectivity (Figure S2E). Moreover, the bipartite network contains more GCs that share inputs with large numbers of other GCs (Figure S2F &G). However, the differences between the anatomically constrained local GCL network and the uniform binary model are relatively small because the mean span of the dendrites is comparable to the radius of the network. This resulted in similar information transmission properties when we compared the properties of binary networks constructed with the anatomically constrained and the uniform binary model structures and calculated information with the direct method (see Figure S3). Moreover, the systematic error introduced by differences in network structure overestimates the encodable range of networks having many connections. Hence, correction of the error would serve to strengthen the finding that networks having many synaptic connections per GC encode a smaller range of MF activity (Figure S3F).

2.4 Local granule cell layer network size and calculation of granule cell covariance

We set the network radius to $40\mu m$, which corresponds to approximately twice the GC dendrite length since this is the largest distance over which two GCs would be likely to sample the same input. To test whether this was indeed the case we computed the GC covariance as a function of network radius for a binary variant of the anatomically constrained local GCL network model. The covariance matrix was determined for a network having 4 synaptic connections per binary GC, threshold of 2 and $p(MF) = 0.5$ for 4000 events. Values in the covariance

matrix were plotted as a function of the corresponding pairwise distance between GCs (Figure S2C). Covariance between cells was zero within a radius of $40\mu m$, confirming our prediction from anatomical considerations. While variation in the parameters used changed the magnitude of covariance, it did not alter its spatial profile.

2.5 Relative fixed threshold and network activity-dependent granule cell threshold function in uniform binary network models

We implemented a relative fixed threshold in the uniform binary network, which was analogous to keeping a fixed tonic inhibition in real cells but scaling down the size of the excitatory synaptic conductances when increasing the number of MF synaptic connections per cell. In practice, this was implemented by setting the GC threshold to 75% of the inputs or the nearest integer value above this using ceiling[$0.75 \cdot d$]. Furthermore, to explore how information processing in uniform binary networks was affected by a network activity-dependent granule cell threshold function as is likely to be mediated by the feedforward inhibitory Golgi circuit (Kanichay and silver, 2008; Sawtell, 2010), we introduced a dependence between the threshold of the GCs and the probability of MF activation ($p(MF)$). To do this we set the GC threshold to progressively higher levels with $p(MF)$ using a piecewise constant function. The 'steepness' of this relationship is controlled by a parameter setting the network activity-dependent threshold, denoted 'NADT'. The NADT was defined as $1/(d \times \text{interval length})$ where the interval length is the width of the constant $p(MF)$ regions of the threshold function and d is the number of synaptic connections per GC. Higher NADT implied a smaller interval length and a correspondingly steeper change of the GC threshold with MF activation, thus mimicking greater inhibition.

2.6 Calculation of Shannon information in uniform binary networks

For binary networks we determined the Shannon information of the GC output and compared it to the raw event information encoded in the MFs inputs to assess the information transmission efficiency through the network. Since signal transmission in these networks is noise free, the Shannon information is equal to the raw output entropy. We defined the encodable information as that arising from the set of sensory-motor states that the animal could encounter during its lifetime. To do this we assumed N events occur in the lifetime of the animal, with one unique event occurring in each small time interval, which was taken as $30ms$, implying a maximum of 1 billion events in the 1 year lifespan of a small rodent in the wild. The binary MF code representing these events was defined as the randomly realized state (0 or 1) of the MF inputs to the network. Unfortunately the direct method for calculating information (Eq.(1)) is excessively computationally demanding when considering such a large number of distinct MF input patterns and the need to average across many instances of an individual network, given the random nature of their synaptic connectivity. Moreover, this was compounded by the fact that we wanted to systematically investigate networks with different numbers of synaptic connections and different MF activation probabilities. We therefore developed an alternative, analytical method for rapidly calculating information in uniform binary networks. The maximum information carried by the GC output is equal to the entropy of the MFs (H_{MF}), but the entropy realized by the GCs H_{GC} depends on the number of synaptic connections per GC (d), their thresholding ϕ and their numerosity γ. Our approach utilizes Eq. 29, given with its derivation in the Appendix (below), to calculate H_{GC}.

2.6.1 Identification of the sources of error in estimating entropy in uniform binary network models

Since information transmission can be computed by explicit enumeration of input and output codes for a modest number of patterns, we used this approach

to test the validity of our analytical approach for calculating H_{GC} and for examining the effect of reducing the anatomically constrained local network structure to a uniform bipartite network. Connections in the anatomically constrained local network were described by a connection matrix **A**, with elements $a_{ij} = 1$ if MF i connects to GC j and $a_{ij} = 0$ otherwise. In the anatomically constrained local network this matrix was determined by the 3D spatial pattern of connectivity between GCs and glomeruli which was generated as described above, where as in the case of the uniform binary model this was simply a random matrix with the only constraints that GCs could connect each MF only once and each GC connected to exactly d MFs. To simulate network activity, a random binary vector **x** representing an MF activation pattern was multiplied by the connection matrix. Each element of the resulting vector was then thresholded by the GC threshold level to yield the binary GC outputs $\mathbf{y} = \Phi(\mathbf{Ax})$. The mapping from MF patterns to GC patterns ($\mathbf{x} \rightarrow \mathbf{y}$) was therefore random, by virtue of the random network connections, but note that in the event of a repeat input **x** the identical GC pattern **y** would reoccur; this was justified by the assumption that the noise arising from synaptic transmission and firing was negligible over each 30 ms integration window.

Generation of output patterns from input patterns in this manner enables the entropy of the output patterns to be determined. For N events, M unique input patterns may be produced ($M = N$ only if each MF realization is unique for every event), which then map to G unique GC patterns (in general, $G \leq M$ since some of the M MF patterns could map to duplicate GC patterns). The mutual information between these G GC vectors and the N events for a particular network can be characterized by the empirical entropy (Cover & Thomas, 1991; Shannon, 1948) of the GC patterns since our network is noise free,

$$H_{GC} = -\sum_{k=1}^{G} p(\mathbf{y}_k) \log_2[p(\mathbf{y}_k)] \tag{1}$$

where $\mathbf{y}_k$ is a binary GC state vector and $p(\mathbf{y}_k)$ is the empirical probability of that GC state given a particular realization of the random network connections; $p(\mathbf{y}_k) =$

Q_k/N where Q_k is the number of times that GC output vector k occurs over the N events. Note that no undersampling bias correction (Treves and Panzeri, 1995) is required, since given a single network and a single set of MF patterns, the mapping from MFs $\rightarrow$ GCs is deterministic.

The empirical entropy of the MFs (H_{MF}) is determined by the probability of activation of an individual input $p(MF)$, the number of inputs μ and the number of events. The information rate of MF pattern generation is the standard quantity

$$-\mu[p(MF)\log_2(p(MF)) + (1-p(MF))\log_2(1-p(MF))] \quad (2)$$

and is the value that the empirical entropy converges to for an infinite number of events. The maximum information rate of MF pattern generation (occurring when $p(MF) = 0.5$) is the input capacity of the network $C = \mu$ bits and is the upper limit of the event information that *could* be represented by the binary MFs.

To examine error in the estimation of GC entropy using our analytical approach (Eq. (29); Appendix) we compared the GC entropy in binary networks with a directly simulated uniform binary network for a smaller number of MF input patterns, using the direct method. For 4000 MF patterns we are able to estimate GC entropy over the full range of parameter space (Figure S3). The discrepancy between the analytical calculation method and the direct calculation method never exceeded a fraction of a bit for the region of parameter space we explored (Figure S3A,D). However a larger discrepancy was observed between the uniform bipartite graph networks and the anatomically constrained network model, both calculated with the direct method (Figure S3B,E) or with the analytical and direct method, respectively ((Figure S3C,F). Thus the error arising from the the differences in connectivity between the anatomically constrained network model and the bipartite graph is larger than that introduced by our rapid analytical method for calculating entropy in bipartite networks. The maximum error introduced by simplifying network structure is less than 1 bit for networks up to around 10 connections but is more pronounced for networks having many connections (Figure S3B,E).

2.7 Biologically detailed spiking network model

2.7.1 Mossy fiber spike train generation

The anatomically constrained local GCL network structure was used to construct the biologically detailed spiking network models. For each simulation configuration, a variable fraction $p(MF)$ of the 176 total MF synaptic inputs were randomly selected and designated as active. When simulating spatially correlated inputs, MF terminals were activated in groups of 5, starting by selecting one terminal at random and activating it together with its 4 closest neighbors. For each repetition of the simulation configuration, active MF input spike trains were then generated as instances of the Poisson stochastic process with a mean rate of 80Hz, except for simulations in Figure S5 where other rates were explored. For the remaining inactive MFs, Poisson spike trains of 10 Hz rate were generated. The NEURON code generated by neuroConstruct used NEURON's built in NetStim and mcell_ran4 for pseudorandom event generation.

2.7.2 Synaptic conductance models

The active and inactive MF spike trains were then used to build trains of synaptic conductances for each MF input (i.e.rosette). Each individual excitatory synaptic conductance occurred at the time of a MF spike and consisted of both AMPAR- and NMDAR-mediated conductance components (G_{AMPA} and G_{NMDA}, respectively). These synaptic conductances exhibited distinct waveforms and voltage and time dependencies, as well as short term plasticity as for real MF-GC inputs. Values for all the parameters of the synaptic models, together with their provenance (or the source of the relevant experimental data) are reported in Table S3.

AMPAR-mediated synaptic waveform. The total AMPAR conductance component (i.e from direct quantal release and glutamate spillover) arising from a

single MF spike is given by

$$G_{AMPA} = p_{AMPA,d} \cdot g_{AMPA,d} + p_{AMPA,s} \cdot g_{AMPA,s}$$

where

$$g_{AMPA,d} = \sum_{i=1}^{2} a_i^{(AMPA,d)} (e^{-t/\rho^{(AMPA,d)}} - e^{-t/\delta_i^{(AMPA,d)}})$$
$$g_{AMPA,s} = \sum_{i=1}^{3} a_i^{(AMPA,s)} (e^{-t/\rho^{(AMPA,s)}} - e^{-t/\delta_i^{(AMPA,s)}})$$

and $a_i^{(AMPA,d)}$, $\rho^{(AMPAd)}$, $\delta_i^{(AMPA,d)}$ and $a_i^{(AMPA,s)}$,$\rho^{(AMPA,s)}$,$\delta_i^{(AMPA,s)}$ indicate component amplitudes, rise times and decay times for the direct and glutamate spillover-only components of the AMPAR synaptic waveform, respectively. $p_{AMPA,d}$ and $p_{AMPA,s}$ are the independent short-term depression scaling factors for the direct and spillover components, respectively, and are both defined as per the standard NeuroML2 *TsodyksMarkramDepMechanism* component type, as described below.

NMDAR-mediated synaptic waveform. The total unblocked NMDAR conductance component arising from a single MF spike is given by

$$G_{NMDA} = p_{NMDA} \cdot \sum_{i=1}^{2} a_i^{(NMDA)} (e^{-t/\rho^{(NMDA)}} - e^{-t/\delta_i^{(NMDA)}})$$

where $a_i^{(NMDA)}$, $\rho^{(NMDA)}$ and $\delta_i^{(NMDA)}$ indicate conductance amplitudes, rise time and decay times for the two components of the waveform. The p_{NMDA} scaling factor implements short term depression *and* facilitation of the NMDAR component and is defined as per the standard NeuroML2 *TsodyksMarkramDepFacMechanism* component type, as described below.

Mg^{2+} block mechanism. A Mg^{2+} block mechanism for the NMDAR-mediated synaptic conductance component was modeled as a custom LEMS component type by defining an unblock function $b(V)$ with the Woodhull formalism (Rothman & Silver, 2014):

$$b(V) = \frac{C_1 e^{\delta_{bind}\theta V} + C_2 e^{-\delta_{perm}\theta V}}{C_1 e^{\delta_{bind}\theta V} + C_2 e^{-\delta_{perm}\theta V} + [Mg^{2+}]_{out} e^{-\delta_{bind}\theta V}}$$

where V is the postsynaptic membrane potential, and $\theta = zF/RT$, with z as the Mg^{2+} ionic charge, F the Faraday constant, R the ideal gas constant, and T the absolute temperature.

Synaptic plasticity mechanisms. The short term plasticity models in the simulations make use of NeuroML2's standard *TsodyksMarkramDepMechanism*[1] and *TsodyksMarkramDepFacMechanism*[2] component types. These, in turn, are defined as the models formulated in, respectively, Tsodyks & Markram (1997) and Tsodyks et al. (1998), simplified to ignore inactivation. For a synaptic conductance g, a plasticity mechanism defines a factor p which multiplies the contribution of a single synaptic event before it gets added to g.

As defined in the NeuroML2 documentation, *TsodyksMarkramDepFacMechanism* is parametrized by an *initial release probability* r, a *depression recovery time* Δ and a *potentiation recovery time* Π. p is defined as

$$p(t) = U(t) \cdot R(t)$$

where $U(t)$ and $R(t)$ are the internal dynamical variables of the model, which are initialized as

[1] *http://www.neuroml.org/NeuroML2CoreTypes/Synapses.html#tsodyksMarkramDepMechanism*
[2] *http://www.neuroml.org/NeuroML2CoreTypes/Synapses.html#tsodyksMarkramDepFacMechanism*

$$U(0) = r$$
$$R(0) = 1$$

Following a synaptic event, U and R get updated according to

$$U \to U + r \cdot (1 - U)$$
$$R \to R \cdot (1 - U)$$

while, in absence of synaptic events, they decay exponentially to their initial values:

$$\frac{dU}{dt} = -\frac{U - r}{\Pi}$$
$$\frac{dR}{dt} = -\frac{R - 1}{\Delta}$$

Finally, *TsodyksMarkramDepMechanism* is a simpler version of *TsodyksMarkramDepFacMechanism* where U is fixed to its initial value.

Derivation of synaptic model parameters from experimental data. Estimates for the parameters for the synaptic waveform shape and plasticity mechanisms were recomputed from the experimental data published in Rothman et al. (2009) with a particle swarm optimization algorithm (Deb & Padhye, 2010) implemented using the inspyred framework (Garrett, 2014). Parameters for the Mg^{2+} block mechanism were set to the values published in Schwartz et al. (2012).

2.7.3 Granule cell model

Granule cells were modeled as standard refractory conductance-based integrate-and-fire neurons as per the *IaFRefCell* component type definition[3] in NeuroML2. For these model GCs the membrane voltage evolves according to

$$
\begin{aligned}
-C_m\frac{dV}{dt} &= G_m\cdot(V-E_m)+G_{GABAR}\cdot(V-E_{GABAR})+\\
&+\sum_{i=1}^{d}G_{AMPAR}(i;t)\cdot(V-E_{AMPAR})+b(V)\cdot\sum_{i=1}^{d}G_{NMDAR}(i;t)\cdot(V-E_{NMDAR})
\end{aligned}
\tag{3}
$$

where C_m is the membrane conductance, E_m the reversal potential of the membrane leak conductance, G_{GABAR} and E_{GABAR} the tonic GABAR-mediated conductance and reversal potential, $G_{AMPAR}(i;t)$ and $G_{NMDAR}(i;t)$ the AMPAR and NMDAR-mediated conductance trains computed from the MF input spike trains, E_{AMPAR} and E_{NMDAR} the AMPAR and NMDAR reversal potentials, and $b(V)$ the NMDAR Mg^{2+}unblock function defined above. Upon V reaching the threshold value V_t a spike is emitted, and V is then clamped to the reset potential V_r for a refractory interval τ_r. Values and experimental provenance for all parameters in the model are reported in Table S3.

2.7.4 Simulation management

Single cell and synaptic models were serialized as LEMS/NeuroML2 files. Instantiations of the anatomically detailed network model were generated with Mathematica (Wolfram Research, Champaign, USA-IL) and exported in the GraphML format (Brandes et al., 2002). These were loaded, respectively, through neuroConstruct's Jython scripting interface (Gleeson et al., 2007) and networkX (Hagberg et al., 2008) into custom Python software that generated NEURON simulations (Carnevale and Hines, 2006) through neuroConstruct, distributing the computational load from code generation and simulation across two HPC platforms (the SilverLab's own cluster and UCL's Legion cluster) using the Sun Grid

[3]http://www.neuroml.org/NeuroML2CoreTypes/Cells.html#iafRefCell

Engine job queuing system. Spike time data was stored in compressed hdf5 archives using h5py (Collette, 2013).

2.8 Analysis of spike trains

Mutual information (MI) was calculated between the set of N MF input patterns and N output network activity classes obtained by performing a Voronoi (nearest-neighbor) tessellation of the output space. N was the smallest number of output classes that allowed full recovery of information, given that we assumed a uniform prior over the inputs. The N seed points for the tessellation were the centroids of the clusters obtained by running the k-means algorithm (Lloyd, 1982) as implemented in scikit-learn (Pedregosa et al., 2011) on a training dataset of 30 repetitions per pattern. To avoid being trapped in local minima, the algorithm was re-initialized 10 times using k_means++ (Arthur & Vassilvitskii, 2007), and the best clustering solution was used in the computation of the centroids. Training data was not re-used for the computation of MI. Information, which had an upper bound equal to the input entropy ($\log_2 1024 = 10$ bits for 1024 patterns), was calculated with the pyentropy package (Ince et al., 2009). Undersampling bias in the MI estimate (Panzeri et al., 2007) was accounted for with the *quadratic extrapolation* procedure (Strong et al., 1998) (Figure S4D, bottom). Other bias correction methods (Nemenman et al., 2002; Panzeri & Treves, 1996) were considered, but did not to provide a significantly better performance (data not shown). Population sparseness was computed using the definition in Vinje and Gallant (2000) (see main text). An initial 150ms transient was discarded from all simulations to allow the system to go from resting to steady state for the pattern being simulated. Independent recordings of the response to a pattern were extracted from a single simulation by slicing it in 30ms-long time frames and discarding every other frame, to allow the system state to decorrelate between the frames we kept for analysis. This is justified by the characteristic time of the autocorrelation function of the GC spiking output being of the order of 30ms (Figure S4B).

Table S3, related to Figure 7 and Supplemental Experimental Procedures: Parameter values used in the biologically constrained spiking model, and sources for the relevant experimental information.

Parameter	Value	Source	Parameter	Value	Source
E_m	-79.9 mV	Schwartz et al. (2012)	E_{NMDAR}	0 mV	Rothman et al. (2009)
G_m	1.06 nS		$a_1^{(NMDA)}$	17 nS	Schwartz et al. (2012)
C_m	3.22 pF		$a_2^{(NMDA)}$	2.645 nS	
V_t	-40 mV		$\rho^{(NMDA)}$	0.8647 ms	Rothman et al. (2009)
V_r	-63 mV		$\delta_1^{(NMDA)}$	13.52 ms	
τ_r	2 ms		$\delta_2^{(NMDA)}$	121.9 ms	
E_{GABAR}	-79.1 mV	Seja et al. (2012)	$r^{(NMDA)}$	0.0322	
G_{GABAR}	0.438 nS	Rothman et al. (2009)	$\Delta^{(NMDA)}$	236.1 ms	
E_{AMPAR}	0 mV		$\Pi^{(NMDA)}$	6.394 ms	
$a_1^{(AMPA,d)}$	3.724 nS		z	2	Schwartz et al. (2012)
$a_2^{(AMPA,d)}$	0.3033 nS		T	308.15K	
$\rho^{(AMPA,d)}$	0.3274 ms		$[Mg^{2+}]_{out}$	1 mM	
$\delta_1^{(AMPA,d)}$	0.3351 ms		δ_{bind}	0.35	
$\delta_2^{(AMPA,d)}$	1.651 ms		δ_{perm}	0.53	
$r^{(AMPA,d)}$	0.1249		C_1	2.07 mM	
$\Delta^{(AMPA,d)}$	131 ms		C_2	0.015 mM	
$a_1^{(AMPA,s)}$	0.2487 nS				
$a_2^{(AMPA,s)}$	0.2799 nS				
$a_3^{(AMPA,s)}$	0.1268 nS				
$\rho^{(AMPA,s)}$	0.5548 ms				
$\delta_1^{(AMPA,s)}$	0.4 ms				
$\delta_2^{(AMPA,s)}$	4.899 ms				
$\delta_3^{(AMPA,s)}$	43.1 ms				
$r^{(AMPA,s)}$	0.2792				
$\Delta^{(AMPA,s)}$	14.85 ms				

Note: because of how the STP mechanisms are defined, the maximum amplitude of a conductance pulse isolated in time will be of the order of $r^{(AMPA)} \cdot a^{(AMPA)}$ for AMPA and $b(V) \cdot r^{(NMDA)} \cdot a^{(NMDA)}$ for NMDA. This, in practice, means a maximum peak amplitude of 630pS for both AMPA (Sargent et al., 2005), and unblocked NMDA (Schwartz et al., 2012).

3 Appendix

3.1 Analytical method to compute granule cell empirical entropy in the Uniform Binary Network

The connectivity of the GC layer can be simplified to a random bipartite graph with two sets of nodes, one for the MF rosettes (inputs) and one for the GCs (outputs). Each output node is randomly connected to d inputs, corresponding to each GC having d synaptic connections. If an output node is connected with an input node, the graph is said to have an *edge* between the two. We modeled GCs as linear threshold (binary) units, active if the number of active binary synaptic inputs is greater than or equal to some value ϕ. In this appendix we refer to this spatially uniform binary network model as a UBN (in correspondence to the main text). Our aim in studying the UBN is to understand the properties of random feedforward network encoders.

Statement of mathematical problem: Encoding is performed by the UBN by mapping from N unique samples to M unique patterns on the input layer of the UBN (MFs), which are in turn mapped onto G unique patterns on the output layer (GCs). These mappings are deterministic, but the patterns arising on the input are randomly determined. Parameters of the network model determine both M and G. Since all mappings are deterministic, information is lost whenever the mapping from events to output patterns is not a bijection. We calculated the entropy of the G output patterns to quantify the conservation of the information of the N encoded samples. Hence we quantified how the network parameters impact the conservation of information. For notation, see Tables S4 and S5.

3.1.1 Mossy fiber inputs

Input patterns and symbols: MF synaptic rosettes are represented by the input layer of the UBN model. We denote the number of such input nodes as μ. Each input is either on or off and input patterns to the network are μ element binary vectors $\hat{\chi}$:$\{\chi_\lambda \in \{0,1\}\}$ for all $\lambda \in \{0,...,\mu-1\}$. Let X denote the set of all such vectors. Define $\Omega^{(X)}$ as the index set of N vectors chosen from X, containing the integers associated with each binary input vector in the following manner: for the lth vector $\hat{\chi}^{(l)} \rightarrow \omega_l^{(X)} = \sum_{\lambda=0}^{\mu-1} 2^\lambda \chi_\lambda^{(l)}$ where $l \in \{1,...,N\}$ and $\omega_l^{(X)} \in \{0,...,2^{\mu-1}\}$ is a single integer in the index set. Each one of the integers $\omega_l^{(X)}$ is referred to as a *symbol* in order to distinguish it from the *pattern* $\hat{\chi}^{(l)}$ associated with it (which is a vector). $\Omega^{(X)}$ is referred to as the input alphabet. Since there is a one-to-one mapping between patterns and symbols , they are identical from the point of view of Shannon information, Eq. (1). However, patterns can have properties, such as the number of active inputs while symbols should be viewed here as an accounting device to keep track of the number and probability of distinguishable patterns.

Input information and the alphabet: The pattern of activity from the sensory receptors of an animal is unlikely to ever repeat precisely due to the huge numbers of transducers involved in raw sensation, but repeated body movements in familiar environments could result in very similar MF activation patterns. Downstream networks may learn from MF states by clustering them (facilitated by expansion and sparsening in the GCL) thereby flexibly identifying occurrences of similar states. Maximum flexibility is afforded if the MF patterns retain as much of the raw sensory event information as possible prior to downstream learning. To reflect this picture of sensory encoding we consider random activation patterns occurring in MF afferents as a result of sensory motor states during short intervals of time, which we call events. In the biological context the precise input pattern that results from each event is determined by random factors that differ in detail from animal to animal: developmental, morphological and physiological factors such as the microscopic arrangement of sensory afferents carrying impulses from

each somatic area and the precise activity signature from sensory cells. Hence in our model each event (microscopic sensory state) is thought of as mapping to a pattern of activity (MF input vector) that is determined by a single random realization of the MF inputs, assuming that for this realization the binary input variables are independent and identically distributed Bernoulli trials $\hat{\chi} : p(\chi_\lambda = 1) = p_{MF}$ for all $\lambda \in \{0, .., \mu - 1\}$ (p_{MF} denoted $p(MF)$ in the main text). We make the simplifying assumption that the event $\rightarrow$ pattern transduction has no noise, such that the events map *deterministically* to the random MF patterns, i.e. although the MF patterns are themselves random, their mapping to sensory events is constant. We assume that the number of possible events is sufficiently large that in practice the events occurring within the lifetime of the organism are all unique. There are many ways in which N MF patterns can denote N events. We describe each one of these mappings from the events, $1, 2, ..., N$ to the integers in the input alphabet $\Omega^{(X)}$, using a mapping $m = \{1 \rightarrow \omega_1^{(X)}, 2 \rightarrow \omega_2^{(X)}, ..., N \rightarrow \omega_N^{(X)}\}$, where the ordering of events is not considered important. The set of all such mappings $\mathcal{M} = \{m_1, m_2, ..., m_Z\}$ corresponds to the set of all possible input alphabets for the sequence of N events. Each alphabet can be thought of as representing the way that the nervous system of an individual animal responds to events.

The empirical probability of occurrence of an input symbol given some alphabet $p(\omega^{(X)}|m)$: When sampling from a probability distribution, we call the *empirical probability* the fraction of the total outcomes accounted for by each unique outcome (i.e. the normalized frequency of outcomes). Applied to input symbols, the empirical probability only assumes knowledge of the input alphabet, requiring that we make minimal assumptions about the process generating the events themselves. The empirical input symbol probability is

$$p(\omega^{(X)}|m) = \frac{1}{N} \sum_{l=1}^{N} I(\omega_l^{(X)} = \omega^{(X)}, m) \tag{4}$$

where $I \in \{0, 1\}$ is an indicator function that adopts the value 1 if and only if code $\omega_l^{(X)}$ is equal to $\omega^{(X)}$ for $\omega_l^{(X)} \in m$. As the number of events tends to infinity ($N \rightarrow \infty$), the empirical probabilities tend to their true probabilities ($p(\omega^{(X)}|m) \rightarrow p(\hat{\chi})$),

whereupon the empirical symbol probability becomes independent of the alphabet.

Computation of the empirical probability of a symbol $p(\omega^{(X)}, s_X)$: The input success class c_{s_X} is the set of those symbols whose patterns have exactly s_X active inputs. Since the underlying probability measure of the MF patterns is that of μ independent identically distributed binary variables, the number of times a symbol is realized depends only on the success class s_X. Let $u_n^{s_X}$ denote the mean number of unique draws of a symbol from success class c_{s_X} after n samples from that class, where $l_{n-1}(\omega \in c_{s_X})$ is the probability that the nth code taken from success class s_X is unique (i.e. has not yet been sampled) after $n-1$ draws. Symbols are drawn randomly with equal probability, hence $l_{n-1}(\omega \in c_{s_X}) = \left[\binom{\mu}{s_X} - u_{n-1}^{s_X}\right] / \binom{\mu}{s_X}$,

$$
\begin{aligned}
u_n^{s_X} &= u_{n-1}^{s_X} + l_{n-1}(\omega \in c_{s_X}) \\
&= 1 + u_{n-1}^{s_X} \left(\frac{\binom{\mu}{s_X} - 1}{\binom{\mu}{s_X}} \right)
\end{aligned}
\tag{5}
$$

Letting $r_{s_X} = \left[\binom{\mu}{s_X} - 1\right] / \binom{\mu}{s_X}$ we can find a homogeneous recurrence relation that is solved using standard methods,

$$
\begin{aligned}
u_{n+1}^{s_X} &= 1 + u_n r_{s_X} \\
u_{n+1}^{s_X} - u_n^{s_X} &= 1 + u_n^{s_X} r_{s_X} - (1 + u_{n-1}^{s_X} r_{s_X}) \\
&= u_n^{s_X} r_{s_X} - u_{n-1}^{s_X} r_{s_X} \\
u_{n+1}^{s_X} &= u_n^{s_X}(1 + r_{s_X}) - u_{n-1}^{s_X} r_{s_X}
\end{aligned}
\tag{6}
$$

having characteristic equation,

$$
r_{s_X}^2 = (1 + r_{s_X}) r_{s_X} - r_{s_X} \tag{7}
$$

where we used the ansatz $u_n^{s_X} = r_{s_X}^n$ and divided through by $r_{s_X}^{n-1}$. Eq.(7) has

roots,

$$(1/2)\left(1 \pm (r_{s_X} - 1) + r_{s_X}\right) \tag{8}$$

the general solution to the recursion relation is obtained by summing the roots with indefinite weighting,

$$u_n^{s_X} = A + Br^n. \tag{9}$$

Specific solutions can now be obtained by determining coefficients in Eq.(9): solving for $u_0^{s_X} = 0$, we find $B = -A$ and when $u_1^{s_X} = 1$ (since the first draw must be unique), $A = -1/(r_{s_X} - 1)$, $B = 1/(r_{s_X} - 1)$,

$$u_n^{s_X} = \frac{(r_{s_X}^n - 1)}{(r_{s_X} - 1)}. \tag{10}$$

Hence the empirical probability of any single code in success class s_X within a finite sample of N events is $p(\omega^{(s_X)}) = p(s_X)/u_{Np(s_X)}^{s_X} = p(s_X)[(r_{s_X} - 1)/(r_{s_X}^{Np(s_X)} - 1)]$.

3.1.2 Granule cell output

Granule cells: GCs are represented by the output nodes of the network. We denote the number of such output nodes as γ. Each output node is either on or off ($\nu \in \{0, 1\}$) which is determined by the thresholded summed inputs,

$$\alpha = \sum_{i=1}^{\mu} I_i \chi_i \tag{11}$$

$$\nu = \begin{cases} 1 & \text{if } \alpha \geq \phi \\ 0 & \text{if } \alpha < \phi \end{cases} \tag{12}$$

where $I = 1$ if the granule cell is connected to MF input i, $I = 0$ otherwise and ϕ is the minimum number of inputs that must be active for the output to switch from off (0) to on (1). The vector $\hat{\nu}(\hat{\chi})$:$\{\nu_\kappa \in \{0, 1\}\}$ for all $\kappa \in \{0, .., \gamma - 1\}$ thus defines an output pattern for each input pattern $\hat{\chi}$ in X and we denote the set of all such output patterns Y.

Output patterns and symbols: We define the index set of the output patterns as $\Omega^{(Y)}$, containing symbols which are the integers $\omega_l^{(Y)} \in \{0, ..., 2^{\gamma-1}\}$ for all $l \in \{1, ..., N\}$ derived from output patterns using the identical method used to map the input patterns to integer symbols. The network therefore performs a mapping of a set of input symbols on to a set of output symbols $f : \omega_l^{(X)} \to \omega_l^{(Y)}$ for all l. This mapping determines the empirical output symbol probability (and hence how much information is captured) and the relative properties of the input and output patterns such as their activity levels. Our model contains no transmission noise so each output pattern occurs deterministically in response to an input pattern (which is itself deterministically mapped to an event by the event mapping). However, the random connections between inputs and outputs in combination with the variable output thresholding ϕ mean that in general not all input patterns are mapped to unique output patterns, permitting the empirical entropy of the output symbols to differ from the entropy of the input symbols in a manner that depends on network parameters. For a network to encode all of the event information, i) the activity level of the inputs must be within a range that allows their variation to fully encode the events and ii) the parameters of the network should ensure a one to one mapping from input symbols to output symbols.

Output information: For a given set of input symbols and a given network realization a measure of the information of the output symbols can be determined directly from the empirical entropy of the output symbols conditional on the network (Cover & Thomas, 1991),

$$H(\Omega^{(Y)}|\theta, m) = -\sum_{\omega^{(Y)} \in \Omega^{(Y)}} p(\omega^{(Y)}|\theta, m) \log_2[p(\omega^{(Y)}|\theta, m)] \qquad (13)$$

where the empirical entropy of the output alphabet is conditional on the network realization θ and event input alphabet m. Even given an identical set of input symbols having identical probabilities of occurrence, Eq. (13) will evaluate differently for different networks. The conditional entropy itself is therefore a random variable. For this reason we seek the mean conditional entropy over network realizations and event mappings

$$\left\langle H(\Omega^{(Y)}|\theta, m) \right\rangle_{\Theta,\mathcal{M}} = \left\langle - \sum_{\omega^{(Y)} \in \Omega^{(Y)}} p(\omega^{(Y)}|\theta, m) \log_2[p(\omega^{(Y)}|\theta, m)] \right\rangle_{\Theta,\mathcal{M}}$$
$$= - \sum_{\omega^{(Y)} \in \Omega^{(Y)}} \left\langle p(\omega^{(Y)}|\theta, m) \log_2[p(\omega^{(Y)}|\theta, m)] \right\rangle_{\Theta,\mathcal{M}}$$

We calculate this average in the "annealed" approximation, i.e. substituting

$$\left\langle p(\omega^{(Y)}|\theta, m) \log_2[p(\omega^{(Y)}|\theta, m)] \right\rangle \simeq \left\langle p(\omega^{(Y)}|\theta, m) \right\rangle \log_2 \left[\left\langle p(\omega^{(Y)}|\theta, m)] \right\rangle \right]$$

We have checked the validity of this approximation numerically and by examining the Taylor expansion of Eq. (13); deviations from the annealing assumption are small if $p(\omega^{(Y)}|\theta, m) \in [0, 1]$, which is always true as p is a probability.

Approximation of the granule cell output entropy : We require the expectation of Eq. (13) with respect to the network realization and input symbols. Let Θ denote the index set of all possible networks existing between the outputs and the inputs. Each member θ of this set gives rise to a different mapping of input symbols onto output symbols. In analogy to the symbols, θ can be thought of as the integers associated with each binary vector indexing a given network realization. This vector consists of $\mu\gamma$ dimensions (it is an 'unrolled' connection matrix), with each value being equal to one if there is an edge between the corresponding input/output pair and zero otherwise. Given a single realization of the network and event mapping we can determine with certainty whether for that realization, some input symbol $\omega^{(X)}$ maps to some output symbol $\omega^{(Y)}$, since this mapping is deterministic. Hence we can define an indicator function on the sets $\Omega^{(Y)}$, $\Omega^{(X)}$, Θ and $\mathcal{M}$, which takes the value 1 if and only if output code $\omega^{(X)}$ occurs as a result of alphabet m, and gives rise to $\omega^{(Y)}$ for network

configuration θ. Let $I(\omega^{(Y)}, \omega^{(X)}, \theta, m)$ denote this function. Given a single network realization θ the probability of an output symbol must be the sum of the probabilities of the input symbols giving rise to it since the outputs are deterministic, $p(\omega^{(Y)}|\theta, m) = \sum_{\omega^{(X)} \in c_{\omega^{(Y)}}} p(\omega^{(X)}|m) = \sum_{\omega^{(X)}=0}^{2^\mu - 1} p(\omega^{(X)}|m) I(\omega^{(Y)}, \omega^{(X)}, \theta, m)$, where $c_{\omega^{(Y)}}$ is the set of all input symbols that give rise to output symbol $\omega^{(Y)}$ for network θ and event mapping m. In Eq.(13) the logarithm is defined only for $\omega^{(Y)}$ which occur. Therefore we determine the mean empirical symbol probability in the case where $\omega^{(Y)}$ always exists in the output alphabet given the network realization θ and alphabet m. We develop this quantity further in due course, but for now we denote it $\{p(\omega^{(Y)}|\theta, m)\}_{\Theta, \mathcal{M}}$ where the curly braces indicate that this is an average over Θ and $\mathcal{M}$ under the probability measure in which $\omega^{(Y)}$ must exist. The expected conditional entropy is therefore,

$$\begin{aligned} H(\Omega^{(Y)}|\Theta, \mathcal{M}) \;=\; & -\sum_{\omega^{(Y)}=0}^{2^\gamma - 1} \sum_{\omega^{(X)}=0}^{2^\mu - 1} \left\langle p(\omega^{(X)}|m) I(\omega^{(Y)}, \omega^{(X)}, \theta, m) \right\rangle_{\Theta, \mathcal{M}} \cdot \\ & \cdot \log_2 [\left\{ \sum_{\omega^{(X)}=0}^{2^\mu - 1} p(\omega^{(X)}|m) I(\omega^{(Y)}, \omega^{(X)}, \theta, m) \right\}_{\Theta, \mathcal{M}}]. \end{aligned} \quad (14)$$

The remainder of the appendix is concerned with determining the components of Eq. (14) in terms of the macroscopic properties of the network only, such as the number of inputs, the number of outputs and the activity level of the inputs.

Determination of $\langle p(\omega^{(X)}|m) I(\omega^{(Y)}, \omega^{(X)}, \theta, \mathcal{M}_j) \rangle_{\Theta, \mathcal{M}}$ in Eq. (14): We first make the following observations: i) In the uniform binary model, the outputs are conditionally independent given the input $\omega^{(X)}$ since they are not themselves directly connected by any graph edges, hence

$$\sum_{\theta=0}^{2^{\mu\gamma} - 1} p(\theta) \prod_{j=1}^{\gamma} I(\nu_j = g_j, \omega^{(X)}, \theta, m) = p(\underline{\nu} = \underline{g}, \omega^{(X)}, m) = \prod_{j=1}^{\gamma} p(\nu_j = g_j | \omega^{(X)}, m) \quad (15)$$

ii) The input alphabet makes no difference to the probability of an output being active given some input symbol, $p(\nu_1 = g_1 | \omega^{(X)}, m) = p(\nu_1 = g_1 | \omega^{(X)})$ because only

the empirical probability of $\omega^{(X)}$ is affected by the choice of alphabet, not the state of a single output given that $\omega^{(X)}$ has occurred. Applying these observations,

$$\sum_{m\in\mathcal{M}} p(m)p(\omega^{(X)}|m) \sum_{\theta=0}^{2^{\mu\gamma}-1} p(\theta) I(\omega^{(Y)}, \omega^{(X)}, \theta, m) =$$

$$= \sum_{m\in\mathcal{M}} p(m)p(\omega^{(X)}|m) \sum_{\theta=0}^{2^{\mu\gamma}-1} p(\theta) \prod_{j=1}^{\gamma} I(\nu_j = g_j, \omega^{(X)}, \theta, m) \quad \text{[applying (i) above]}$$

$$= \sum_{j=1}^{Z} p(m)p(\omega^{(X)}|m) \prod_{j=1}^{\gamma} p(\nu_j = g_j|\omega^{(X)}, m) \quad \text{[applying (ii) above]}$$

$$= p(\omega^{(X)})p(\nu_1 = g_1|\omega^{(X)})...p(\nu_\gamma = g_\gamma|\omega^{(X)}) \quad (16)$$

where to perform the desired computation of Eq. (16) we must determine the conditional output activation probability $p(\nu = 1|\omega^{(X)})$ for a single output given presentation of an input symbol.

Determination of the conditional output activation probability $p(\nu = 1|\omega^{(X)})$ in Eq. (16): Every input symbol $\omega^{(X)}$ can be placed into a class c_{s_X} having some number s_X of active inputs. Consider the state of the UBN when a single input pattern from c_{s_X} is applied to the inputs. Each output is connected at random to the inputs and therefore samples both active and inactive inputs. Since every output makes d connections to the inputs, there can be no more than max(d, s_X) active inputs connected to each output and we wish to know how many active inputs are sampled. This can be seen as a sampling problem, where given a population of μ variables in which s_X are active, we wish to know how many active variables we sample after taking d samples without replacement; this is precisely the sampling described by the hypergeometric distribution. Therefore, the probability that each output is activated given s_X active inputs is the mass of the hypergeometric distribution between the threshold ϕ and the maximal active units max(d, s_X),

$$p(\nu = 1|\omega^{(s_X)}) = \sum_{z=\phi}^{\max(d,s_X)} \frac{\begin{pmatrix} s_X \\ z \end{pmatrix} \begin{pmatrix} \mu - s_X \\ d - z \end{pmatrix}}{\begin{pmatrix} \mu \\ d \end{pmatrix}} \tag{17}$$

where the notation $p(\nu = 1|\omega^{(s_X)})$ indicates the probability of activation of an output given presentation of a single input symbol from success class s_X.

Determination of $\left\{\sum_{\omega^{(X)}=0}^{2^\mu-1} p(\omega^{(X)}|m) I(\omega^{(Y)}, \omega^{(X)}, \theta, \mathcal{M}_j)\right\}_{\Theta,\mathcal{M}}$ in Eq. (14): Before derivation of this quantity we make some observations to guide us. Recall that this is defined as the expectation of the output symbol $\omega^{(Y)}$ probability given the input symbol $\omega^{(X)}$ running over only those input alphabet and connectivity combinations for which the output code $\omega^{(Y)}$ is realized. Imagine that we take a UBN with a fixed number of inputs and a fixed input alphabet and we attach a single output with random connections. We 'display' all input patterns on the MF inputs and evaluate the resulting empirical entropy of the output symbols after all N patterns are presented. We then randomly connect another output and repeat the procedure, before adding another and again repeating the procedure, thus growing the size of the output layer one unit at a time. As the number of network outputs increases, more entropy is recovered by the outputs from the inputs. Eventually, when all entropy is recovered the mapping f is one to one and the input symbol is fully determined by the output symbol. At this point, the empirical probability of some output symbol $p(\omega^{(Y)}|\theta, m)$ is either 0 (i.e. symbol $\omega^{(Y)}$ does not occur) or $p(\omega^{(X)}|m)$ where $\omega^{(X)}$ is the necessarily unique input symbol giving rise to $\omega^{(Y)}$. As we noted above, however, the average $\langle p(\omega^{(Y)})\rangle_{\Theta\mathcal{M}}$ inclusive of $\omega^{(Y)}$ that do not occur for a given θ and m tends to zero as $\gamma \to \infty$ for every symbol $\omega^{(Y)}$. This leads to an incorrect entropy since if we sum entropy over all possible $\omega^{(Y)} \in \Omega^{(Y)}$, $\langle H\rangle \to \infty$ which is incorrect since the entropy of the outputs cannot exceed the entropy of the inputs, which is bounded by the raw entropy of the events (where this bound is achieved if all $\omega^{(X)}$ are unique). For a small number of GCs every output symbol appears with finite probability (i.e.

all possible GC patterns are observable) and each of those symbols occurs in response to many input symbols. Hence, in this regime, output symbol probabilities are largely independent of the the connections and event mapping. However as the number of GCs increases, their output symbols appear only in response to a smaller number of input symbols. Hence output symbol occurrence becomes constrained by the specific connectivity of the network and choice of event mapping, until eventually single input symbols map uniquely to single output symbols in a manner that is completely determined by the specific network realization. As γ increases and this regime is approached, $\{p(\omega^{(Y)}|\theta,m)\} \sim p(\omega^{(X)}|m)$ becomes appropriate. Hence our definition of this quantity should exhibit this behavior in the limits of small and large γ. We first determine the conditional output symbol probability in the case that the output code exists,

$$
\begin{aligned}
p(\omega^{(Y)}|\theta,m) &= \sum_{\omega^{(X)}=0}^{2^{\mu}-1} p(\omega^{(X)}|m) I(\omega^{(Y)},\omega^{(X)},\theta,m) \\
&= \sum_{s_X=0}^{\mu} \sum_{\omega^{(X)} \in c^{(s_X)}_{\omega^{(Y)}}} p(\omega^{(X)}|m) \qquad \text{if } \omega^{(Y)} \text{exists; 0 otherwise} \qquad (18)
\end{aligned}
$$

where $c^{(s_X)}_{\omega^{(Y)}}$ is the set of input symbol indices for patterns in class s_X that map to $\omega^{(Y)}$. The number of symbols in this set is $n_c = \left|c^{(s_X)}_{\omega^{(Y)}}\right|$. Therefore to determine the expected conditional probability of occurrence of output code $\omega^{(Y)}$ (assuming that $\omega^{(Y)}$ exists for network θ and input alphabet m) the expectation of the non-zero contribution to Eq. (18) is computed,

$$\left\{\sum_{\omega^{(X)}=0}^{2^{\mu}-1} p(\omega^{(X)}|m) I(\omega^{(Y)}, \omega^{(X)}, \theta, m)\right\}_{\Theta,\mathcal{M}} = \sum_{\theta=0}^{2^{\mu\gamma}-1} p(\theta) \sum_{m\in\mathcal{M}} p(m) \sum_{s_X=0}^{\mu} \sum_{\omega^{(X)}\in c^{(s_X)}_{\omega^{(Y)}}} p(\omega^{(X)}|m)$$
$$= \sum_{\theta=0}^{2^{\mu\gamma}-1} p(\theta) \sum_{s_X=0}^{\mu} n_c p(\omega^{(s_X)})$$
$$= \sum_{s_X=0}^{\mu} p(s_X) \frac{\langle n_c\rangle_\Theta}{u^{s_X}_{Np(s_X)}}$$
$$= \sum_{s_X=0}^{\mu} p(s_X) a^{(s_X)}_{\gamma/s_Y} \quad (19)$$

where $a^{(s_X)}_{\gamma/s_Y}$ is the fraction of the input symbols in class s_X that lead to an output symbol $\omega^{(Y)}$ in class s_Y for a network having γ outputs ($u^{s_X}_{Np(s_X)}$ is defined in Eq. (10)). The average fraction of input symbols leading to some output symbol $\omega^{(Y)}$ over all network connectivities ($a^{(s_X)}_{\gamma/s_Y}$) depends on the number of active outputs (s_Y) in the output symbol (which is determined by each $\omega^{(Y)}$), and on the total number of outputs (γ), rather than on the specific connectivity θ or symbol $\omega^{(Y)}$. In the limit that $\gamma \to \infty$ only one input symbol can map to the output symbol ($a^{(s_X)}_{\gamma/s_Y} = 1/u^{s_X}_{Np(s_X)}$) and in this limit contributions to the output symbol probability from other input symbols must fall to zero. Therefore we may express this probability as a sum of the contribution due to the class of input symbols that $\omega^{(s_Y)}$ converges on as $\gamma \to \infty$, $a^{(s_X=s^*_X)}_{\gamma/s_Y}$ and contributions from the input classes whose contributions tend to zero this limit, $a^{(s_X\neq s^*_X)}_{\gamma/s_Y}$. Then,

$$\left\{\sum_{\omega^{(X)}=0}^{2^{\mu}-1} p(\omega^{(X)}|m) I(\omega^{(Y)}, \omega^{(X)}, \theta, \mathcal{M}_j)\right\}_{\Theta,\mathcal{M}} = p(s^*_X) a^{(s_X=s^*_X)}_{\gamma/s_Y} + \sum_{\beta\neq s_X} p(\beta) a^{(s_X\neq s^*_X)}_{\gamma/s_Y} \quad (20)$$

To simplify the initial problem of determining $a^{(s_X=s^*_X)}_{\gamma/s_Y}$ and $a^{(s_X\neq s^*_X)}_{\gamma/s_Y}$ we consider the case where $\gamma = s_Y$ such that we consider the output symbol for which all outputs are activated ($\omega^{(Y)} = 2^{\gamma-1}$). Following our preceding arguments we compute $a^{(s_X)}_{\gamma/s_Y}$ as $s_Y = \gamma \to \infty$. For only one output, $a^{(s_X)}_{1/1} = p(\nu = 1|\omega^{(s_X)})$; and assuming only that every additional output unit reduces $a^{(s_X)}_{\gamma/s_Y}$ by some constant proportion we can

specify the recursion relation (dropping the $a^{(s_X)}_{\gamma/s_Y}$ notation using $a^{(s_X)}_{s_Y}$ here instead since $s_Y = \gamma$),

$$a^{(s_X)}_{s_Y} = a^{(s_X)}_{s_Y-1} - r(a^{(s_X)}_{s_Y-1} - b) \tag{21}$$

where r is the proportion of input symbols eliminated from $c^{(s_X)}_{\omega^{(Y)}}$ (for $\omega^{(Y)} \in$ success class s_Y) by each active output and b is some minimal fraction remaining in the limit $\gamma \to \infty$. Differencing and then solving the relation in the same manner as in the previous section,

$$\begin{aligned} a^{(s_X)}_{s_Y} &= a^{(s_X)}_{s_Y-1}(2-r) - a^{(s_X)}_{s_Y-2}(1-r) \\ &= A + B(1-r)^{s_Y} \end{aligned} \tag{22}$$

where, when $s_Y = \gamma = 0$, $a^{(s_X)}_0 = A + B$, while when $s_Y = \gamma \to \infty$, $a^{(s_X)}_{s_Y} \to A$. Therefore, to compute $a^{(s_X = s^*_X)}_{s_Y}$ let $A = p(\omega^{(X)}|s^*_X) = 1/u^{s^*_X}_{Np(s^*_X)}$ and hence $B = a^{(s_X = s^*_X)}_0 - 1/u^{s^*_X}_{Np(s^*_X)}$, while to compute $a^{(s_X \neq s^*_X)}_{s_Y}$ let $A = 0$ and hence $B = a^{(s_X)}_0$. Setting the fraction of input symbols removed from the set that causes output symbol $\omega^{(Y)} = 2^{\gamma-1}$ as those input symbols that do not permit activation of the $s_Y = (\gamma+1)$th output, $r = 1 - p(\nu_{s_Y} = 1|\omega^{(s_X)})$ (i.e. the complement of the probability that the output is active given the input) and setting the initial fraction of input symbols equal to the fraction that activate the first output $a^{(s_X)}_0 = p(\nu_1 = 1|\omega^{(s_X)})$,

$$a^{(s_X = s^*_X)}_{s_Y} = 1/u^{s_X}_{Np(s_X)} + [p(\nu = 1|\omega^{(s_X)}) - 1/u^{s_X}_{Np(s_X)}]p(\nu = 1|\omega^{(s_X)})^{s_Y} \tag{23}$$

$$a^{(s_X \neq s^*_X)}_{s_Y} = p(\nu = 1|\omega^{(s_X)})^{s_Y}. \tag{24}$$

Note that the solution for the case in which the output symbol converges on some

input symbol satisfies the requirements we set earlier, whereas the alternative solution is exactly the standard solution for the probability of the realization of $\{1, 1, ..., 1\}$ throughout a group of uncorrelated random binary variables. Eq. (23) applies to the case where all outputs are active and takes as its initial condition $a_0^{(s_X)}$, the proportion of symbols in class s_X that cause a single output to be activated ($p(\nu = 1|\omega^{(s_X)})$).

We now adapt the expression to apply to any output symbol success class where $s_Y \neq \gamma$. For a pattern having s_Y active outputs and $\gamma - s_Y$ inactive outputs we apply Eq. (23) with the initial condition $a_0^{(s_X)}$ taken as the fraction of input symbols in class s_X causing $\omega^{(Y)}$, assuming s_Y active outputs only (i.e. the initial condition for the recurrence for the $\gamma - s_Y$ inactive outputs is the end condition $a_{s_Y}^{(s_X)}$ of Eq. (23) in the case of s_Y active outputs). Hence

$$a_{\gamma/s_Y}^{(s_X = s_X^*)} = 1/u_{Np(s_X)}^{s_X} + \left[[a_0^{(s_X)} - 1/u_{Np(s_X)}^{s_X}] p(\nu = 1|\omega^{(s_X)})^{s_Y} \right] \left(1 - p(\nu = 1|\omega^{(s_X)}) \right)^{(\gamma - s_Y)} \quad (25)$$

$$a_{\gamma/s_Y}^{(s_X \neq s_X^*)} = p(\nu = 1|\omega^{(s_X)})^{s_Y} \left(1 - p(\nu = 1|\omega^{(s_X)}) \right)^{(\gamma - s_Y)}. \quad (26)$$

Therefore, where subscript in refers to the inputs and subscript out refers to the outputs,

$$\begin{aligned}
\left\{ \sum_{\omega^{(X)}=0}^{2^\mu - 1} p(\omega^{(X)}) I(\omega^{(Y)}, \omega^{(X)}, \theta, \mathcal{M}_j) \right\}_{\Theta, \mathcal{M}} &= p(s_X^*) a_{s_Y}^{(s_X^*)} \\
&\quad + \sum_{\beta \neq s_X^*} p(\beta) p(\nu = 1|\omega_{in}^{(\beta)})^{s_Y} \left(1 - p(\nu = 1|\omega_{in}^{(\beta)}) \right)^{(\gamma - s_Y)} \\
&= p(s_X^*) a_{s_Y}^{(s_X^*)} + \sum_{\beta \neq s_X^*} \binom{\mu}{\beta} \binom{\gamma}{s_Y} p(\omega_{out}^{(s_Y)}, \omega_{in}^{(\beta)}) / \binom{\gamma}{s_Y} \\
&= p(s_X^*) a_{s_Y}^{(s_X^*)} + \sum_{\beta \neq s_X^*} p(s_Y, \beta) / \binom{\gamma}{s_Y} \quad (27)
\end{aligned}$$

The Entropy of the output symbols: For independent random binary inputs, $p(\omega^{(X)})$ depends only on the number of successes in each input pattern and the

number of events. Hence regrouping the input symbols in the sum of Eq. (16) across $\omega^{(X)}$ into input symbol success classes,

$$
\begin{aligned}
&\sum_{\omega^{(X)}=0}^{2^{\mu}-1} p(\omega^{(X)})p(\nu_1 = x_1|\omega^{(X)})...p(\nu_\gamma = x_\gamma|\omega^{(X)}) = \\
&\quad = \sum_{s_x=0}^{\mu}\left(\sum_{\omega^{(X)}\in c^{(s_X)}_{\omega^{(Y)}}} p(\omega^{(s_X)})p(\nu_1 = x_1|\omega^{(s_X)})...p(\nu_\gamma = x_\gamma|\omega^{(s_X)})\right) \\
&\quad = \sum_{s_x=0}^{\mu}\binom{\mu}{s_X} p(\omega^{(s_X)})p(\nu_1 = x_1|\omega^{(s_X)})...p(\nu_\gamma = x_\gamma|\omega^{(s_X)}) \\
&\quad = \sum_{s_x=0}^{\mu}\binom{\mu}{s_X} p(\omega^{(s_X)})p(\nu = 1|\omega^{(s_X)})^{s_Y}[1 - p(\nu = 1|\omega^{(s_X)})]^{(\gamma - s_Y)} \qquad (28)
\end{aligned}
$$

Combining this and other previous results,

$$
\begin{aligned}
H(\Omega^{(Y)}|\Theta,\mathcal{M}) &= -\sum_{\omega^{(Y)}=0}^{2^{\gamma}-1}\sum_{\omega^{(X)}=0}^{2^{\mu}-1}\left\langle p(\omega^{(X)})I(\omega^{(Y)},\omega^{(X)},\theta,m)\right\rangle_{\Theta,\mathcal{M}} \cdot \\
&\quad \cdot \log_2[\left\{\sum_{\omega^{(X)}=0}^{2^{\mu}-1} p(\omega^{(X)})I(\omega^{(Y)},\omega^{(X)},\theta,m)\right\}_{\Theta,\mathcal{M}}] \\
&= -\sum_{s_Y=0}^{\gamma}\binom{\gamma}{s_Y}\sum_{s_x=0}^{\mu}\binom{\mu}{s_X} p(\omega^{(s_X)})p(\nu = 1|\omega^{(s_X)})^{s_Y}[1 - p(\nu = 1|\omega^{(s_X)})]^{(\gamma - s_Y)} \cdot \\
&\quad \cdot \log_2[p(s_X)a_{s_Y}^{(s_X^*)} + \sum_{s_X\neq\beta} p(\beta, s_Y)/\binom{\gamma}{s_Y}] \\
&= -\sum_{s_Y=0}^{\gamma}\sum_{s_x=0}^{\mu} p(s_X,s_Y)\log_2[p(s_X)a_{s_Y}^{(s_X^*)} + \sum_{\beta\neq s_X} p(\beta, s_Y)/\binom{\gamma}{s_Y}]. \qquad (29)
\end{aligned}
$$

The direct method for computing the outputs of a UBN given an input (as outlined in section 2.6.1, above) takes a time (τ_M) proportional to the product of the dimensions of the connectivity matrix, $\mu\gamma$, and the number of events; hence $\tau_M \sim N\mu\gamma$. When implemented in optimized form the time (τ_A) required to compute the entropy with Eq. (29) scales as $\tau_A \sim (\mu + \gamma)^3$. For small numbers of patterns, $\tau_M < \tau_A$ and the direct method is the fastest way to determine the UBN

entropy. However, for a network of the size used in this study, taking $\tau_M = \tau_A$ and solving for N indicates that Eq. (29) outperforms the naive method when the number of events exceeds around 4000. In practice this value is much lower because τ_M does not account for the overhead in generating the random inputs or creating the empirical distribution and computing the Shannon information over that distribution. Since we computed our entropy estimate for 1×10^9 events, Eq. (29) provided a significant advantage. To gather our full dataset, however, still required significant computational resources due to our explicit search over a large number of points in parameter space; so we deployed our method using custom parallelized C code running on a compute cluster. Due to the large values of the arguments of the binomial coefficients in our calculation, standard floating point arithmetic would experience overflow errors. To prevent this we utilized multi-precision arithmetic. We avoided using an approximation such as Stirling's formula due to concern about accumulation of numerical errors when one or both arguments of the binomial coefficient are small.

3.2 Mathematical definitions used in the derivation of the entropy

Table S4, related to the Appendix to the Supplemental Experimental Procedures: Mathematical notation for the input layer

Symbol:	Meaning	Illustrative value
μ	Number of MF input nodes	176
$\hat{\chi}$	An **input pattern** (vector of binary MF values)	$0, 0, 1, 0, ..., 1$
X	The set of all possible input patterns	$\{\{0, 0, ..., 0\}, \{1, 0, ..., 0\}, ...\}$
N	Number of events encoded	1×10^9
$\omega^{(X)}$	**Input symbol:** Integer representation of an input pattern	12
$\Omega^{(X)}$	**Input alphabet:** Integer index set of some subset of input patterns	$\{12, 14, ..., 2\}$
p_{MF}	Probability that MF is active when a pattern is generated	0.1
$p(\hat{\chi})$	Probability of an input pattern sampled in response to an event	$0.1 \times 0.1 \times ... \times 0.9$
m	**Event mapping:** mapping from events to input symbols	$\{1 \to 12, ..., N \to 2\}$
$\mathcal{M}$	The set of all event mappings	$\{m_1, m_2, ..., m_Z\}$
$p(\omega^{(X)})$	Probability of occurrence of input symbol $\omega^{(X)}$	0.0001
$p(\omega^{(X)}\|m)$	Empirical input symbol probability given event mapping m	0.0001
I	Indicator function	$[0, 1]$
s_X	**Input success class:** Number active MFs in an input pattern	10
$u^{s_X}_{Np(s_X)}$	Mean number of unique codes in MF success class s_X after N events	174.2

Table S5, related to the Appendix to the Supplemental Experimental Procedures: Mathematical notation for the output layer

Symbol:	Meaning	Illustrative value
γ	Number of GC output nodes	509
$\hat{\nu}$	An **output pattern** (vector of binary GC values)	$0, 0, 1, 0, ..., 1$
d	**Number of GC synaptic connections:** Number MFs connected	4
ϕ	**GC threshold:** Number of active MF inputs for GC to activate	2
Y	Set of all possible output patterns	$\{\{0, 0, ..., 0\}, \{1, 0, ..., 0\}, ...\}$
$\omega^{(Y)}$	**Output symbol:** Integer representation of an output pattern	37
$\Omega^{(Y)}$	**Output alphabet:** Integer index set of some subset of output patterns	$37, 14, ..., 76$
$p(\nu = 1 \vert \omega^{(X)})$	GC activation probability given input symbol $\omega^{(X)}$	0.1
f	Mapping from input symbols to output symbols	$\Omega^{(X)} \rightarrow \Omega^{(Y)}$
θ	Integer indexing a single network realization	Large integers
$p(\omega^{(Y)} \vert \theta, m)$	Conditional empirical probability of $\omega^{(Y)}$ given network θ and m	0.0001
$H(\Omega^{(Y)} \vert \theta, m)$	Conditional entropy of $\Omega^{(Y)}$ given network θ and and m	29.9 bits
Θ	The set of all network realizations	$\{0, 1, ..., 2^{\mu\gamma-1}\}$
$H(\Omega^{(Y)} \vert \Theta, \mathcal{M})$	Expected conditional entropy of $\Omega^{(Y)}$ given networks Θ and event mappings $\mathcal{M}$	29.9 bits
$c_{\omega^{(Y)}}$	The set of input symbols that cause output symbol $\omega^{(Y)}$	$24, 10, ..., 17$
$c_{\omega^{(Y)}}^{(s_X)}$	The set of input symbols in class s_X that cause output symbol $\omega^{(Y)}$	$1, 4, ..., 64$
s_Y	**Output success class:** The number of active GCs in an output pattern	10
$a_{\gamma/s_Y}^{(s_X)}$	Mean fraction of input codes in s_X leading to output codes in s_Y for γ GCs	0.001
$a_{\gamma/s_Y}^{(s_X^*)}$	As $a_{\gamma/s_Y}^{(s_X)}$ when output symbol occurs for input symbol in s_X when f is $1:1$	0.001
$a_{\gamma/s_Y}^{(s_X \emptyset)}$	As $a_{\gamma/s_Y}^{(s_X^*)}$ but output symbol does not occur for s_X when f is $1:1$	0
$p(s_X, s_Y)$	The joint distribution of input and output pattern success classes	Matrix

Supplemental References

Arthur, D. & Vassilvitskii, S. (2007). K-means++: The advantages of careful seeding. In Proceedings of the Eighteenth Annual ACM-SIAM Symposium on Discrete Algorithms, SODA '07, pp. 1027–1035. (Philadelphia, PA, USA: Society for Industrial and Applied Mathematics).

Brandes, U., Eiglsperger, M., Herman, I., Himsolt, M., & Marshall, M. (2002). Graphml progress report structural layer proposal. In Graph Drawing, P. Mutzel, M. Jünger, & S. Leipert, eds., vol. 2265 of *LNCS*. (Springer Berlin Heidelberg), pp. 501–512.

Collette, A. (2013). Python and HDF5. (O'Reilly Media).

Cover, T. M. & Thomas, J. A. (1991). Elements of information theory. (New York: Wiley).

Deb, K. & Padhye, N. (2010). Development of efficient particle swarm optimizers by using concepts from evolutionary algorithms. In Proceedings of the 12th annual conference on Genetic and evolutionary computation, pp. 55–62. ACM.

Garrett, A. L. (2014). inspyred. http://inspyred.github.com/.

Gundersen, H., Bagger, P., Bendtsen, T., Evans, S., Korbo, L., Marcussen, N., Moller, A., Neilsen, K., Nyengaard, J., & Pakkenberg, B. (1988). The new stereological tools: disector, fractionator, nucleator and point sampled intercepts and their use in pathological research and diagnosis. Apmis, 96, 857–881.

Hagberg, A. A., Schult, D. A., & Swart, P. J. (2008). Exploring network structure, dynamics, and function using NetworkX. In Proceedings of the 7th Python in Science Conference (SciPy2008), pp. 11–15. Pasadena, CA USA.

Ince, R. A. A., Petersen, R. S., Swan, D. C., & Panzeri, S. (2009). Python for information theoretic analysis of neural data. Front. Neuroinform., 3, 4.

Lloyd, S. (1982). Least squares quantization in pcm. IEEE Trans. Inf. Theory, 28, 129–137.

Nemenman, I., Shafee, F., & Bialek, W. (2002). Entropy and inference, revisited. In NIPS 14. (MIT Press).

Panzeri, S., Senatore, R., Montemurro, M. A., & Petersen, R. S. (2007). Correcting for the sampling bias problem in spike train information measures. J Neurophysiol, 98, 1064–1072.

Panzeri, S. & Treves, A. (1996). Analytical estimates of limited sampling biases in different information measures. Network - Comp. Neural., 7, 87–107.

Pedregosa, F., Varoquaux, G., Gramfort, A., Michel, V., Thirion, B., Grisel, O., Blondel, M., Prettenhofer, P., Weiss, R., Dubourg, V., Vanderplas, J., Passos, A., Cournapeau, D., Brucher, M., Perrot, M., & Duchesnay, E. (2011). Scikit-learn: Machine learning in Python. JMLR, 12, 2825–2830.

Rothman, J. S. & Silver, R. A. (2014). Data-driven modeling of synaptic transmission and integration. In Computational Neuroscience, K. T. Blackwell, ed., vol. 123 of *Progress in Molecular Biology and Translational Science*. (Academic Press), pp. 305 – 350.

Sargent, P. B., Saviane, C., Neilsen, T. A., DiGregorio, D. A., & Silver, R. A. (2005). Rapid vesicular release, quantal variability, and spillover contribute to the precision and reliability of transmission at a glomerular synapse. J. Neurosci., 25, 8173–8187.

Sawtell, N. B. (2010). Multimodal integration in granule cells as a basis for associative plasticity and sensory predication in a cerebellum-like circuit. Neuron, 66, 573–584.

Strong, S. P., Koberle, R., de Ruyter van Steveninck, R. R., & Bialek, W. (1998). Entropy and information in neural spike trains. Phys. Rev. Lett., 80, 197–200.

Tsodyks, M. & Markram, H. (1997). The neural code between neocortical pyra-

midal neurons depends on neurotransmitter release probability. PNAS, 94, 719–723.
