# Supplementary material for: Network Structure within the Cerebellar Input Layer Enables Lossless Sparse Encoding
Source: Neuron. 2014 Aug 20;83(4):960–74. doi: 10.1016/j.neuron.2014.07.020 (PMC4148198; doi:10.1016/j.neuron.2014.07.020)
Supplement: Document S2. Article plus Supplemental Information [file mmc2.pdf]

CellPress

Neuron
Article

Network Structure within the Cerebellar Input Layer Enables Lossless Sparse Encoding

Guy Billings,[1] Eugenio Piasini,[1] Andrea Lőrincz,[2] Zoltan Nusser,[2] and R. Angus Silver[1,*]
[1]Department of Neuroscience, Physiology and Pharmacology, University College London, London, WC1E 6BT UK
[2]Institute of Experimental Medicine, Hungarian Academy of Sciences, H-1083 Budapest, Hungary
*Correspondence: a.silver@ucl.ac.uk
http://dx.doi.org/10.1016/j.neuron.2014.07.020

SUMMARY

The synaptic connectivity within neuronal networks is thought to determine the information processing they perform, yet network structure-function relationships remain poorly understood. By combining quantitative anatomy of the cerebellar input layer and information theoretic analysis of network models, we investigated how synaptic connectivity affects information transmission and processing. Simplified binary models revealed that the synaptic connectivity within feedforward networks determines the trade-off between information transmission and sparse encoding. Networks with few synaptic connections per neuron and network-activity-dependent threshold were optimal for lossless sparse encoding over the widest range of input activities. Biologically detailed spiking network models with experimentally constrained synaptic conductances and inhibition confirmed our analytical predictions. Our results establish that the synaptic connectivity within the cerebellar input layer enables efficient lossless sparse encoding. Moreover, they provide a functional explanation for why granule cells have approximately four dendrites, a feature that has been evolutionarily conserved since the appearance of fish.

INTRODUCTION

Different regions of the brain exhibit distinct anatomical structures, cell morphologies, and synaptic connectivities and perform specific computational tasks. However, linking the structure to function (Honey et al., 2007) or dysfunction (Dyhrfjeld-Johnsen et al., 2007) has proved difficult, because the synaptic connectivity, neuronal properties, and the computations performed are usually poorly defined. Some notable exceptions exist in circuits where the function is clear. In the retina, asymmetric spatial patterns of synaptic input onto starburst amacrine cells contribute to direction selectivity (Briggman et al., 2011). In mouse primary visual cortex, neurons with similar orientation selectivity have been shown to be preferentially connected (Ko et al., 2011). In pattern generator circuits within the spinal cord, distinct neuronal subtypes compute different gaits during locomotion (Talpalar et al., 2013). Despite these advances, the contribution that synaptic connectivity makes to information processing remains unclear in most brain regions.

The cerebellar cortex is particularly well suited to network structure-function analysis due to its relatively simple three layer structure, few neuronal cell types, and its well-established role in motor control (Eccles et al., 1967). Moreover, there is wide consensus that the cerebellar input layer, or granule cell layer (GCL), transforms mossy fiber (MF) inputs, conveying sensory and efferent copy information, into a higher dimensional, sparser code (Marr, 1969). This increases the separation between the patterns (Olshausen and Field, 2004), thereby enabling downstream cerebellar circuits to perform more effective associative learning (Albus, 1971; D'Angelo and De Zeeuw, 2009; Marr, 1969; Medina and Mauk, 2000; Schweighofer et al., 2001; Tyrrell and Willshaw, 1992), adaptive filtering (Fujita, 1982), and binary addressing (Kanerva, 1988). Three basic properties are required for divergent feedforward networks to perform effective pattern separation: (1) information is conserved, (2) the dimensionality of the output coding is larger than that of the input, and (3) the output code is sparse. However, the contribution that synaptic connectivity makes to these functions remains poorly understood.

To investigate how the network structure of the cerebellar input layer affects its function, we first quantified specific anatomical properties of the network. We then developed a simplified model of the GCL that was analytically tractable, allowing us to quantify information transmission and sparse encoding in networks with different synaptic connectivities. Finally, we tested predictions from our analytical approach on the relationship between network structure and function using biologically detailed network models of spiking neurons, whose parameters were constrained by experimental measurements. Our results show that the synaptic connectivity within the cerebellar input layer, where GCs receive an average of approximately four excitatory MF inputs, is well suited for performing sparse encoding without loss of information.

RESULTS

Quantification of the Cerebellar Input Layer Structure and Development of a 3D Model of Excitatory Network Connectivity

Cerebellar MFs form large en passant presynaptic structures called rosettes that form the core of each synaptic glomerulus,

CrossMark

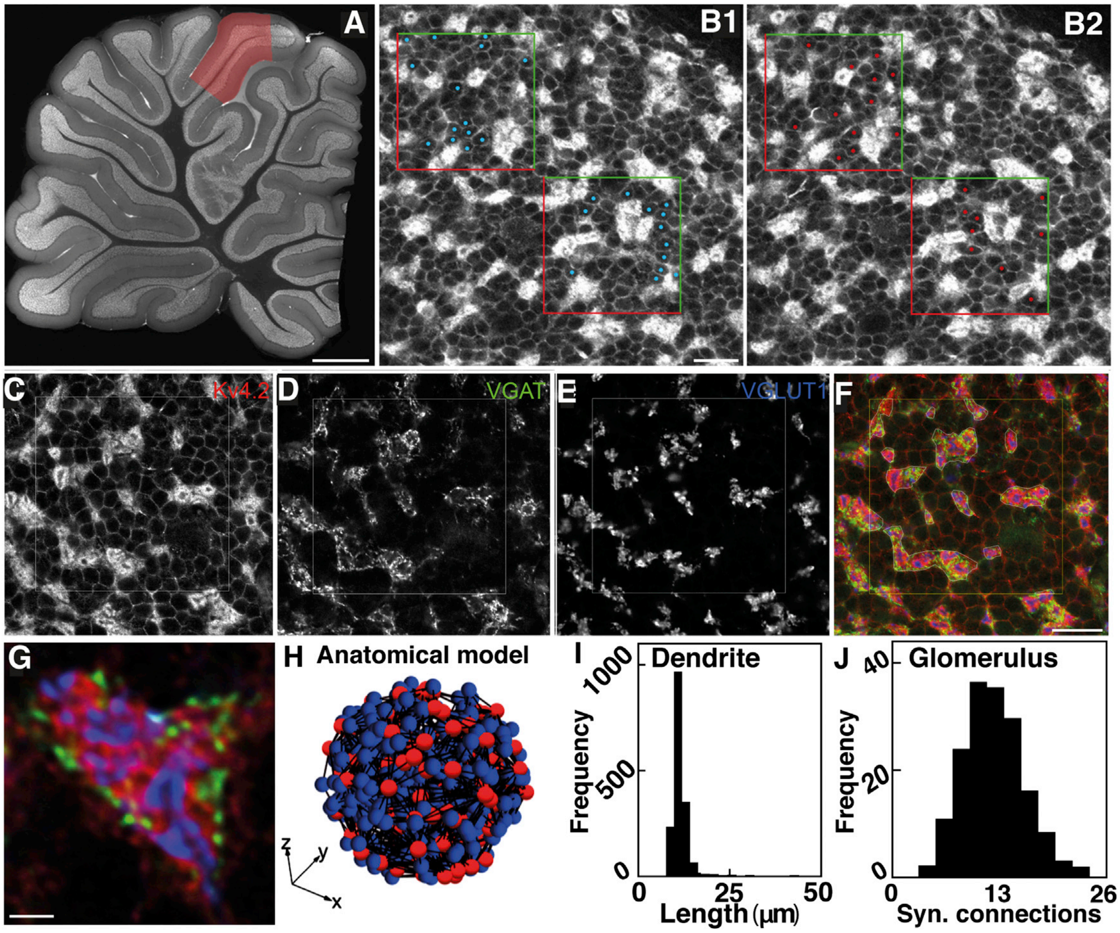

Figure 1. Granule Cell and Glomerular Density in the Rat Cerebellum and Construction of a Local Granule Cell Layer Model

(A) Measurements performed in lobule VIa (red area) of a parasagittal slice of cerebellar vermis.

(B) Regions of granule cell layer (GCL) with pairs of sections (left and right) and frames used for the unbiased counting method (cells on green edges are counted and on red edges excluded).

(C–E) Area of GCL immunolabeled for Kv4.2 (C) showing circular GC somatic outlines, VGAT (D), and VGLUT1 (E).

(F) Overlay of immunolabels for Kv4.2 (red), VGAT (green), and VGLUT1 (blue).

(G) Colabeling of the three markers used to demarcate a glomerulus.

(H) 3D anatomically constrained model of the local GCL network, consisting of a 40-μm-radius ball of glomeruli (red) and GCs (blue) with four dendrites per GC (black lines).

(I) Distribution of GC dendrite length in the local GCL network model.

(J) Distribution of the number of GC dendrites per MF rosette. (C)–(F) are at the same magnification, with scale bar on (F) applying to all panels. Scales, 1 mm in (A), 20 μm in (B)–(F), and 2 μm in G.

which also consists of Golgi cell axons, GC and Golgi cell dendrites, and a glial coat. While quantitative anatomical data are available on several cellular components across species (Harvey and Napper, 1988), the rosette-to-GC expansion ratio remains uncertain. To address this, we combined high-resolution confocal microscopy, multicolor immunofluorescence labeling, and an unbiased counting method to study the properties of the cerebellar GC layer in rat (Figures 1A and 1B). Immunolabeling for Kv4.2 delineated somatic plasma membranes and the dendrites of GCs (Figure 1C). GCs had a mean diameter of 6.72 ± 0.13 μm (n = 24) and mean density of 1.9 ± 0.14 × 10^6 mm^{-3}, similar to that previously reported (Harvey and Napper, 1988). Golgi cell axons and MF rosettes were identified with VGAT and VGlut1 immunolabeling, respectively (Figures 1D and 1E). Colabeling for all three molecules was used to identify glomeruli (Figures 1F and 1G), which occupied 28.8% ± 2.3% of the input layer volume and occurred at a density of 6.6 ± 1.5 × 10^5 mm^{-3} (Figure S1 available online; Tables S1 and S2). The local glomeruli-to-GC and thus rosette-to-GC ratio is therefore 1:2.9.

We examined the likely spatial extent of a local GC layer network by building a 3D anatomical model of MF-GC connectivity (Figure 1H), using our measured parameters together with existing measurements of MF rosette spacing (20 μm parasagittal and 60 μm mediolateral; Sultan, 2001). The claw-like ending of each GC dendrite contacts a single MF rosette. GC dendrites

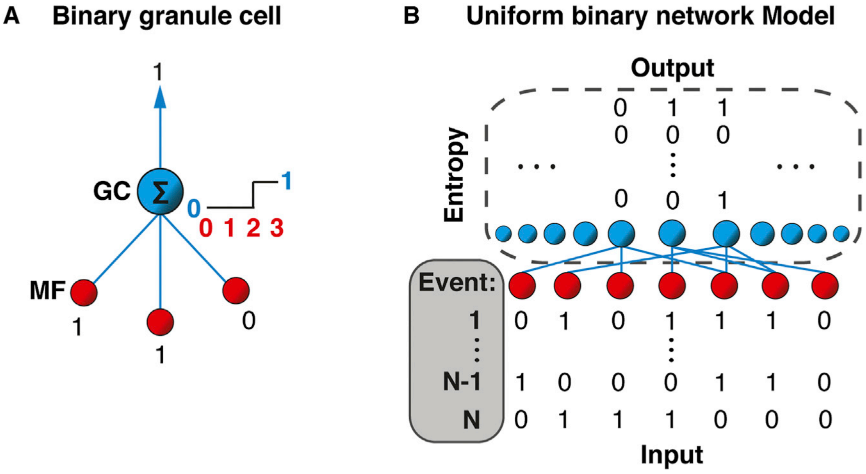

Figure 2. Schematic Representation of the Uniform Binary Network Model

(A) Schematic diagram showing binary mossy fiber (MF) synaptic inputs (red) and a linear thresholding binary granule cell (GC, blue) with a single MF synaptic connection per dendrite (blue lines). GC output is 1 if the sum of its MF input values is equal to the threshold or greater and 0 otherwise.

(B) Uniform binary network model is a random bipartite graph consisting of binary MFs and linear threshold GC units. Network with 3 MF synaptic connections per GC (d = 3), shown only for the three central GCs for clarity. N events, encoded as binary MF input patterns, are transformed into a binary GC output patterns. GC population entropy is calculated from the distribution of output patterns.

rarely exceed 30 μm (e.g., 4% in the cat; Palay and Chan-Palay, 1974; Palkovits et al., 1972), making the likelihood of a GC being innervated by two or more rosettes from the same MF low (Livet et al., 2007). The number of GC dendrites is therefore equal to the number of MF synaptic connections per GC (d). Moreover, the MFs that converge onto a GC typically arise from multiple precerebellar nuclei (Huang et al., 2013). To recreate these conditions, model GCs were placed at random in a sphere at the measured anatomical density within a central subfield of MFs to minimize edge effects (Figure 1H). Each model GC made synaptic connections to randomly selected MF rosettes, with the constraint that the dendritic length should be close to 15 μm. In practice, they rarely exceeded 20 μm (Figure 1I), as observed experimentally. Since the maximum distance between two GCs that could share the same MF input was ~40 μm, the largest ball of tissue that could be expected to have independent inputs was 80 μm in diameter, which is comparable to the thickness of the GC layer in rodents. Our anatomically constrained model of this "local GCL network" contained 176 MF synaptic rosettes and 509 GCs (Figure 1H). For an average of four dendrites per GC (Eccles et al., 1967), a single MF rosette made synaptic connections with an average of 12 different GCs (Figure 1J), consistent with estimates of 15–20 in monkey and cat (Eccles et al., 1967).

Uniform Binary Network Model for Computing Information Transmission

Direct calculation of information transmission for all possible configurations of MF input drive and network structure is computationally intractable. We therefore simplified our local GCL network model to permit mathematical analysis by removing the spatial dependences in the synaptic connectivity but conserving the random nature of the connectivity, the MF rosette-to-GC expansion ratio, and the number of MF synaptic connections per GC (Supplemental Information; Figure S2). In addition, we reduced rate-coded signals in MFs and GCs to binary representations, where 0 and 1 represent quiescence and activity, respectively. When a spatial pattern of binary MF activity is presented, each GC sums its equally weighted inputs and compares the sum to its threshold value. The GC output is 1 if the sum exceeds the threshold and 0 otherwise (Figure 2A). We refer to these binary MF and GC networks as the "uniform binary network model" or UBN model. Figure 2B shows a schematic illustration of such a model with three synaptic connections per GC. Sensory-motor "events" are represented as random binary MF activity patterns. Each one is thresholded by the GCs and transformed into a binary GC output pattern, from which the information encoded by the GC population can be calculated.

Although the simplifying assumptions required for calculating information in this manner are substantial, the cerebellar GCL is particularly amenable to this approach. Since the dimensionality of sensory-motor sample space is vast, we considered raw input as being nonrepeating from event-to-event, with each sensory-motor event being directly mapped to an MF input pattern. Real MF activity patterns encoding sensory-motor events consist of two main stochastic components: the subset of MFs in the network that were activated and the trial-to-trial variability in the spiking, synaptic transmission, and membrane noise present in an individual connection. In the UBN model, we consider the simplified case where the variance is dominated by the MF patterns themselves and the stochasticity of transmission is negligible, because fluctuations are averaged over multiple release sites and are integrated by GCs. Indeed, GCs have intrinsic and synaptic properties that make them well-suited to a binary representation because: (1) their soma and dendrites form a single electrical compartment, thereby acting as a point neuron (Silver et al., 1992), (2) much of their excitatory drive is composed of slow spillover-mediated AMPAR and NMDAR conductances that build up over time during rate-coded MF input (Arenz et al., 2008; DiGregorio et al., 2002; Schwartz et al., 2012), (3) GCs have only two to seven excitatory MF inputs, and (4) multiple MF inputs are typically required to reach spike threshold (Jörntell and Ekerot, 2006; Schwartz et al., 2012). Thus, GC activity reflects a thresholded version of a few active MF inputs, making the simplification to a thresholded binary representation (Figure 2A) more reasonable than for cells with larger numbers of inputs.

To quantify information transmission across UBN models, we developed an analytical method for rapidly calculating Shannon

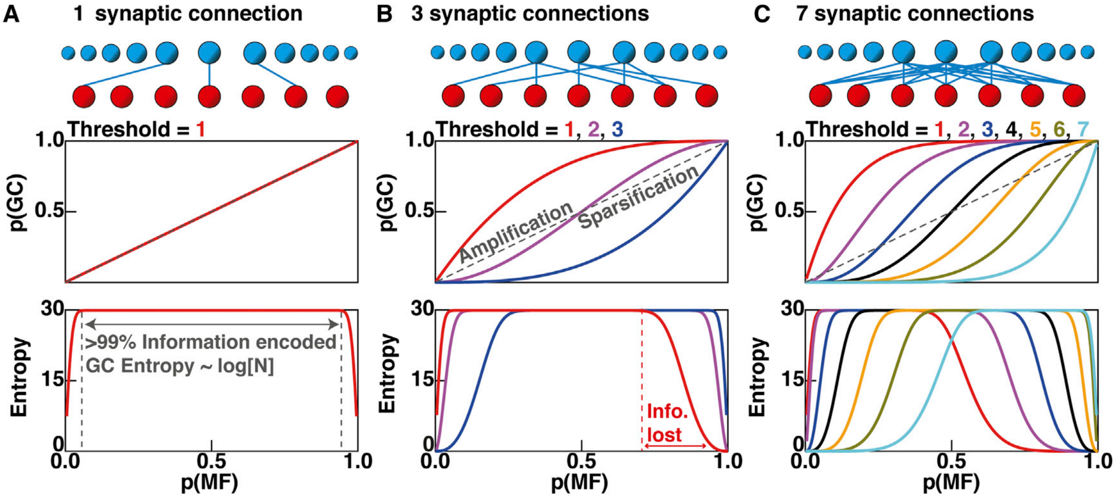

Figure 3. Number of Synaptic Connections per Neuron and Threshold Determine the Transmission and Transformation of Information in a Uniform Binary Network Model

(A) Top: schematic illustration of mossy fibers (MF, red) and granule cells (GCs) (blue) for a binary network with one MF synaptic connection per GC (d = 1; blue lines, shown for 3 GCs only). Middle: GC activation probability ($p(GC)$) as a function of MF activation probability ($p(MF)$), red line for a threshold of 1. Gray dashed line indicates $p(GC) = p(MF)$. Bottom: information (entropy) in GC population as a function of $p(MF)$ for one billion events. Vertical dashed lines indicate range of $p(MF)$ where >99% of the information is encoded by the GC population.

(B) Same as for (A) but for a network with d = 3 and all possible threshold values (1-3 red-blue).

(C) Same as for (B) but for d = 7 (1-7 red-cyan).

Information (Shannon, 1948), which corresponded to the entropy in the GC population under noise-free conditions (Figure 2B; Supplemental Information: Appendix, Equation 29). This allowed us to explore how information transmission depends on the synaptic connectivity of randomly connected networks, GC threshold, and the fraction of MFs active. In the limited cases where direct calculation of entropy was possible, it gave similar results to our analytical method (Figure S3). Although our analytical method can be used to calculate information transmission for the full set of MF input patterns (i.e., $2^{176} \approx 10^{53}$), we restricted the number of patterns to the maximum number of events a local GCL network could encode in the lifetime of a small rodent in the wild. This is approximately one billion, if we assume that events are integrated in ~30 ms windows (Figure S4B; Schwartz et al., 2012; van Beugen et al., 2013) for a lifetime of 1 year.

Effect of Synaptic Connectivity and Neuronal Threshold on the Transmission and Transformation of Activity Patterns in Uniform Binary Network Models

We first analyzed the functional properties of the simplest configuration of the UBN model, with one synaptic connection per GC and a threshold of 1. Since GCs are 3-fold more numerous than MF synaptic rosettes, the absolute number of activated GC was higher than the number of MF inputs. Despite this expansion, the fraction of active MFs (or MF activation probability [$p(MF)$]) was equal to the fraction of active GCs (or GC activation probability [$p(GC)$]) (Figure 3A, middle), as expected for a simple relay network. The relationship between GC entropy and $p(MF)$ had a truncated flat top because the information contained in the GC output patterns (Figure 3A) was limited by the maximum one billion unique MF input patterns and thus had a maximal value of 29.9 bits (i.e., $\log_2(10^9)$). We defined such flat, saturated sections of the entropy versus $p(MF)$ curve (Figure 3A, bottom) as full information transmission or lossless encoding regions (i.e., >99% of the event information). These calculations show that randomly connected feedforward networks with unitary synaptic connectivity can transmit all MF event information to GCs over a wide range of input activity. This is possible because the network operates far below the maximum transmission capacity of either its input or output (179 bits and 509 bits, respectively). However, this network does not perform sparse encoding.

For networks with more than one MF input per GC, the number of inputs required to reach threshold was a key variable, since it determined the fraction of GCs activated. Networks with three synaptic connections per GC had three possible threshold settings and thus three mappings from MF to GC activity (Figure 3B). For a threshold of 1, the fraction of active neuronal elements used to represent information was larger for the GC output than the MF input ($p(GC) > p(MF)$) over the whole MF input activity range (red line in Figure 3B, middle). Increasing the threshold to 2 produced GC activity that approximately matched the MF input activity, while increasing the threshold to 3 reduced the GC activity below that of the MFs across the whole range of input activity. Such sparsening of MF activity (i.e., $p(GC) < p(MF)$) is a key proposed function of the GCL (Albus, 1971; Marr, 1969).

At low threshold, full transmission of event information was achieved at low and intermediate MF input activities, but information was lost at high MF activities (Figure 3B, bottom, red). In contrast, at the highest threshold the lossless encoding range was shifted to higher MF activities (Figure 3B, bottom, blue). An intermediate threshold of 2 produced lossless encoding over

nearly the whole MF activity range but did not perform effective sparsification. Setting the number of MF inputs per GC to seven increased the number of possible mappings between MFs and GCs (Figure 3C). Moreover, at high thresholds, sparsification became highly pronounced (i.e., $p(GC) << p(MF)$), but the range of MF activation over which lossless encoding occurred was markedly reduced (Figure 3C, bottom). These results show that both synaptic connectivity and thresholding within the networks have a big impact on both the transmission and transformation of information. The inability of some networks to transmit information in particular $p(MF)$ regions is likely to be highly disadvantageous, because in vivo recordings show that MFs exhibit a wide range of activity levels (Arenz et al., 2008; Rancz et al., 2007; van Kan et al., 1993). On the other hand, full information transmission without sparsification is also problematic, because the GCL circuit then fails to perform its main function.

Trade-Off between Information Transmission and Sparsification in Uniform Binary Network Models with Fixed GC Threshold

To investigate further how network connectivity affects information transmission and sparsification, we examined the case of a relatively high fixed threshold, since GCs typically require activation of three of their four MF inputs to fire (Jörntell and Ekerot, 2006; Schwartz et al., 2012) and spike threshold is dominated by the presence of a large tonic inhibitory conductance (Brickley et al., 1996; Duguid et al., 2012). To do this, we set the threshold to 75% of the number of synaptic connections per neuron or as close to it as discretisation allowed (using a ceiling function). For such fixed threshold networks, the lossless encoding range decreased with increasing numbers of inputs (Figure 4A).

In contrast to information transmission, GC sparseness tended to increase with the number of synaptic connections per GC, particularly at low-to-intermediate levels of MF activation (Figures 4B and 4C). Figures 4D and 4E illustrate how two networks with different numbers of synaptic connections per GC transform MF input at $p(MF) = 0.3$. While the GC activity of the network with 20 synaptic connections was substantially lower than that with 3, the highly connected network was unable to transmit information at low-to-intermediate values of $p(MF)$. When $p(MF)$ was increased to 0.8, the high connectivity network became effective at transmitting information but did not encode the MF input more sparsely (Figure 4F). These results clearly demonstrate a trade-off between transmission and sparsening of input representations in simple feedforward networks and that synaptic connectivity and threshold determine the balance between these two competing functions.

To gain further insight into how the transmission-sparsification trade-off arises, we examined the relationship between the mean activity of the GC population and the mean activity of the MF population for different network connectivities (Figure 4C). For a single MF connection per GC and a threshold of 1, the network transfer function was linear, but as the number of connections increased the relationship became increasingly nonlinear. This lowered $p(GC)$ across a wide range of $p(MF)$, resulting in greater sparsification. However, at these low activation levels, the GC population could not encode all the MF patterns and thus information was lost.

Extension of Uniform Binary Network Models to Include Network-Activity-Dependent Thresholds

Golgi cells provide network-activity-dependent inhibition of GCs, via feedforward (Kanichay and Silver, 2008) and feedback inhibition (Cesana et al., 2013), although the impact of this phasic and spillover-mediated component is much weaker than tonic inhibition (Duguid et al., 2012). To mimic this activity-dependent change in inhibition in the UBN model, we implemented a network-activity-dependent threshold (NADT), which scaled the GC threshold in proportion to $p(MF)$ (Experimental Procedures). For a network with seven synaptic inputs per GC (Figure 5A, top), a low NADT and an initial threshold of 1 resulted in an amplification (i.e., $p(GC) > p(MF)$) and only permitted lossless encoding over the lower portion of $p(MF)$ (Figure 5A, cyan). Similar behavior was observed across all network connectivities, except those with few connections, which transmitted information across a wider range of $p(MF)$ (Figure 5B1). However, none of the networks with low NADT performed sparsification, when averaged across $p(MF)$ (Figure 5B2).

An NADT value of 1 enabled lossless transmission over nearly the entire MF input range for all networks tested, irrespective of the number of synaptic connections per GC (Figure 5C1). This was achieved by maintaining the GC activity around 0.5 (Figure 5C2), thereby maximizing encoding capacity. However, this strategy compromised sparsification. Increasing the NADT to high levels sparsened the GC representation (Figure 5D2) but introduced a lossy region for intermediate levels of MF activity for networks with more than 5 MF inputs per GC (Figure 5D1). These results show that unity NADT can enhance information transmission through feedforward networks with large numbers of synaptic connections per GC, but higher levels of NADT are required to sparsen input activity. However, only networks with small numbers of connections can perform lossless sparse encoding with high NADT, due to the network connectivity-dependent trade-off between information transmission and sparsification.

Identification of Optimal Network Connectivities for Robust Lossless Sparse Encoding

Since both information transmission and sparsification are essential for cerebellar operation, we investigated which feedforward network connectivity and threshold settings provided the best trade-off between these two competing functions. To do this, we analyzed our data set to find all network configurations that transmitted information without loss over the widest range of $p(MF)$ and transformed MF patterns into a sparser GC representation. Figure 6A shows the range of $p(MF)$ over which networks with different numbers of connections can transmit information losslessly and sparsify it for the fixed relative threshold case (75% of inputs). A comparable pattern was observed for networks with high NADT, but in these cases sparsening of the encoding was more effective at high $p(MF)$ (Figure 6B). Combining a high initial threshold and NADT provided an effective strategy for sparse lossless encoding in networks with few synaptic connections (Figure 6C). To identify the best-performing networks, we found those networks where sparsification could not be improved without detriment to the range of $p(MF)$ over which full information transmission was achieved and,

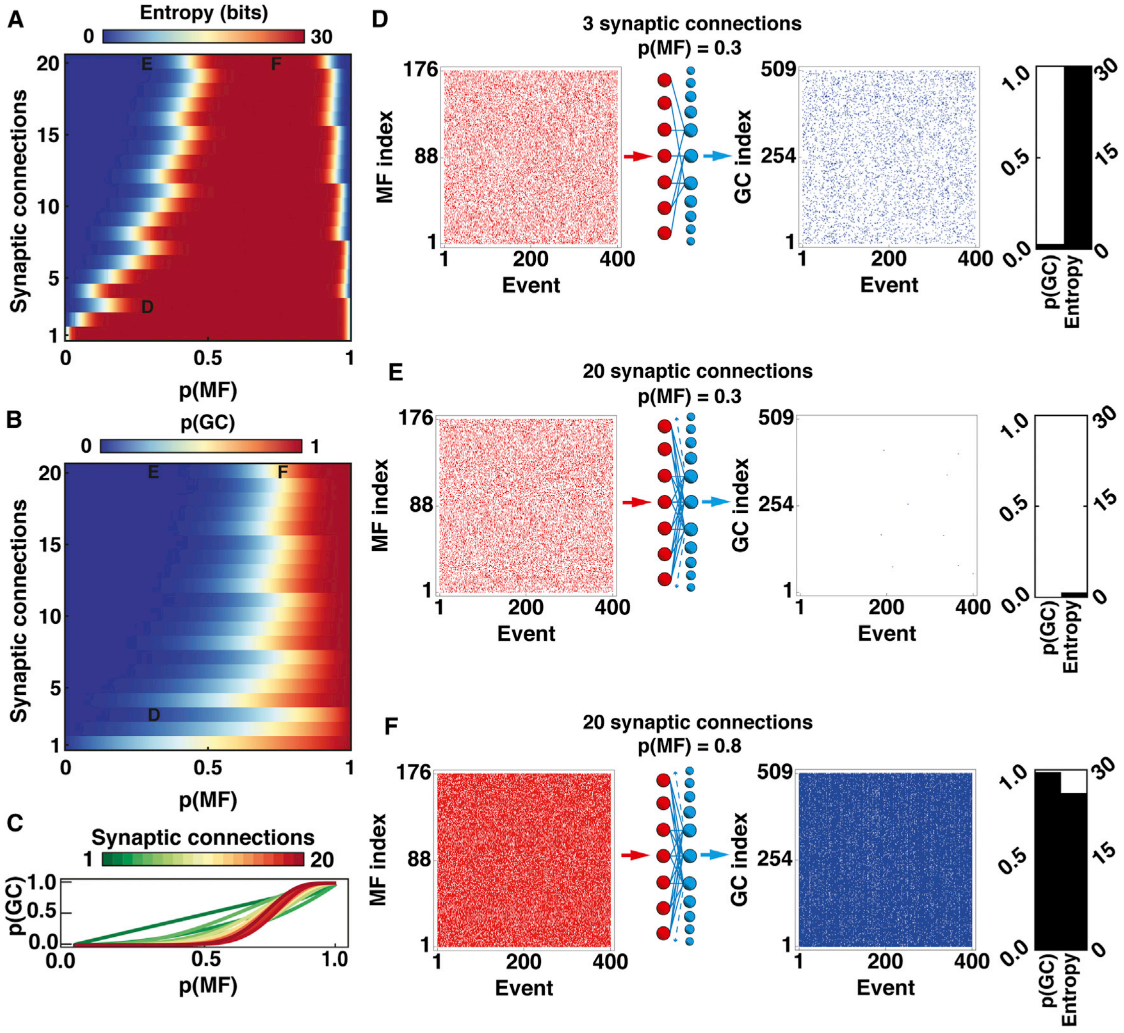

Figure 4. Effect of Synaptic Connectivity on Information Transmission and Population Activity in Uniform Binary Network Models with a Fixed Relative Threshold

(A) Quantity of event information (entropy) encoded by the granule cell (GC) population across the full range of mossy fiber (MF) activation probability *p(MF)* for uniform binary network models with different numbers of synaptic connections per GC (*d*) and a fixed relative threshold $\varphi = ceiling\ [0.75 \times d]$.

(B) GC activation probability *p(GC)* for the same network configurations as in (A).

(C) Same as for (B) but visualized as a line plot to show the relationship between *p(GC)* and *p(MF)* for different models.

(D) From left to right: a sample of 400 MF input patterns (events) with *p(MF)* = 0.3, where active MFs are red and inactive MFs are white, schematic network representation and GC output activity patterns (blue raster plot) for a network with *d* = 3 (see label D in panels A and B). Bar graph indicates *p(GC)* and entropy for one billion patterns, as for (A).

(E) Same as for (D) but for *d* = 20 (see label E in panels A and B).

(F) Same as for (D) but for *d* = 20 and *p(MF)* = 0.8 (see label F in panels A and B).

conversely, the lossless *p(MF)* range could not be improved without detriment to sparsification. For the fixed threshold case, networks with two to three MF inputs per GCs performed sparse encoding over the widest range of *p(MF)* without loss of information (Figure 6D). As the number of synaptic connections per GC increased, the sparse encodable range declined due to information loss, with little improvement in average sparsification. Networks with NADT = 2, or with a high initial threshold combined with NADT, that best performed lossless sparse encoding over the largest *p(MF)* range also had few synaptic connections, with four being particularly effective (Figure 6D). As the number of dendrites increased further, the sparse encodable range fell steeply indicating that encoding became lossy.

Our analysis of UBN models predict that few synaptic connections per GC provide the best trade-off between information transmission and sparse encoding over a wide range of MF input activity. However, the simplifications required to develop our analytical treatment raise the question of whether these predictions are valid for biological networks, where sensory-motor signals are encoded by MF firing rate and synaptic conductances, and GC spike threshold is set by a tonic inhibitory conductance.

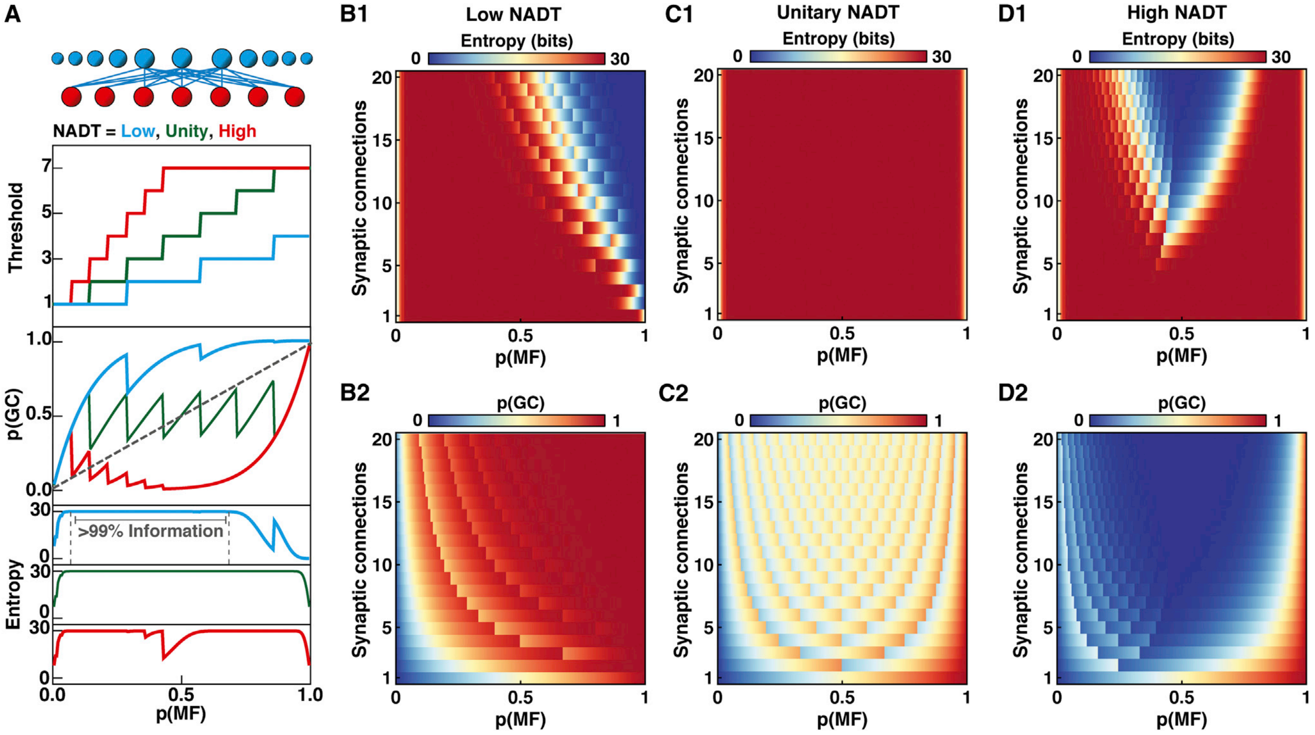

Figure 5. Effect of Activity-Dependent Threshold Regulation on the Trade-Off between Information Transmission and Sparsification
(A) Top: uniform binary network model schematic with mossy fibers (MFs) in red and granule cells (GCs) in blue (top); GC network-activity-dependent threshold (NADT) for low (0.5, blue), unity (1.0, green), and high (2.0, red) NADT, for a network with seven synaptic connections per GC ($d = 7$; connections for center 3 GCs shown for clarity). Middle: GC activation probability ($p(GC)$) versus MF activation probability ($p(MF)$) for the NADT functions above. Bottom: information encoded by GCs for each threshold function.
(B1 and B2) Information encoded by GCs and $p(GC)$, respectively, for low NADT networks with different d.
(C1 and C2 and D1 and D2) Same as for (B1 and B2) for unity NADT and high NADT, respectively.

A Biologically Detailed Network Model of the Cerebellar Input Layer

To test the validity of the predictions from our simple analytical model, we constructed a biologically detailed network model where each parameter was constrained by experimental measurements. We used the anatomically constrained local GC layer network model (Figure 1H), which captured the measured densities of MF synaptic rosettes and GCs and the spatial dependence of synaptic connectivity imposed by the finite length of GC dendrites. For each network configuration, we used a fixed instantiation of the randomly generated connectivity. GCs in the model were conductance based integrate-and-fire neurons with a capacitance (3.22 pF), input resistance (0.94 GΩ), and resting potential (−79.9 mV) set to the mean value obtained from GCs recorded at physiological temperature (Rothman et al., 2009; Schwartz et al., 2012). Information was represented in each MF input by the rate of an independent Poisson spike train (Arenz et al., 2008; van Kan et al., 1993) and each spike triggered excitatory synaptic responses in connected GCs. GCs received a fixed number of MF synaptic inputs (depending on the network configuration) in the form of trains of AMPAR- and NMDAR-mediated conductances (Figures 7A and 7B), producing trains of EPSPs and spikes (Figure 7C).

Since the GC input-output (I-O) relationship is determined predominantly by the properties of the MF synaptic conductances, which include slow glutamate spillover-mediated components (DiGregorio et al., 2002) and short-term plasticity (STP) (Saviane and Silver, 2006), we converted existing experimental measurements of Poisson trains of synaptic currents to conductances (Rothman et al., 2009; Schwartz et al., 2012) and used them to constrain STP models of synaptic AMPAR and NMDAR components (Figures 7D and 7E, respectively). Moreover, we used an NMDAR model that captured the measured voltage dependence of NMDARs in GCs (Figure 7E, inset; Schwartz et al., 2012). Lastly, inhibition was implemented with a tonic $GABA_A$R-mediated inhibition, with a conductance of 438 pS and a reversal potential of −79.1 mV (Rothman et al., 2009; Seja et al., 2012; Figure 7A, green line). With these experimentally constrained settings, the I-O relationship of the model GC (Figure 7B) was similar to that observed in real GCs (Rothman et al., 2009; Schwartz et al., 2012). For networks with different numbers of synaptic inputs per GC the AMPAR- and NMDAR-mediated conductance amplitudes were scaled to conserve the total excitatory conductance (Supplemental Information). Biologically detailed networks were implemented using neuroConstruct (Gleeson et al., 2007) and simulated with NEURON (Carnevale and Hines, 2006).

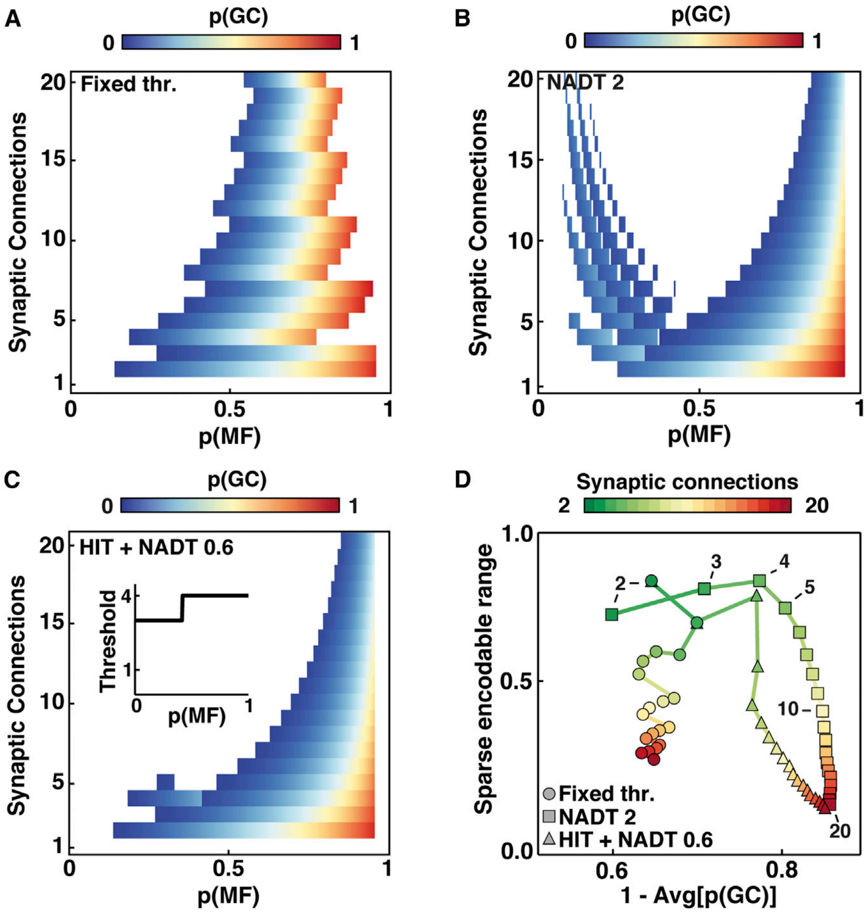

Figure 6. Networks that Best Perform Lossless Sparse Encoding Have Few Synaptic Connections per Neuron

(A) Granule cell activation probability (*p*(*GC*)) versus mossy fiber activation probability (*p*(*MF*)) with different numbers of synaptic connections per GC (*d*) for a fixed relative threshold (φ = *ceiling [0.75* × *d]*). Colored regions indicate sparse encodable range, where >99% of information was encoded and *p(GC)* < *p(MF)*.

(B and C) Same as for (A) but for network-activity-dependent threshold (NADT) = 2 and a high initial threshold (HIT) combined with NADT = 0.6, respectively. Inset in (C) shows threshold function for networks with *d* = 4.

(D) Relationship between size of sparse encodable range and output sparseness (1-Avg[*p(GC)*] averaged across all values of *p(MF)*). Color code indicates *d* and circle, square, and triangle symbols show different threshold functions in (A), (B), and (C), respectively.

Quantification of Transmission and Transformation of Information in Biologically Detailed Spiking Network Models

We quantified the I-O relationships of biologically detailed networks using sets of MF input patterns (Figures 7F, 7G, and 7H). Each input pattern was generated by randomly selecting a subset of the 176 MF inputs and designating them to be active (black lines in barcode, Figure 7F), while the remainder were inactive. During the simulation, active MFs fired random Poisson trains with a mean rate of 80 Hz and inactive MFs fired at 10 Hz (red raster plot in Figure 7F), reflecting the properties of real MF rate-coded inputs (Arenz et al., 2008; van Kan et al., 1993). This resulted in individual GCs receiving both high- and low-frequency trains of synaptic input conductances (e.g., top two and bottom two traces in Figure 7A, respectively). The output spiking of the 509 GCs in the network (blue spheres Figure 7G) was recorded over a 30 ms time window, corresponding to the synaptic integration time of GCs (Figure S4B; Schwartz et al., 2012). For each MF input pattern, the number of spikes was calculated for each GC, and this was expressed as a vector for the GC population (blue barcode in Figure 7H). To quantify network performance, we used sets of N = 1,024 MF input patterns, which was the largest number achievable with the computational resources available.

Since direct calculation of the Shannon information between input and output spike trains was computationally intractable, we reduced the dimensionality of the output space by cascading the network with a classifier (called the decoder), which labeled the output spike count vectors as belonging to one of N classes (Experimental Procedures). The network and decoder constituted a communication channel that mapped N input patterns to N output classes, for which we calculated the mutual information (MI) assuming a flat prior over inputs. This fixed the maximum MI achievable as $\log_2(1,024)$ = 10 bits. Unlike for the UBN model, Shannon information in the biologically detailed model does not correspond to the GC population entropy because of the noise introduced by the random spike trains. The network transformation was quantified by calculating the GC population sparseness (Vinje and Gallant, 2000). This measure is analogous to 1-*p(GC)* for the binary case, enabling comparison of the binary and spiking models. The average output sparseness provided a single measure of GC population sparseness across *p(MF)*.

Best-Performing Synaptic Connectivity for Lossless Sparse Encoding in Biologically Detailed Spiking Network Models

We first examined how effectively spiking network models with different synaptic connectivity transmitted independent MF patterns (Figure 8A). Networks with few synaptic connections per neuron were most effective at transmitting information across the widest range of MF input activity, defined as the fraction of MF inputs active (*p(MF)*) (Figure 8B). Indeed, networks with few inputs recovered almost all of the maximum of ten bits, which is remarkable, given the noisy nature of the encoding and that GC spikes were only decoded over 30 ms. However, for

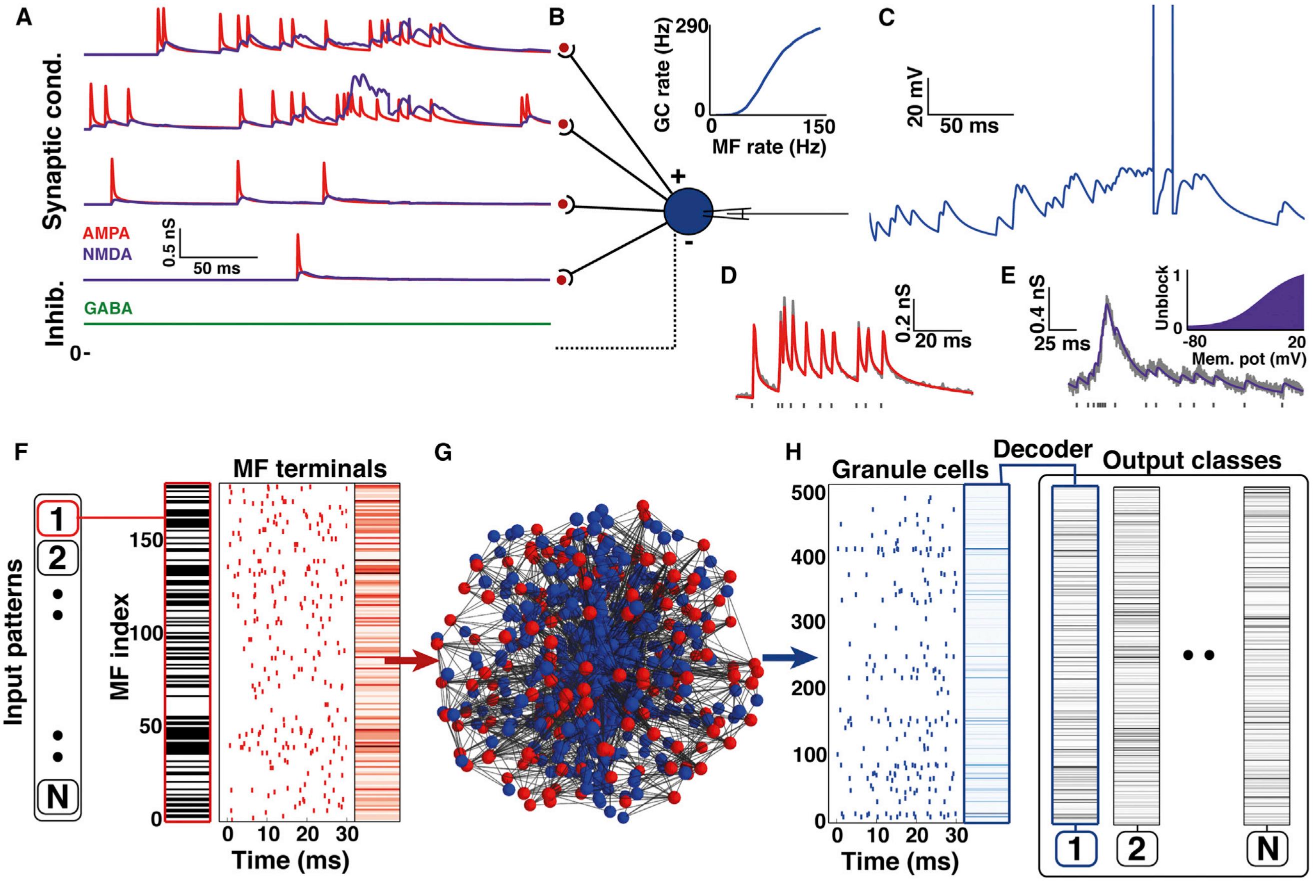

Figure 7. Construction and Analysis of an Experimentally Constrained Spiking Model of the Local Granule Cell Layer Network Incorporating Synaptic Mechanisms and Tonic Inhibition

(A) Excitatory AMPAR (red) and NMDAR (purple) synaptic conductances for four independent mossy fiber (MF) inputs injected into a model granule cell (GC). Top two traces: active MFs with excitatory conductance driven by independent Poisson spike trains firing at 80 Hz. Lower two traces: inactive MF firing at 10 Hz. Bottom trace: tonic inhibitory $GABA_AR$ conductance (green).

(B) Model GC with action potential firing rate-coded input-output relationship (above) for four synaptic inputs.

(C) Membrane potential of model GC during synaptic input in (A).

(D) Fit of the short-term plasticity model (red) of the AMPAR component to an experimental recording of a 100 Hz synaptic conductance train (gray).

(E) Same as for (D) but for the NMDAR component and an 80 Hz conductance train. Inset: voltage dependence of NMDAR conductance.

(F) A binary stimulus pattern was randomly selected from a set of N patterns (black active and white inactive on barcode). A Poisson spike train was generated for each MF input (80 Hz active, 10 Hz inactive; red raster plot), thereby setting the timing of synaptic conductances (as in A). Red barcode indicates spike counts for the given realization of the spike trains.

(G) 3D view of the anatomically constrained local GCL network model with 176 MFs in red and 509 GCs in blue.

(H) Raster plot of GC firing activity in response to the input. Blue barcode indicates GC spike count vector (measured over a 30 ms window), which was assigned to one of N output classes (black bar codes) defined using the k-means algorithm on a separate data set.

networks with larger numbers of synaptic connections per neuron information transmission performance decreased across large regions of *p(MF)*. Reducing or extending the window over which spikes were decoded or altering the firing rates of active MFs shifted the dependence of MI on connectivity, but the overall relationship remained the same (Figures S4 and S5). These results show that biologically detailed spiking networks with few synaptic connections per neuron are most effective at transmitting information, as predicted from the UBN model.

The sparseness of the GC population, averaged over *p(MF)*, increased as the number of MF inputs per GC increased (Figure 8C). Thus, the network connectivity-dependent trade-off between information transmission and sparsification is also present in spiking models. This arose because as the number of synaptic connections per GC increased, the network I-O relationship became highly nonlinear, reducing the average number of spikes per GC to levels well below the average numbers of spikes per MF (Figure 8G). Although encoding in this region was sparse, so few GCs were activated that information transmission was compromised. To find the best-performing biological network configurations, we plotted the relationship between the average fraction of information recovered by the GCs versus average sparsification performed by each network (Figure 8H). Networks with two to seven synaptic

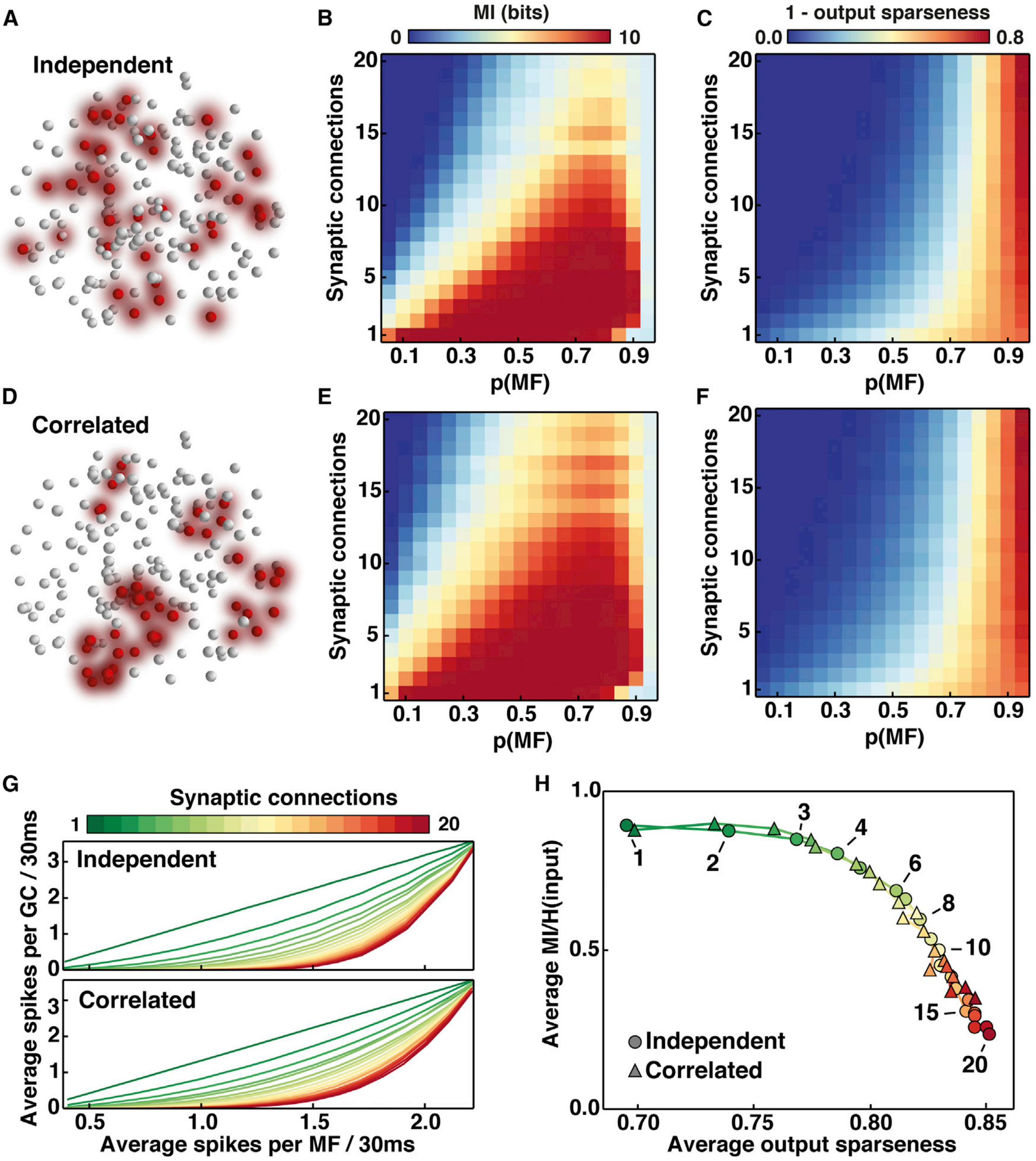

Figure 8. Sparse Encoding in Biologically Detailed Spiking Network Models with Different Numbers of Synaptic Connections per Granule Cell

(A) Visualization of independent mossy fiber (MF) inputs in the local granule cell (GC) layer network model with active MFs in red and inactive MFs in white, for an example random activation pattern.

(B) Mutual information (MI) encoded by the GC population for 1,024 uncorrelated input patterns across the full range of MF activation probability *p*(*MF*) in biologically detailed spiking networks with different numbers of synaptic connections per GC (*d*).

(C) Same as for (B) but for 1-average output sparseness (analogous to *p(GC)* in UBN model).

(D, E, and F) Same as for (A), (B), and (C) but for a set of 1,024 spatially correlated patterns, where neighboring MF inputs were activated in groups of five.

(G) Same as for (B) and (E) but visualized as a line plot to show the relationship between average spikes per GC and average spikes per MF in a 30 ms window across all values of *p(MF)*, for networks with different *d*.

(H) Relationship between average MI (normalized by the MF input entropy) and average output sparseness (across all values of *p(MF)*) for spiking networks with different *d* (color code) for independent (circles) and spatially correlated (triangles) inputs.

connections per GC provided the best solution for performing sparse lossless encoding, with four inputs performing particularly well, supporting the predictions from our simplified analytical approach.

Spatial correlation in MF input activity is likely to occur in real cerebellar networks and may vary from region to region, due to variations in the numbers of MFs arising from different origins (Huang et al., 2013). Since one of the central assumptions in

our simplified analytical approach was that all MF inputs were independent, we examined how spatial correlations in the MF activity patterns affected information transmission and sparsification in spiking networks. Introduction of a pronounced spatial correlation in the input, where groups of five neighboring MFs are activated (Figure 8D), marginally increased the information transmission performance over all networks (c.f. Figures 8B and 8E). This is due to GCs in a particular region receiving a larger fraction of active MF inputs and thus having a greater chance of generating a spike. Output sparseness of the GC population was little affected by spatial correlations in the MF input activity (cf. Figures 8C and 8F). Thus, spatial correlations in MF activity only subtly shift the trade-off between information transmission and sparsification in biologically detailed spiking networks.

Finally, we tested whether adding network-activity-dependent inhibition to the physiological level of tonic inhibition improved performance of our spiking networks, as predicted from the UBN model. To do this, we scaled the tonic inhibitory conductance as a function of *p(MF)*, from the experimentally measured tonic level. This reduced information transmission in networks with larger numbers of synaptic connections (Figure S6A). However, for networks with few synaptic connections per neuron, information transmission was preserved and GC activity was further sparsened. Biologically detailed networks with three to five synaptic connections, tonic inhibition, and a modest network-activity-dependent inhibition (NADT = 0.3) performed lossless sparse encoding better than tonic inhibition alone (Figure S6C), again supporting the predictions of our binary model. Interestingly, all three of these features are characteristic properties of the cerebellar GCL, suggesting that both the connectivity and the inhibition properties are tuned to enable robust lossless sparse encoding over the widest range of MF excitatory drive.

DISCUSSION

We have explored the relationship between the structure of feedforward networks and their ability to transmit information and transform it into a sparse representation, which are both essential for pattern separation. By combining quantitative anatomy of the cerebellar input layer and a full information theoretic treatment of uniform binary network models, we show that the extent of the synaptic connectivity in feedforward networks sets the trade-off between information transmission and sparse encoding. Networks with two to seven synaptic connections per output neuron perform lossless sparse coding over the widest range of input activity. Structurally and functionally detailed spiking network models with synaptic inputs, neuronal properties, and tonic inhibition constrained to experimentally measured values confirmed that the properties of the cerebellar input layer are particularly well suited for performing lossless sparse encoding. Our results therefore provide a computational explanation of why the most numerous neuron in the brain of vertebrates receives an average of four excitatory synaptic inputs.

The Relationship between Feedforward Network Structure and Function

To understand how synaptic connectivity affects network function, it is first necessary to understand why information transmission and sparse encoding are competing functions. Although a local network of 500 GCs could potentially encode an astronomical number of MF input patterns (i.e., 2^{500} for a binary network), when GC activity is reduced to low levels the capacity of the network to encode patterns shrinks considerably. Our results show that the balance between information transmission and the sparseness of the encoding is set by the extent of synaptic connectivity. Increasing the number of synaptic inputs required to reach firing threshold provides a sparser output representation, but if the probability of GC activation becomes too low, GCL encoding capacity falls and information is lost. By contrast, if the GC network capacity is much larger than the number of MF patterns to be encoded, the GC population activity could be reduced to a sparser representation, thereby saving energy (Attwell and Laughlin, 2001) and improving pattern separation (Tyrrell and Willshaw, 1992).

Results from our biologically constrained spiking networks confirmed that few excitatory synaptic connections per neuron and a high level of inhibition provide a highly effective trade-off between information transmission and sparse encoding. The fact that the synaptic connectivity of the best performing networks match that found in the cerebellar input layer suggests that the GCL structure is optimized for transforming MF input patterns into a higher dimensional sparser code, without information loss. Our results extend classical work on the relationship between cerebellar structure and function (Albus, 1971; Kanerva, 1988; Marr, 1969), by showing that the synaptic connectivity between MFs and GCs is a major determinant of information transmission and sparse encoding in this brain region.

Relationship between Mossy Fiber Activity and Granule Cell Layer Properties

Since the cerebellum receives dynamic patterns of sensory-motor inputs via the MF system, the ability to perform lossless sparse encoding over a wide range of MF excitatory drive is likely to be crucial. Indeed, in vivo recordings show that MFs exhibit a wide range of activity, with those signaling rapid discrete sensory events exhibiting high-frequency bursts and relatively quiescent periods (Rancz et al., 2007), while those that convey slower continuous sensory variables, such as joint angle and head velocity, typically fire continuously at 10–100 Hz (Arenz et al., 2008; van Kan et al., 1993). The spatial patterns of MF activation are also likely to be highly diverse. Although MF innervations of the GCL exhibit a large-scale fractured map topology (Shambes et al., 1978), the MFs that innervate an individual GC typically arise from different precerebellar nuclei (Huang et al., 2013), suggesting that MF activation is rather spatially independent and that a key function of the GCL is to combine information from different modalities. However, MF inputs onto individual GCs in vermal areas that encode limb movement carry highly correlated information (Jörntell and Ekerot, 2006). Our results show that the synaptic connectivity found within the GCL can losslessly transform a wide range of MF excitatory drive into a sparse GC population code, even when spatial correlations in MF activity are present. Moreover, the broad bandwidth of MF-GC signaling (Saviane and Silver, 2006) also enables certain sensory stimuli, such as whisker deflection, which generate high-frequency MF bursts (e.g., 700 Hz), to be relayed through the GCL (Rancz

et al., 2007). Thus, our results suggest that the GCL acts as a general purpose sparse encoder of rate-coded MF inputs that has the flexibility to respond rapidly to urgent stimuli.

Determinants of Network Encoding Capacity

Our results show that the encoding capacity of noise-free binary networks is sufficiently large to encode all patterns that an animal could possibly encounter during its lifetime. On the other hand, we show that noisy biologically detailed spiking networks can comfortably encode 1,024 rate-coded MF input patterns, assuming GC spikes are integrated over 30 ms. While the number of patterns that a real local GCL network encodes falls between these two values, it may vary widely across cerebellar regions because encoding capacity depends on MF firing rates (Figure S5), correlations in MF activity, the properties of inhibition (Figure S6), and the time window over which GC firing is integrated (Figure S4). These considerations suggest that the encoding capacity of a local GCL network will depend strongly on the properties of the MF inputs it receives.

Another way to increase the encoding capacity is to increase the number of local networks engaged. In vivo recordings from GCs in mouse vestibular cerebellum indicate that ~400 MF-GC synapses are required to encode head velocity at the precision observed in man (Arenz et al., 2008), suggesting that multiple local GCL networks are involved. Indeed, MF axons, which form ~20 en passant synaptic rosettes (Eccles et al., 1967; Sultan, 2001), enable GCs in neighboring local networks to sample the same MF signals. The idea that many GCs are required for sensory representations is supported by the finding that markedly reducing the number of functional GCs induces deficits in consolidation of motor learning (but concomitant changes in long-term plasticity could also contribute to these effects; Galliano et al., 2013). These observations are consistent with the notion that multiple local GCL networks are involved in certain sensory-motor tasks and that the large MF to GC divergence found in the cerebellum is required for efficient encoding.

The Properties of Inhibition and Encoding Capacity

Our results show that the physiological level of tonic $GABA_A$R-mediated inhibition (Brickley et al., 1996) provides a robust solution for performing lossless sparse encoding. Recent in vivo recordings show that tonic inhibition dominates other forms of inhibition in GCs, accounting for 98% of the inhibitory charge (Duguid et al., 2012). This sets a relatively high threshold so that simultaneous activity from three or more rate-coded MF inputs are typically required to reach GC firing threshold (Jörntell and Ekerot, 2006; Schwartz et al., 2012). The importance of a high GC spike threshold to cerebellar function is reinforced by the finding that when tonic inhibition was eliminated in $GABA_A\alpha_6$ knockout mice, two-pore K^+ channels were upregulated, thereby maintaining threshold at a high level (Brickley et al., 2001). However, knocking out transporters has been more effective in modulating GC threshold. Deletion of the GABA transporter GAT1 increased tonic inhibition in GCs by 4-fold and was associated with tremor and ataxia (Chiu et al., 2005). Our results suggest that information loss could have contributed to these behavioral effects. Lowering GC spike threshold by selectively deleting the KCC2 chloride transporter in GCs, which our results would suggest reduces the sparseness of encoding and thus pattern separation, impairs learning consolidation (Seja et al., 2012). Thus, our models of GCL function provide insights into how alterations in the level of tonic inhibition could impair cerebellar function and why GC spike threshold is tightly regulated by homeostatic mechanisms.

When network-activity-dependent inhibition was added to tonic inhibition, it further sparsified GC encoding without loss of information. Our results therefore support previous proposals that Golgi cells aid sparse coding by controlling the gain to the GCL (Albus, 1971; Marr, 1969; Schweighofer et al., 2001). Although weaker, the phasic and spillover components of Golgi cell-mediated inhibition (Rossi et al., 2003) may also contribute to temporal pattering, which could perform temporal sparsening of GC spikes, time slicing (D'Angelo and De Zeeuw, 2009) and introduce delays that are important for learning temporal operations such as eyeblink conditioning (Medina and Mauk, 2000) and signal cancellation (Kennedy et al., 2014). However, the longer temporal delays during signal cancellation are mediated by unipolar brush cells (Kennedy et al., 2014), which form short-range intrinsic MFs that could increase spatial correlations in the vestibular cerebellum where they are more numerous. Indeed, regional variations in the origin of MF inputs, the presence of UBCs and synaptic plasticity within the MF-GC-Golgi cell circuit could tune the spatiotemporal transformation that specific GCL "modules" perform. Our results show that few synaptic connections per GCs provide a robust structural framework that enables a wide range of MF activity patterns to be transmitted and sparsified efficiently.

Synaptic Connectivity of the Cerebellar Input Layer Is Evolutionarily Conserved

The cerebellum is an ancient brain structure that arose in the early vertebrates. In terms of numbers, cerebellar GCs dominate the vertebrate CNS, making up more than half of all the neurons in the human brain (Williams and Herrup, 1988). Remarkably, the morphology of cerebellar GCs is conserved across a wide range of species including fish, amphibians, reptiles, and mammals (Llinás, 1969; Wittenberg and Wang, 2007), demonstrating that it has been evolutionarily conserved. In mammals, the observed range is two to seven dendrites (and thus MF inputs) per GC (Palkovits et al., 1972) with four per cell being the most common configuration. The strikingly similarity between the synaptic connectivity in the cerebellar GCL and the feedforward networks that provide the best trade-off between information transmission and sparsification provides a functional explanation for why the characteristic dendritic morphology of cerebellar GCs has been conserved for hundreds of millions of years.

Comparison of the Structure of the GCL to Other Networks

The cerebellar GCL is not the only example of a network that performs sparsification and has few synaptic connections per neuron. GCs in the dorsal cochlear nucleus and deep GC in the electrosensory lobe (ELL) of the electric fish have one to four synaptic inputs, averaging to three in the ELL (Kennedy

et al., 2014; Mugnaini et al., 1980; Zhang et al., 2007). Kenyon cells in the mushroom body of the fly receive an average of seven synaptic inputs from olfactory projection neurons (Caron et al., 2013). Indeed, expansion recoding in the insect mushroom body has other similarities to the cerebellar input layer (Laurent, 2002), including random connectivity (Caron et al., 2013), and inhibitory interneurons that facilitate sparsification (Papadopoulou et al., 2011), enhancing pattern separation and enabling the discrimination of similar odors (Lin et al., 2014). These examples suggest that other brain structures may have converged on a similar feedforward network structure for performing lossless sparse encoding.

If few synaptic connections provide an evolutionary advantage, then why don't other input layers exhibit a similar structure to the cerebellar GCL? Although we cannot provide a definitive answer, the distinct functions performed by different brain regions provide some hints. Spiny stellate cells in layer 4 of neocortex receive many more synaptic inputs than GCs, but these are predominantly recurrent excitatory connections, which amplify thalamic synaptic input that display strong short-term depression (Lien and Scanziani, 2013). Moreover, nonlinear NMDAR spikes in the dendrites of spiny stellate cells also amplify synaptic inputs (Lavzin et al., 2012). Recurrent connections introduce loops that can support attractor states, intrinsic activity, and complex nonlinear dynamics (Buonomano and Maass, 2009) and aid receptive field formation and feature extraction (Somers et al., 1995). These synaptic, cellular, and network properties appear tuned to detect features and amplify novel stimuli, producing gradually fading memory traces that enable sensory input to be combined with recent experience (Buonomano and Maass, 2009). These sophisticated operations may explain why the structure of the neocortical input layer is more complex than cerebellar input layer, which lacks recurrent excitatory connections.

Experimentally Testable Predictions

Our results make a number of predictions that could potentially be tested experimentally: (1) GC population activity is sparser than sustained MF activity within a local region; (2) information is conserved within local GCL networks; (3) reduction of tonic inhibition impairs pattern separation, while elevation impairs information transmission; (4) network-activity-dependent inhibition improves lossless sparse encoding; and (5) increasing the number of MFs inputs per GC aids sparsification but impairs information transmission. However, experimental validation is complicated by the need to measure spike trains from local populations of MFs and GC in awake behaving animals and potential compensatory effects associated with genetic changes. Nevertheless, work combining genetic manipulations and motor learning consolidation (Galliano et al., 2013; Seja et al., 2012) and developments in fast 3D imaging technologies (Fernández-Alfonso et al., 2014) look encouraging.

EXPERIMENTAL PROCEDURES

Measurement of Granule Cell and Glomerular Density

Four 30-day-old Sprague-Dawley rats were deeply anesthetized and perfused with 4% paraformaldehyde in 0.1 M phosphate buffer and 40-μm-thick sagittal sections of cerebellum were prepared. Cerebellar glomeruli were labeled with anti-Kv4.2, anti-VGAT and anti-VGLUT1, or anti-GLAST primary antibodies and Alexa 488, Cy5, or CY3 labeled secondary antibodies (Supplemental Information) and visualized with a confocal scanning microscope. The two-way dissector method was used to determine GC density within GCL and tissue shrinkage was taken into account. Density of glomeruli was calculated from the mean volume of glomeruli and the mean volume of GCL occupied by glomeruli.

Construction of the Uniform Binary Network Model

The UBN model was constructed with connectivity statistics as close as possible to the anatomically constrained local GCL network model. The UBN model is formally equivalent to a random bipartite graph consisting of two disjoint sets of nodes (inputs, representing MF rosettes, and outputs, representing GCs), with each output node connected to a fixed number of randomly chosen input nodes (representing the number of synaptic connections per GC). The relative fixed binary threshold was implemented across networks with different numbers of synaptic connections per GC (d) by setting the minimum GC threshold to *ceiling[0.75* × *d]*, where ceiling rounds noninteger values up to the nearest integer. NADT was modeled as a dependence between the threshold of the GCs and $p(MF)$, using a piecewise constant function, monotonically increasing from 1 to d over a fraction of the total $p(MF)$ range given by 1/NADT (e.g., raising from 1 to d between $p(MF) = 0$ and $p(MF) = 1/2$ for NADT = 2).

Information Theoretical Analysis of the Uniform Binary Network Model

Because the UBN model is noise-free the Shannon Information between the events and the state of the GC population is the GC population entropy (H),

$$H = -\sum_{k=1}^{G} p(k)\log_2[p(k)] \tag{1}$$

where $p(k)$ is the probability of the kth of G unique GC patterns caused by events. We developed a mathematical technique that enabled us to directly calculate average entropy across network instantiations (see derivation of Equation 29; Appendix, Supplemental Information) even for large numbers of GC patterns. Computation time using our analytical method is independent of the number of events.

Biologically Detailed Spiking Network Simulations

For spiking networks, we used the anatomically constrained local GCL network model. GCs were modeled using a conductance-based integrate-and-fire model whose parameters were set to previously published experimental averages (Rothman et al., 2009; Schwartz et al., 2012; Table S3). AMPAR- and NMDAR-mediated synaptic conductances were fitted using swarm intelligence techniques (Supplemental Information) to measured EPSCs (Rothman et al., 2009), with short-term plasticity modeled as in Tsodyks et al. (1998). We used 1,024 MF patterns, since this was the maximum possible with the computational resources available: simulations and analysis of Figures 8, S5, and S6 required more than one million 2 GHz core hours. Cell and synaptic models in NeuroML2/LEMS format (Gleeson et al., 2010) and links to the simulation and data management code are available on the Open Source Brain (http://www.opensourcebrain.org/projects/granule-cell-layer-piasini-2014).

Analysis of Spiking Network Data

Mutual information was calculated between the set of N input patterns and N output network activity classes, obtained by performing an appropriate tessellation of the output space, as this was the smallest number of classes that allowed for full recovery of information (Supplemental Information). Undersampling bias in the MI estimate (Treves and Panzeri, 1995) was accounted and corrected for. Population sparseness was defined as

$$S = \left(C - \frac{\left(\sum_{i=1}^{c} r_i\right)^2}{\sum_{i=1}^{c} r_i^2}\right) \Big/ (C-1)$$

(Vinje and Gallant, 2000) where C is the number of cells and r_i is the spike count of cell i.

SUPPLEMENTAL INFORMATION

Supplemental Information includes Supplemental Experimental Procedures, six figures, and two tables and can be found with this article online at http://dx.doi.org/10.1016/j.neuron.2014.07.020.

AUTHOR CONTRIBUTIONS

G.B. developed the UBN model and analytical methods, with refinements from E.P. and R.A.S. E.P. built the spiking model and software to manage the numerical simulations and performed analysis. A.L. and Z.N. performed experiments and the quantitative anatomical analysis. R.A.S. conceived and supervised the project and wrote the manuscript, with contributions from all authors.

ACKNOWLEDGMENTS

Support was provided to R.A.S. from the BBSRC (F005490), Wellcome Trust (086699), and his ERC Advanced Grant (294667) and Wellcome Trust Principal Research Fellowship (095667); to E.P. from the EU Marie Curie Initial Training Network CEREBNET (FP7-ITN-PEOPLE-2008; 238686); to Z.N. from the Wellcome Trust (WT094513), a Lendület Grant from the Hungarian Academy of Sciences (LP2012-29), and his ERC Advanced Grant; and to A.L. from a Janos Bolyai Scholarship of the Hungarian Academy of Sciences. We thank Laszlo Kocsis for help with analyzing glomeruli volume and D. Attwell, P. Dayan, M. van Rossum, C. Houghton, J. Rothman, A. Valera, and B. Marin for comments on the manuscript. We acknowledge use of the UCL Computer Science Cluster and Legion HPC Facility (Legion@UCL) and support services.

Accepted: June 25, 2014
Published: August 7, 2014

REFERENCES

Albus, J.S. (1971). A theory of cerebellar function. Math. Biosci. *10*, 25–61.

Arenz, A., Silver, R.A., Schaefer, A.T., and Margrie, T.W. (2008). The contribution of single synapses to sensory representation in vivo. Science *321*, 977–980.

Attwell, D., and Laughlin, S.B. (2001). An energy budget for signaling in the grey matter of the brain. J. Cereb. Blood Flow Metab. *21*, 1133–1145.

Brickley, S.G., Cull-Candy, S.G., and Farrant, M. (1996). Development of a tonic form of synaptic inhibition in rat cerebellar granule cells resulting from persistent activation of GABAA receptors. J. Physiol. *497*, 753–759.

Brickley, S.G., Revilla, V., Cull-Candy, S.G., Wisden, W., and Farrant, M. (2001). Adaptive regulation of neuronal excitability by a voltage-independent potassium conductance. Nature *409*, 88–92.

Briggman, K.L., Helmstaedter, M., and Denk, W. (2011). Wiring specificity in the direction-selectivity circuit of the retina. Nature *471*, 183–188.

Buonomano, D.V., and Maass, W. (2009). State-dependent computations: spatiotemporal processing in cortical networks. Nat. Rev. Neurosci. *10*, 113–125.

Carnevale, N.T., and Hines, M.L. (2006). The NEURON Book. (Cambridge: Cambridge University Press).

Caron, S.J.C., Ruta, V., Abbott, L.F., and Axel, R. (2013). Random convergence of olfactory inputs in the Drosophila mushroom body. Nature *497*, 113–117.

Cesana, E., Pietrajtis, K., Bidoret, C., Isope, P., D'Angelo, E., Dieudonné, S., and Forti, L. (2013). Granule cell ascending axon excitatory synapses onto Golgi cells implement a potent feedback circuit in the cerebellar granular layer. J. Neurosci. *33*, 12430–12446.

Chiu, C.-S., Brickley, S., Jensen, K., Southwell, A., Mckinney, S., Cull-Candy, S., Mody, I., and Lester, H.A. (2005). GABA transporter deficiency causes tremor, ataxia, nervousness, and increased GABA-induced tonic conductance in cerebellum. J. Neurosci. *25*, 3234–3245.

D'Angelo, E., and De Zeeuw, C.I. (2009). Timing and plasticity in the cerebellum: focus on the granular layer. Trends Neurosci. *32*, 30–40.

DiGregorio, D.A., Nusser, Z., and Silver, R.A. (2002). Spillover of glutamate onto synaptic AMPA receptors enhances fast transmission at a cerebellar synapse. Neuron *35*, 521–533.

Duguid, I., Branco, T., London, M., Chadderton, P., and Häusser, M. (2012). Tonic inhibition enhances fidelity of sensory information transmission in the cerebellar cortex. J. Neurosci. *32*, 11132–11143.

Dyhrfjeld-Johnsen, J., Santhakumar, V., Morgan, R.J., Huerta, R., Tsimring, L., and Soltesz, I. (2007). Topological determinants of epileptogenesis in large-scale structural and functional models of the dentate gyrus derived from experimental data. J. Neurophysiol. *97*, 1566–1587.

Eccles, J.C., Ito, M., and Szentágothai, J. (1967). The Cerebellum as a Neuronal Machine. (New York: Springer-Verlag).

Fernández-Alfonso, T., Nadella, K.M.N.S., Iacaruso, M.F., Pichler, B., Roš, H., Kirkby, P.A., and Silver, R.A. (2014). Monitoring synaptic and neuronal activity in 3D with synthetic and genetic indicators using a compact acousto-optic lens two-photon microscope. J. Neurosci. Methods *222*, 69–81.

Fujita, M. (1982). Adaptive filter model of the cerebellum. Biol. Cybern. *45*, 195–206.

Galliano, E., Gao, Z., Schonewille, M., Todorov, B., Simons, E., Pop, A.S., D'Angelo, E., van den Maagdenberg, A.M., Hoebeek, F.E., and De Zeeuw, C.I. (2013). Silencing the majority of cerebellar granule cells uncovers their essential role in motor learning and consolidation. Cell Rep. *3*, 1239–1251.

Gleeson, P., Steuber, V., and Silver, R.A. (2007). neuroConstruct: a tool for modeling networks of neurons in 3D space. Neuron *54*, 219–235.

Gleeson, P., Crook, S., Cannon, R.C., Hines, M.L., Billings, G.O., Farinella, M., Morse, T.M., Davison, A.P., Ray, S., Bhalla, U.S., et al. (2010). NeuroML: a language for describing data driven models of neurons and networks with a high degree of biological detail. PLoS Comput. Biol. *6*, e1000815.

Harvey, R.J., and Napper, R.M. (1988). Quantitative study of granule and Purkinje cells in the cerebellar cortex of the rat. J. Comp. Neurol. *274*, 151–157.

Honey, C.J., Kötter, R., Breakspear, M., and Sporns, O. (2007). Network structure of cerebral cortex shapes functional connectivity on multiple time scales. Proc. Natl. Acad. Sci. USA *104*, 10240–10245.

Huang, C.-C., Sugino, K., Shima, Y., Guo, C., Bai, S., Mensh, B.D., Nelson, S.B., and Hantman, A.W. (2013). Convergence of pontine and proprioceptive streams onto multimodal cerebellar granule cells. Elife (Cambridge) *2*, e00400.

Jörntell, H., and Ekerot, C.-F. (2006). Properties of somatosensory synaptic integration in cerebellar granule cells in vivo. J. Neurosci. *26*, 11786–11797.

Kanerva, P. (1988). Sparse Distributed Memory. (Cambridge: The MIT Press).

Kanichay, R.T., and Silver, R.A. (2008). Synaptic and cellular properties of the feedforward inhibitory circuit within the input layer of the cerebellar cortex. J. Neurosci. *28*, 8955–8967.

Kennedy, A., Wayne, G., Kaifosh, P., Alviña, K., Abbott, L.F., and Sawtell, N.B. (2014). A temporal basis for predicting the sensory consequences of motor commands in an electric fish. Nat. Neurosci. *17*, 416–422.

Ko, H., Hofer, S.B., Pichler, B., Buchanan, K.A., Sjöström, P.J., and Mrsic-Flogel, T.D. (2011). Functional specificity of local synaptic connections in neocortical networks. Nature *473*, 87–91.

Laurent, G. (2002). Olfactory network dynamics and the coding of multidimensional signals. Nat. Rev. Neurosci. *3*, 884–895.

Lavzin, M., Rapoport, S., Polsky, A., Garion, L., and Schiller, J. (2012). Nonlinear dendritic processing determines angular tuning of barrel cortex neurons in vivo. Nature *490*, 397–401.

Lien, A.D., and Scanziani, M. (2013). Tuned thalamic excitation is amplified by visual cortical circuits. Nat. Neurosci. *16*, 1315–1323.

Lin, A.C., Bygrave, A.M., de Calignon, A., Lee, T., and Miesenböck, G. (2014). Sparse, decorrelated odor coding in the mushroom body enhances learned odor discrimination. Nat. Neurosci. *17*, 559–568.

Livet, J., Weissman, T.A., Kang, H., Draft, R.W., Lu, J., Bennis, R.A., Sanes, J.R., and Lichtman, J.W. (2007). Transgenic strategies for combinatorial expression of fluorescent proteins in the nervous system. Nature *450*, 56–62.

Llinás, R. (1969). Neurobiology of Cerebellar Evolution and Development. (Chicago: American Medical Association).

Marr, D. (1969). A theory of cerebellar cortex. J. Physiol. *202*, 437–470.

Medina, J.F., and Mauk, M.D. (2000). Computer simulation of cerebellar information processing. Nat. Neurosci. *3* (*Suppl*), 1205–1211.

Mugnaini, E., Osen, K.K., Dahl, A.L., Friedrich, V.L., Jr., and Korte, G. (1980). Fine structure of granule cells and related interneurons (termed Golgi cells) in the cochlear nuclear complex of cat, rat and mouse. J. Neurocytol. *9*, 537–570.

Olshausen, B.A., and Field, D.J. (2004). Sparse coding of sensory inputs. Curr. Opin. Neurobiol. *14*, 481–487.

Palay, S.L., and Chan-Palay, V. (1974). Cerebellar Cortex: Cytology and Organisation. (Berlin: Springer).

Palkovits, M., Magyar, P., and Szentágothai, J. (1972). Quantitative histological analysis of the cerebellar cortex in the cat. IV. Mossy fiber-Purkinje cell numerical transfer. Brain Res. *45*, 15–29.

Papadopoulou, M., Cassenaer, S., Nowotny, T., and Laurent, G. (2011). Normalization for sparse encoding of odors by a wide-field interneuron. Science *332*, 721–725.

Rancz, E.A., Ishikawa, T., Duguid, I., Chadderton, P., Mahon, S., and Häusser, M. (2007). High-fidelity transmission of sensory information by single cerebellar mossy fibre boutons. Nature *450*, 1245–1248.

Rossi, D.J., Hamann, M., and Attwell, D. (2003). Multiple modes of GABAergic inhibition of rat cerebellar granule cells. J. Physiol. *548*, 97–110.

Rothman, J.S., Cathala, L., Steuber, V., and Silver, R.A. (2009). Synaptic depression enables neuronal gain control. Nature *457*, 1015–1018.

Saviane, C., and Silver, R.A. (2006). Fast vesicle reloading and a large pool sustain high bandwidth transmission at a central synapse. Nature *439*, 983–987.

Schwartz, E.J., Rothman, J.S., Dugué, G.P., Diana, M., Rousseau, C., Silver, R.A., and Dieudonné, S. (2012). NMDA receptors with incomplete Mg^{2+} block enable low-frequency transmission through the cerebellar cortex. J. Neurosci. *32*, 6878–6893.

Schweighofer, N., Doya, K., and Lay, F. (2001). Unsupervised learning of granule cell sparse codes enhances cerebellar adaptive control. Neuroscience *103*, 35–50.

Seja, P., Schonewille, M., Spitzmaul, G., Badura, A., Klein, I., Rudhard, Y., Wisden, W., Hübner, C.A., De Zeeuw, C.I., and Jentsch, T.J. (2012). Raising cytosolic Cl- in cerebellar granule cells affects their excitability and vestibulo-ocular learning. EMBO J. *31*, 1217–1230.

Shambes, G.M., Gibson, J.M., and Welker, W. (1978). Fractured somatotopy in granule cell tactile areas of rat cerebellar hemispheres revealed by micromapping. Brain Behav. Evol. *15*, 94–140.

Shannon, C.E. (1948). A mathematical theory of communication. Bell Syst. Tech. J. *27*, 379.

Silver, R.A., Traynelis, S.F., and Cull-Candy, S.G. (1992). Rapid-time-course miniature and evoked excitatory currents at cerebellar synapses in situ. Nature *355*, 163–166.

Somers, D.C., Nelson, S.B., and Sur, M. (1995). An emergent model of orientation selectivity in cat visual cortical simple cells. J. Neurosci. *15*, 5448–5465.

Sultan, F. (2001). Distribution of mossy fibre rosettes in the cerebellum of cat and mice: evidence for a parasagittal organization at the single fibre level. Eur. J. Neurosci. *13*, 2123–2130.

Talpalar, A.E., Bouvier, J., Borgius, L., Fortin, G., Pierani, A., and Kiehn, O. (2013). Dual-mode operation of neuronal networks involved in left-right alternation. Nature *500*, 85–88.

Treves, A., and Panzeri, S. (1995). The upward bias in measures of information derived from limited data samples. Neural Comput. *7*, 399–407.

Tsodyks, M., Pawelzik, K., and Markram, H. (1998). Neural networks with dynamic synapses. Neural Comput. *10*, 821–835.

Tyrrell, T., and Willshaw, D. (1992). Cerebellar cortex: its simulation and the relevance of Marr's theory. Philos. Trans. R. Soc. Lond. B Biol. Sci. *336*, 239–257.

van Beugen, B.J., Gao, Z., Boele, H.J., Hoebeek, F., and De Zeeuw, C.I. (2013). High frequency burst firing of granule cells ensures transmission at the parallel fiber to purkinje cell synapse at the cost of temporal coding. Front Neural Circuits *7*, 95.

van Kan, P.L., Gibson, A.R., and Houk, J.C. (1993). Movement-related inputs to intermediate cerebellum of the monkey. J. Neurophysiol. *69*, 74–94.

Vinje, W.E., and Gallant, J.L. (2000). Sparse coding and decorrelation in primary visual cortex during natural vision. Science *287*, 1273–1276.

Williams, R.W., and Herrup, K. (1988). The control of neuron number. Annu. Rev. Neurosci. *11*, 423–453.

Wittenberg, G.M., and Wang, S.S.-H. (2007). Evolution and scaling of dendrites. In Dendrites, G. Stuart, N. Spruston, and M. Hausser, eds. (New York: Oxford University Press), pp. 43–67.

Zhang, J., Han, V.Z., Meek, J., and Bell, C.C. (2007). Granular cells of the mormyrid electrosensory lobe and postsynaptic control over presynaptic spike occurrence and amplitude through an electrical synapse. J. Neurophysiol. *97*, 2191–2203.

Neuron, Volume *83*

Supplemental Information

Network Structure within the Cerebellar Input Layer Enables Lossless Sparse Encoding

Guy Billings, Eugenio Piasini, Andrea Lőrincz, Zoltan Nusser, and R. Angus Silver

1 Supplemental data

Figure S1, related to Figure 1

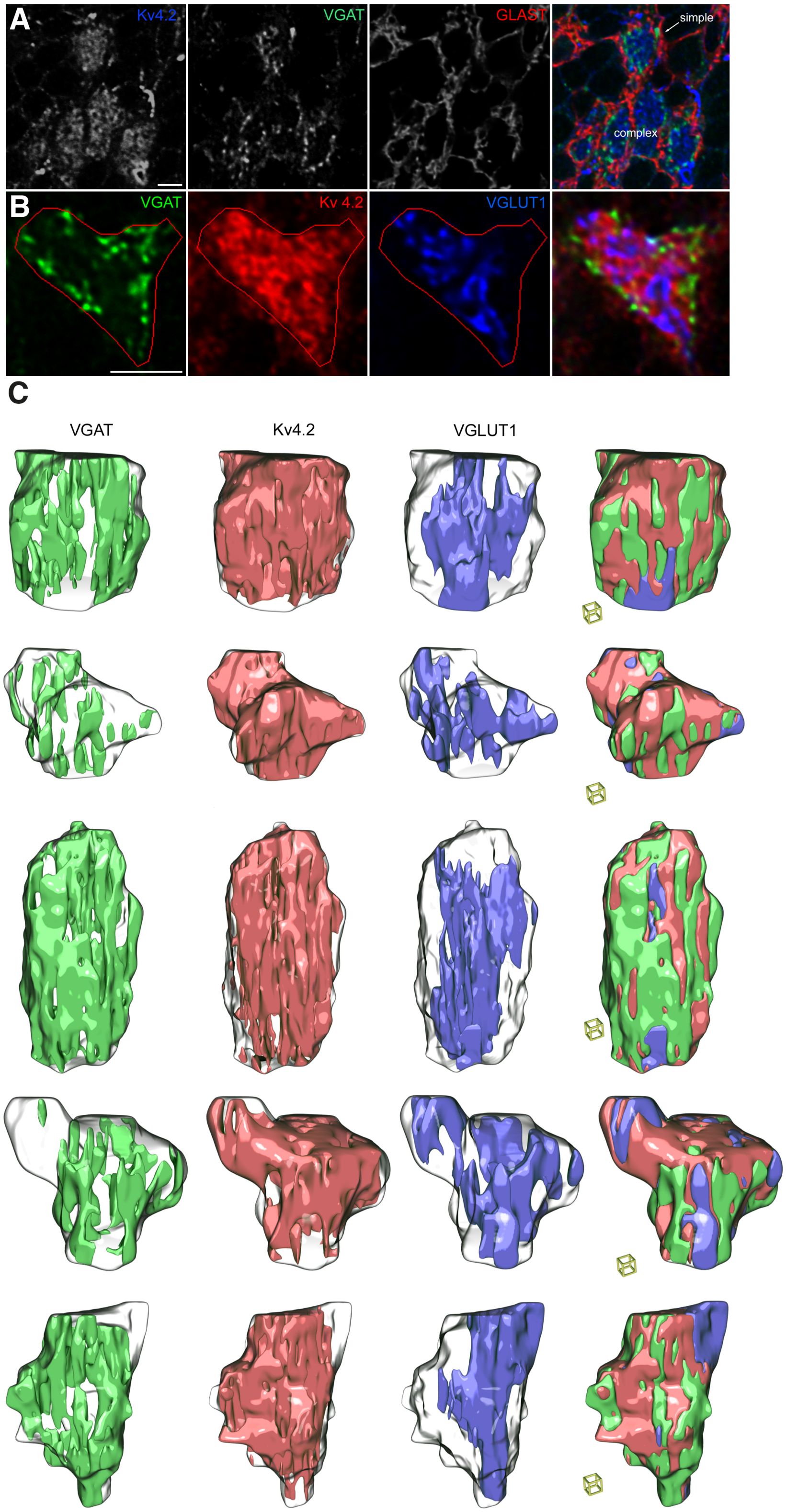

Figure S1, related to Figure 1: Molecular identification and reconstruction of cerebellar glomeruli. A: Immunolabelling for Kv4.2, VGAT and GLAST reveals a complex structure assembled from several simple glomeruli. B: A single confocal section of a simple glomerulus. Within the glomerulus a single VGLUT1 immunopositive mossy fiber synaptic rosette (blue) is present in the central position and it is surrounded by Kv4.2 subunit-immunolabeled granule cell dendrites (red) and by VGAT immunoreactive Golgi cell axon terminals (green). Scale bars: 5 µm. C: Five representative glomeruli reconstructed from confocal image stacks. Inside the glomerulus VGAT immunoreactive Golgi cell axon terminals (green) and Kv4.2 subunit-immunolabeled granule cell dendrites (red) enclose a single VGLUT1 immunopositive mossy fiber synaptic rosette (blue). Each edge of the cube: 1 μm.

Figure S2, related to Figure 2

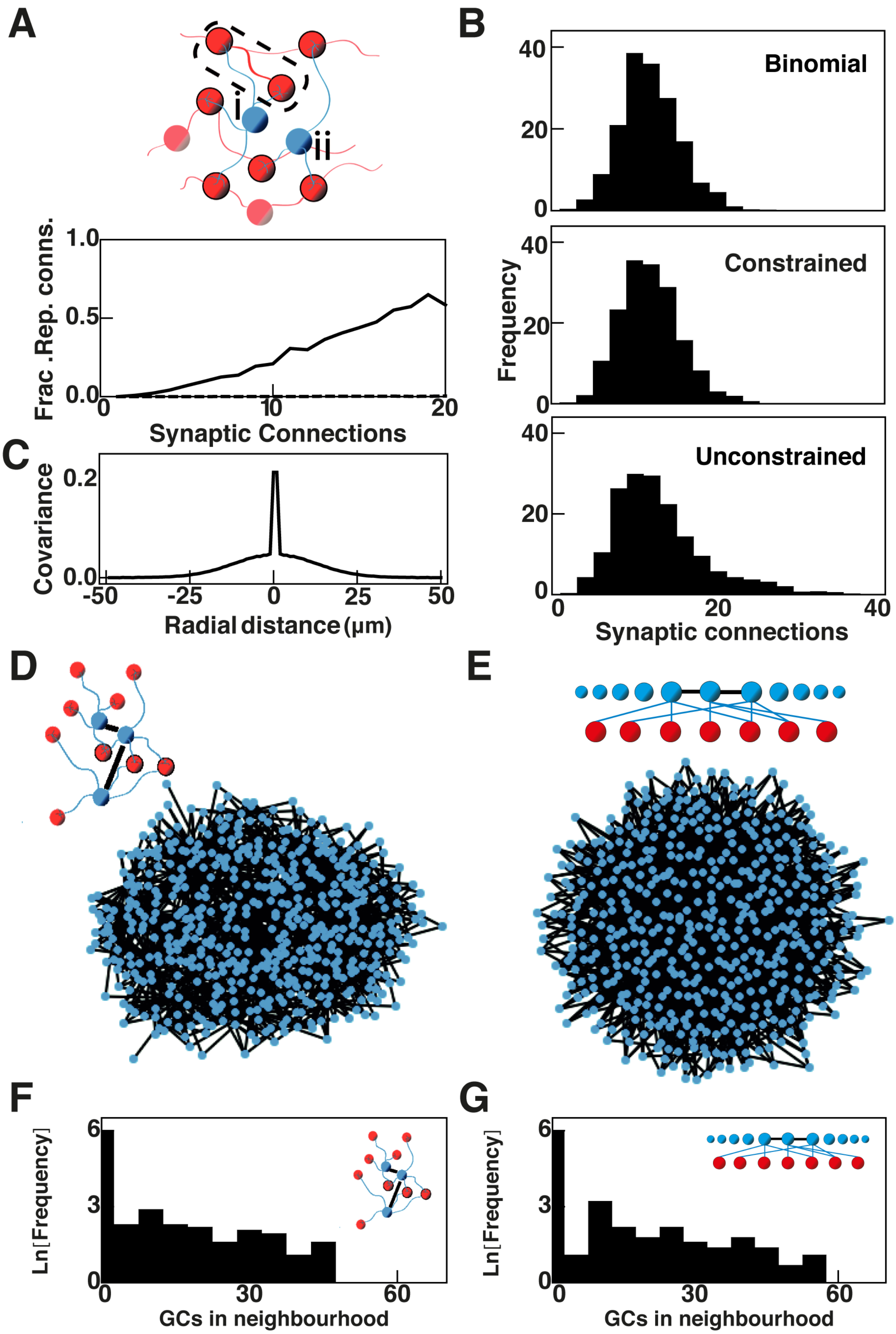

Figure S2, related to Figure 2: Connectivity constraints in the anatomical model produce a binomial distribution of the number of synaptic connections per mossy fiber rosette and biologically plausible dendrite lengths. A: Top: schematic diagram illustrating how multiple synaptic rosettes (glomeruli; red balls) are associated with each mossy fibers (MF; red lines) and distributed in 3D space. These rosettes/glomeruli may in principle be on the same fiber (eg. enclosed with dashed box) or on independent fibers. GCs (blue balls) connect to a subset of these (highlighted balls) within range of their dendrites (blue lines, known to be $\sim 15\mu m$ long on average). For each GC, there are several ways in which connectivity can give rise to non-uniform numbers of connections with distinct MFs including the case where two dendrites contact a single MF synaptic rosette. Here we illustrate i) A GC (blue) with $d = 4$ synaptic inputs, each made onto a different dendrite, but connects to a single MF via two separate synaptic rosettes and ii) Given some connectivity constraint (e.g. dendrite length limit) GCs can fail to connect all dendrites to d glomeruli. Bottom: Fraction of the GCs that do not have d independent inputs as a function of the number of synaptic connections per GC in a model with random connectivity (solid black line) and the same fraction in the model having connectivity constraints (see Supplemental Experimental Procedures) so as to avoid these effects (dashed black line) B: Binomial distribution with a mean of 12, which matches the expected number of synaptic connections per MF in the anatomical model (top), distribution of number of synaptic connections per MF synaptic rosette in anatomical model with constrained random connections (middle), distribution of number of synaptic connections per MF in anatomical model with unconstrained random connections (bottom). C: Mean covariance of GC activity versus distance from the center of the sphere in a *binary local GCL model* (where GCs were represented by binary linear threshold units, and connectivity was anatomically constrained) with 4 MF connections per GC. D: The neighborhood graph, formed by taking GCs as nodes (blue circles) and adding edges (black lines) between those GCs when the GCs share any MF inputs (red circles). The neighborhood graph for the anatomically constrained local GCL network is shown below. E: As D but for the uniform binary

model. More uniform distribution of nodes in this graph indicates a more even sharing of inputs in the uniform binary model. F: The distribution of the natural log of the number of neighborhood sizes indicates the number of other GCs that each GC directly shares inputs with. G: As F but for the bipartite graph. In the uniform binary model, GCs tend to have a larger local neighborhood.

Figure S3, related to Figure 2

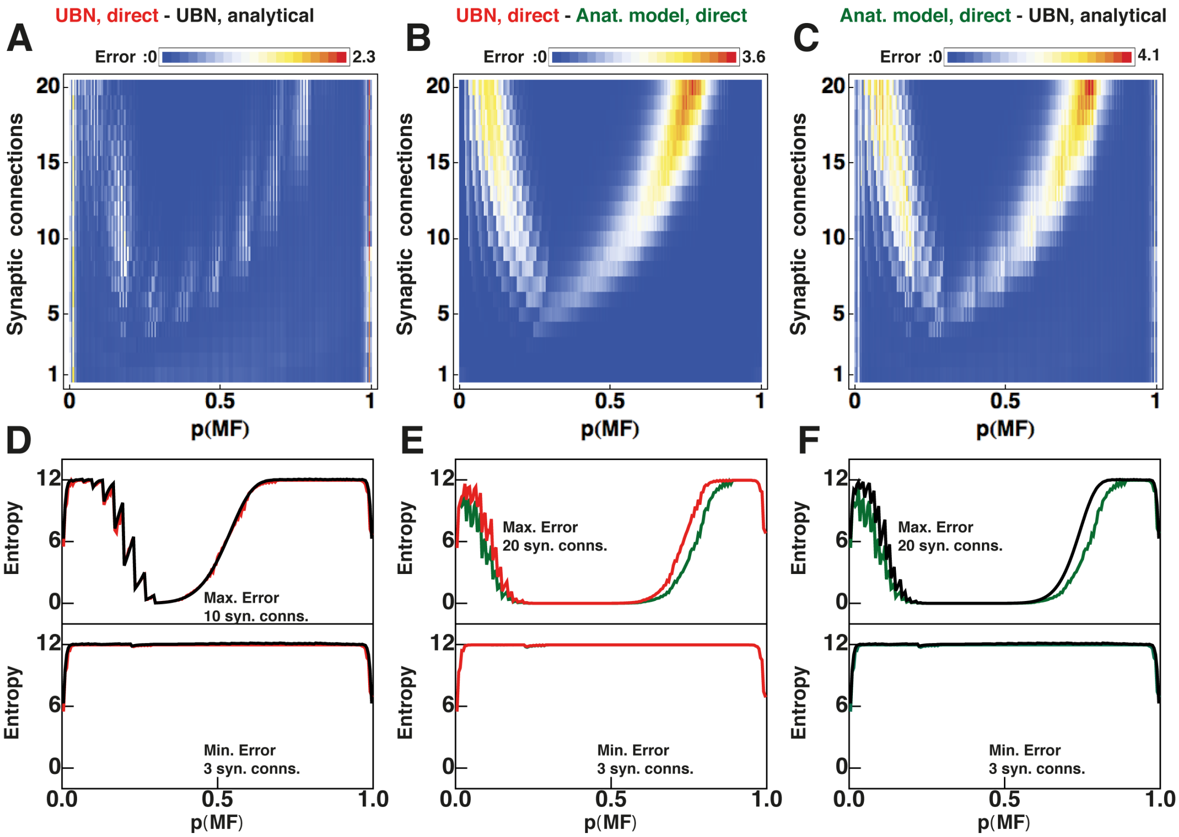

Figure S3, related to Figure 2: Comparison of analytical method for estimating granule cell entropy to direct numerical evaluation. A: Absolute difference ($\langle \Delta H \rangle$, labeled 'Error') of the direct and analytical estimates for the granule cell (GC) entropy encoded from 4000 events in the uniform binary network (UBN) model. Plot shows difference as a function of the number of synaptic connections per GC and the mossy fiber (MF) activity level $p(MF)$. B: As A but for the discrepancy in the direct estimate for the 4000 event entropy between the UBN and the anatomical local GCL model. C: Discrepancy between the direct estimate for the anatomical model and the analytical calculation for the UBN. Note different color scales. D: Top: Entropy encoded after 4000 events for case with 10 synaptic connections, which has the maximum error in A. Black line is entropy determined by our analytical method, red line is data for explicitly simulated UBN. Bottom: Comparison for lowest error case when the network has 3 GC dendrites. E: As D but for comparison of the direct estimate in the UBN (red) and the anatomical model (green); maximum and minimum error in B occurring for 20 GC dendrites

(top) and (respectively) 3 GC dendrites (bottom). F: As E but for comparison of the direct estimate in the anatomical local GCL model (green) and the analytical calculation for the UBN (black) These results show that the errors in our approach are modest with the largest error arising from the simplification of the anatomical local GCL model to a bipartite graph. Data shown is for networks with network activity dependent GC threshold (NADT) = 3.

Figure S4, related to Figure 7

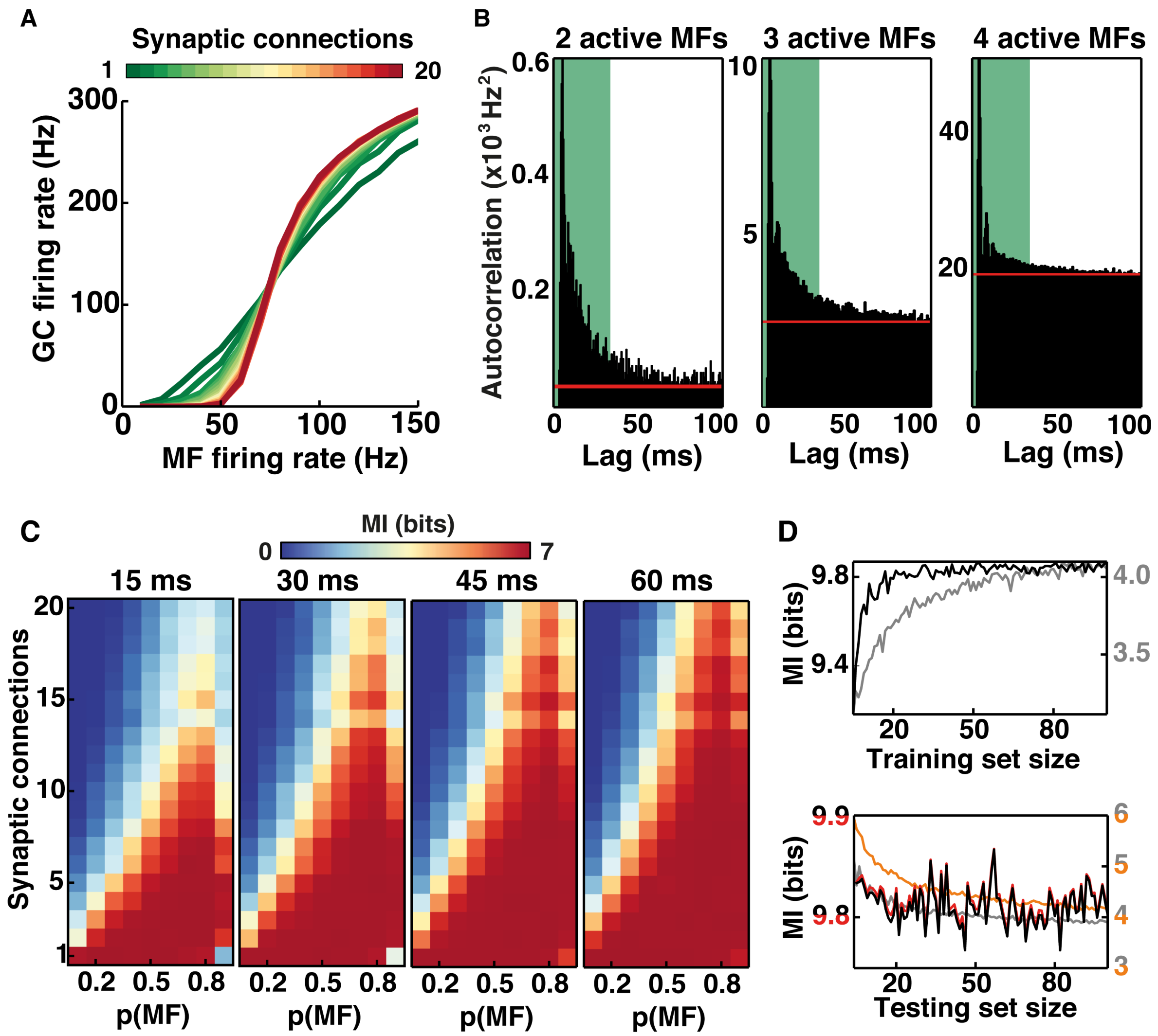

Figure S4, related to Figure 7: Effect of changing the number of synaptic connections on the granule cell input-output relationship, time window of synaptic integration in granule cells and the dependence of encoding on the integration window of the decoder. A: rate-coded input-output curve for the spiking granule cell (GC) model with a variable number of mossy fiber (MF) synaptic inputs per GC. B: autocorrelation of the spiking GC model output (with 4 synaptic inputs) for 2, 3 or 4 active inputs (the case of 1 active input is not shown as the resulting firing rate is very low). Red line: constant value of the autocorrelation of a Poisson point process with the same mean intensity. Green area: 30ms time window. C: Mutual information (MI) for 128 patterns, estimated for different lengths of the time window over which network activity is recorded.

D: Top: MI as a function of the number of repetitions per pattern used to train the decoder, for 1024 MF patterns, 4 synaptic connections and $p(MF)$=0.1 (gray) or $p(MF)$=0.5 (black). Note the different scales. The network performs poorly for $p(MF)$=0.1, transmitting only a fraction of the information present in the input, so this is a particularly stringent test for the performance of the decoder. Bottom: MI as a function of the number of repetitions per pattern used to estimate it, showing the magnitude of undersampling bias and the effect of our chosen bias correction technique. Grey and orange: 4 synaptic connections, $p(MF)$=0.1, quadratic extrapolation and no undersampling bias correction, respectively. Black and red: 4 synaptic connections, $p(MF)$=0.5, quadratic extrapolation and no undersampling bias correction, respectively.

Figure S5, related to Figure 8

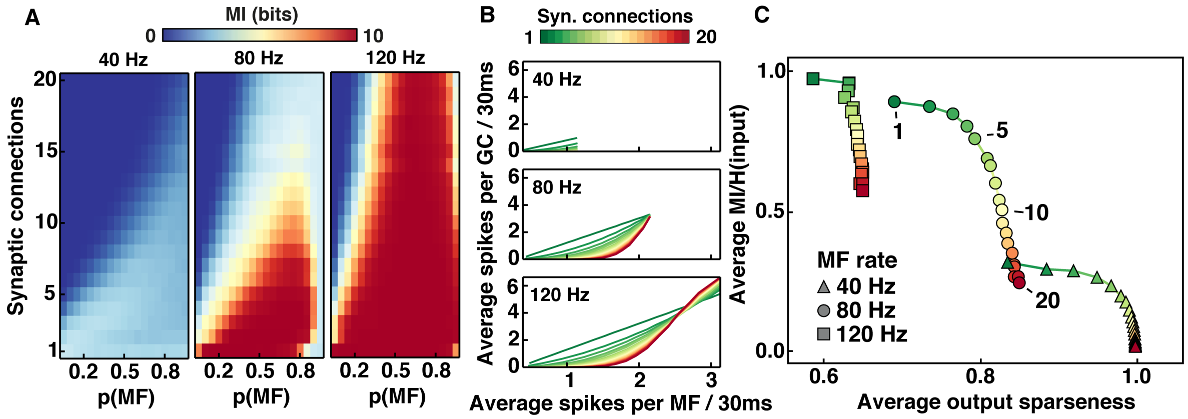

Figure S5, related to Figure 8: Effect of different mossy fiber input rates on spiking granule cell layer network performance. A: Mutual information (MI) for 1024 input patterns for active mossy fibers (MFs) firing at different rates, with tonic inhibition kept fixed at the average experimental value. B: average spikes per granule cell (GC) versus average spikes per MF within a 30 ms window, across all patterns and all values of the probability of MFs being active ($p(MF)$). Different color lines show relationships for networks with different numbers of synaptic connections. C: Average MI, normalized by the input entropy, versus average output sparseness (with averages taken across all values of $p(MF)$) for different active MF rates, parametrized by the number of synaptic connections. Note how, as shown in at the bottom of panel B, there exists a threshold level of MF activity above which the relationship between number of synaptic connections and GC activity is reversed, and the most active networks are those with more connections. This is reflected in panel C, as the networks with many synaptic connections lose the sparsification advantage they have when the inputs are encoded at lower rates, leaving those with fewer connections as an optimal choice for lossless sparse encoding, even though the maximum sparsification attainable is lower.

Figure S6, related to Figure 8

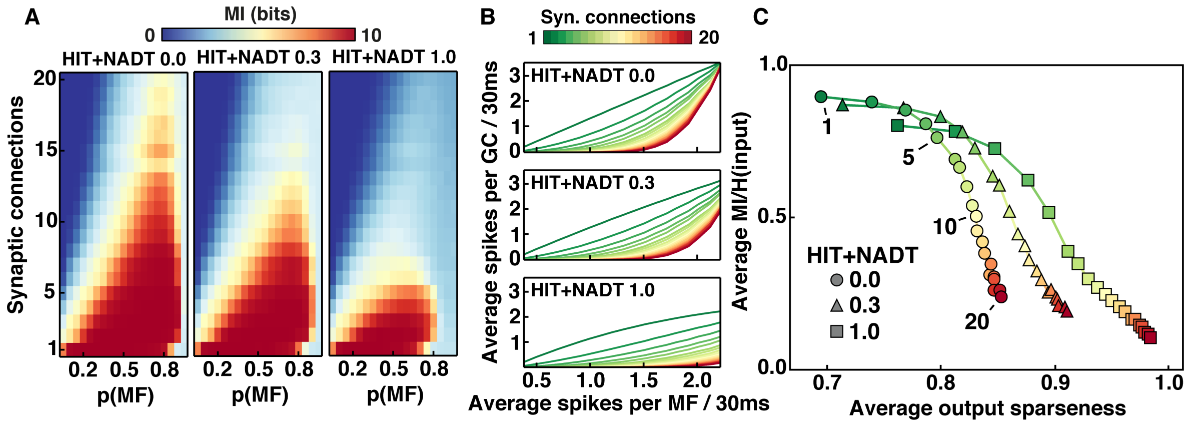

Figure S6, related to Figure 8: Effect of network activity-dependent scaling of tonic inhibition. A: Mutual information (MI) for 1024 patterns of different active mossy fibers (MFs) with different levels of scaling of tonic inhibition in granule cells (GCs) with network activity (analogous to NADT for the uniform binary model) added on top of the physiological baseline (indicated as HIT for consistency with Figure 6 in the main text); heatmap on the left (NADT=0) is included for comparison and represents the baseline case. In these simulations an extra amount of GABA_AR-mediated conductance, proportional to the fraction of active MFs ($p(MF)$), was introduced. The NADT parameter is a scaling coefficient for this proportionality relation. For example, for NADT=1 and $p(MF)$=0.6 the GABA_AR-mediated conductance will be increased by 60%, whereas for the same $p(MF)$, but NADT=0.3 the increase will be of 20%. B: average spikes per GC versus average spikes per MF across all patterns and all values of the probability of MFs being active ($p(MF)$), parametrized by the number of synaptic connections for the different levels of NADT. C: Average MI, normalized by input entropy, versus average output sparseness (with averages taken across all values of $p(MF)$) for different values of NADT parametrized by the number of synaptic connections. The value of NADT=0.3, which is our rough estimate of how the time-averaged GABA_AR conductance contributed by Golgi cells varies with network activity (Ward, Piasini and Silver, unpublished observations), increased the performance for networks with few synaptic connections.

Table S1, related to Figure 1: Size of the cerebellar glomeruli and their components

	GC dendrites (Kv4.2)		Golgi axons (VGAT)		Mossy fiber synaptic rosette (VGLUT1)		Glomerulus		
	Mean ± SD	Range	Mean ± SD	Range	Mean ± SD	Range	Mean ± SD	Range	
Rats	(μm^3)	(μm^3)	(μm^3)	(μm^3)	(μm^3)	(μm^3)	(μm^3)	(μm^3)	N
#5	184.5 ± 37.9	$108.7 - 231.8$	122.2 ± 42.7	$61.1 - 190.3$	96.2 ± 33.7	$45.6 - 144.3$	402.8 ± 106.8	$215.3 - 549.4$	10
#6	250.3 ± 144.8	$123.8 - 496.6$	148.6 ± 68.1	$44.4 - 228.4$	148.5 ± 58.8	$67.6 - 214.4$	547.4 ± 256.8	$235.8 - 939.3$	5
#1	152.6 ± 50.2	$92.9 - 228.2$	112.2 ± 44.4	$36.5 - 181.5$	88.1 ± 20.9	$46.0 - 109.6$	352.9 ± 92.3	$200.7 - 480.2$	9
Mean	195.8 ± 49.8		127.7 ± 18.8		110.9 ± 32.8		434.4 ± 100.9		

Table S2, related to Figure 1: Density of mossy fiber glomeruli

	Rats			
	#5	#6	#1	Mean
GCL occupied by glomeruli (%)	30.6 ± 5.5	26.3 ± 4.9	29.5 ± 4.8	28.8 ± 2.3
Density of glomeruli ($1/mm^3$)	7.5×10^5	4.9×10^5	8.5×10^5	$6.6 \pm 1.5 \times 10^5$

2 Supplemental experimental procedures

2.1 Measurement of granule cell and glomerular density

2.1.1 Tissue preparation and fluorescence immunocytochemistry

Four thirty-day old Sprague-Dawley rats were deeply anesthetized with Halothane and 7% Chloral hydrate (0.4ml), then perfused through the aorta first with saline then with 4% paraformaldehyde in 0.1M phosphate buffer (PB, pH = 7.3) for 12 min. The brains were immediately removed and blocks were cut out from the cerebellum. 40µm thick sagittal sections were cut with a Vibratome (VT1000S, Leica Microsystems, Vienna, Austria) and washed overnight in 0.1M PB. Normal goat serum (NGS, 10%) in Tris-buffered saline (TBS, pH =7.4) was used for blocking, followed by incubations in primary antibodies. Sections for labeling cerebellar glomeruli were incubated in the mixture of guinea pig anti-VGAT (1:500; Calbiochem, Darmstadt, Germany), mouse anti-Kv4.2 (1:1000; NeuroMab, UC Davis, CA), and rabbit anti-VGLUT1 (1:500; Synaptic Systems, Goettingen, Germany) or rabbit anti-GLAST (1:500; gift from Prof. Shigemoto) antibodies diluted in TBS containing 2% NGS and 0.05% Triton X-100. Following several washes, sections were further incubated in the mixture of Alexa 488 goat anti-guinea pig (1:500; Molecular Probes, Leiden, The Netherlands), Cy5 conjugated goat anti-rabbit IgG (1:500, Jackson ImmunoResearch, West Grove, PA) and Cy3 conjugated goat anti-mouse IgG (1:500, Jackson ImmunoResearch) for 2 hours. Sections were mounted on slides in Vectashield (Vector Laboratories, Burlingame, CA).

2.1.2 Image acquisition

Images were taken from parasagittal vermis sections, from the proximal part of lobule VI (lobule VIa). Immunofluorescence labeling was visualized by a confocal scanning microscope (FV1000, Olympus Europe, Hamburg, Germany). Auto-

mated sequential acquisition of multiple channels was used for multi-color images to avoid spectral crosstalk between channels. Series of 25-37 confocal images (800 X 800 voxels) spaced at 0.5, 1 or 3 μm apart were acquired using either a 40X (NA=0.85) or a 60X (NA=1.3) objective lens.

2.1.3 Calculation of the density of granule cells

Granule cells (GCs) were identified by the Kv4.2 immunolabeling, which outlines the somato-dendritic plasma membranes of GCs. The two-way dissector method (Gundersen et al., 1988) was used to determine GC density within GC layer (GCL). The distance between the sections was chosen to be about 30% of the average height of the objects to be counted. The mean diameter of GCs was found to be 6.7 μm, resulting in a real dissector distance of 2 μm. A weak fixation was used to achieve maximal penetration of the antibodies into the tissue. However, such a mild fixation resulted in considerable tissue shrinkage and thickness of the section when mounted on a glass slide and covered with a coverslip. Accordingly, the axial diameter of the GCs was reduced, thus the dissector distance had to be corrected for shrinkage. To evaluate the level of tissue shrinkage we calculated a shrinkage factor (0.57 ± 0.09, n= 3 rats, 3 slices per rat) for individual slices by dividing the thickness of the slice after processing with the slice thickness before processing (40 μm). Indeed, the height of GCs collapsed to 57-59% along the Z-axis. We did not correct for XY direction, as the tissue XY shrinkage was negligible (< 2%).

Pairs of reference and look-up optical sections were randomly selected from the recorded image stacks. Areas for counting were sampled by counting frames (300x300px, 3546.2μm^2) positioned on the images (Figure 1B1 & B2). Cells cut by the acceptance line (green), but not by the forbidden line (red) were included in counting. A cell was counted if the transect from the cell was seen on the reference image but not on the corresponding look-up section. Two counting frames per section (in two-way) and 3 slices per animal were used for calculating

the numerical density of GCs (ρ_{GC}),

$$\rho_{GC} = \frac{\text{total cells counted}}{\text{total area of frames} \times \text{real dissector distance}}.$$

2.1.4 Measurement of the density of cerebellar glomeruli

Mossy fiber (MF) synaptic rosettes, GC dendrites and Golgi cell axon terminals forming a cerebellar glomerulus were identified by, respectively, VGLUT1, Kv4.2 and VGAT immunolabeling (Figure 1C-G; Figure S1). A MF synaptic rosette, which is typically en passant, forms the core of each simple glomerulus. This is surrounded by GC dendrites and by Golgi cell axon terminals. The whole glomerulus is ensheathed by glial processes immunopositive for the glutamate transporter GLAST, Figure S1. Some glomeruli were clumped together forming complex glomeruli. Visualization of the different components showed that they were formed from 2-5 simple glomeruli, Figure S1. Confocal image stacks of simple and in some cases complex glomeruli were acquired at 0.2-0.4 μm axial resolution and were de-convolved by the maximal likelihood estimation algorithm implemented in the Cell P software (Olympus Soft Imaging System, Munster, Germany). Color channels representing VGLUT1 (blue), Kv4.2 (red) and VGAT (green) immunolabeling were binarised and volume calculations were performed in Matlab (MathWorks Inc., Natick, USA-MA). Within the glomerulus the volume of each voxel was weighted by the intensity of corresponding RGB values and volume fractions for the three profiles were calculated, Figure S1. Density of glomeruli was calculated from the mean volume of glomeruli and the mean volume of GCL occupied by glomeruli.

2.2 Anatomically constrained local granule cell layer model

Given the measured ratio of glomeruli to GCs and the number of MF synaptic connections per GC, then the mean number of synaptic connections per MF rosette is fixed. However, in practice the MF rosette degree distribution (i.e. the distribution of the number of synaptic connections per rosette) depends upon the shape of the 3D volume used to construct the model. This occurs because GC soma within the defined volume of the model can connect to glomeruli located outside by virtue of the finite length of GC dendrites. This changes the effective glomerular to GC ratio and also causes the glomeruli outside the GC cell body volume to be sampled less frequently than those within it, affecting the connectivity statistics of the local network. We found that confining the whole network, including all dendrites, within a sphere minimized these effects (e.g. Figure S2). Another problem with constructing networks was that randomly connecting MF rosettes and GCs resulted in some GCs sampling the same rosette twice or the same MF more than once via differing glomeruli (Figure S2A, top). For a network having only 4 dendrites this effect was small (~ 4%) and consistent with experimental observations, but this problem grew as the number of synaptic connections per GC was increased, causing the number of independent MF inputs to depend upon the number of dendrites. We overcame these problems by imposing a constraint on the GC dendrite lengths which, while allowed in principle to assume any value, are as close as possible to $15\mu m$, and by explicitly forcing GCs to have all dendrites connecting to independent MFs (Figure S2A, bottom). This enabled us to generate a spherical network model with a MF rosette degree distribution that was binomial in shape, rather than one with a tail (Figure S2B), as well as a dendrite length distribution having mean $17\mu m$ and mode 15μm, Figure S2C. Indeed, most dendrite lengths were $< 20\mu m$ for 4 dendrites per GC (Figure S2B), as found experimentally (Eccles et al., 1967; Palkovits et al., 1972).

2.3 Uniform binary network model

The structure of the uniform binary network model is formally equivalent to a random bipartite graph consisting of two disjoint sets of nodes with inputs representing MF rosettes and outputs representing GCs, connected by randomly placed edges (synaptic connections), such that each edge joins an input node to an output node. No edges are permitted between two inputs or between two outputs. We applied the added constraint that each output node can only connect to a fixed number of edges, representing the number of synaptic connections per GC. In this manner we constructed a random uniform binary model with connectivity statistics as close as possible to the 3D anatomically constrained local granule cell network model. While the degree distribution of MF rosettes in the random bipartite graph matched the anatomically constrained model, the spatial structure of the connectivity statistics was lost. Because the GCs in the anatomically constrained local GCL model connect by favoring dendrites of around 15μm, they tend to share MFs with nearby GCs, introducing a spatial dependence that does not exist in the more abstract uniform binary network model. In the bipartite graph connections are not influenced by the dimensions of the network, since a particular GC can connect to any MF with equal probability. This is an important assumption in the method of calculating the entropy of the GCs in the bipartite graph (see Appendix below). This can be more clearly seen when we compare connectivity in the anatomically constrained model and bipartite graph by means of their respective neighborhood graphs (Figure S2D,E). The GCL neighborhood graph consists of GCs as nodes with edges between any GCs that mutually connect to at least one MF. Hence it depicts the tendency of GCs to share MFs. The neighborhood graphs were plotted with spring-electric embedding (using the integrated function 'GraphPlot' in Mathematica (Wolfram Research, Champaign, USA-IL), such that each node repels other nodes in a Coulombic manner while exerting an attractive force on its neighbors in the manner of Hooke's law. The graph plotted is the equilibrium configuration of this system.

In 'spring-electric embedding' representations of networks the edges of the graph

are more evenly distributed the more evenly separated the nodes of the graphs are, such that all nodes have an approximately isotropic attraction/repulsion to other nodes. The neighborhood graph for the anatomically constrained local granule cell network model exhibited a flattened spherical shape due to clustering of GCs, caused by the restricted dendrite lengths (Figure S2D). In contrast, the neighborhood graph of the bipartite network had a spherical shape due to the spatially uniform connectivity (Figure S2E). Moreover, the bipartite network contains more GCs that share inputs with large numbers of other GCs (Figure S2F &G). However, the differences between the anatomically constrained local GCL network and the uniform binary model are relatively small because the mean span of the dendrites is comparable to the radius of the network. This resulted in similar information transmission properties when we compared the properties of binary networks constructed with the anatomically constrained and the uniform binary model structures and calculated information with the direct method (see Figure S3). Moreover, the systematic error introduced by differences in network structure overestimates the encodable range of networks having many connections. Hence, correction of the error would serve to strengthen the finding that networks having many synaptic connections per GC encode a smaller range of MF activity (Figure S3F).

2.4 Local granule cell layer network size and calculation of granule cell covariance

We set the network radius to $40\mu m$, which corresponds to approximately twice the GC dendrite length since this is the largest distance over which two GCs would be likely to sample the same input. To test whether this was indeed the case we computed the GC covariance as a function of network radius for a binary variant of the anatomically constrained local GCL network model. The covariance matrix was determined for a network having 4 synaptic connections per binary GC, threshold of 2 and $p(MF) = 0.5$ for 4000 events. Values in the covariance

matrix were plotted as a function of the corresponding pairwise distance between GCs (Figure S2C). Covariance between cells was zero within a radius of $40\mu m$, confirming our prediction from anatomical considerations. While variation in the parameters used changed the magnitude of covariance, it did not alter its spatial profile.

2.5 Relative fixed threshold and network activity-dependent granule cell threshold function in uniform binary network models

We implemented a relative fixed threshold in the uniform binary network, which was analogous to keeping a fixed tonic inhibition in real cells but scaling down the size of the excitatory synaptic conductances when increasing the number of MF synaptic connections per cell. In practice, this was implemented by setting the GC threshold to 75% of the inputs or the nearest integer value above this using ceiling[$0.75 \cdot d$]. Furthermore, to explore how information processing in uniform binary networks was affected by a network activity-dependent granule cell threshold function as is likely to be mediated by the feedforward inhibitory Golgi circuit (Kanichay and silver, 2008; Sawtell, 2010), we introduced a dependence between the threshold of the GCs and the probability of MF activation ($p(MF)$). To do this we set the GC threshold to progressively higher levels with $p(MF)$ using a piecewise constant function. The 'steepness' of this relationship is controlled by a parameter setting the network activity-dependent threshold, denoted 'NADT'. The NADT was defined as $1/(d \times \text{interval length})$ where the interval length is the width of the constant $p(MF)$ regions of the threshold function and d is the number of synaptic connections per GC. Higher NADT implied a smaller interval length and a correspondingly steeper change of the GC threshold with MF activation, thus mimicking greater inhibition.

2.6 Calculation of Shannon information in uniform binary networks

For binary networks we determined the Shannon information of the GC output and compared it to the raw event information encoded in the MFs inputs to assess the information transmission efficiency through the network. Since signal transmission in these networks is noise free, the Shannon information is equal to the raw output entropy. We defined the encodable information as that arising from the set of sensory-motor states that the animal could encounter during its lifetime. To do this we assumed N events occur in the lifetime of the animal, with one unique event occurring in each small time interval, which was taken as $30ms$, implying a maximum of 1 billion events in the 1 year lifespan of a small rodent in the wild. The binary MF code representing these events was defined as the randomly realized state (0 or 1) of the MF inputs to the network. Unfortunately the direct method for calculating information (Eq.(1)) is excessively computationally demanding when considering such a large number of distinct MF input patterns and the need to average across many instances of an individual network, given the random nature of their synaptic connectivity. Moreover, this was compounded by the fact that we wanted to systematically investigate networks with different numbers of synaptic connections and different MF activation probabilities. We therefore developed an alternative, analytical method for rapidly calculating information in uniform binary networks. The maximum information carried by the GC output is equal to the entropy of the MFs (H_{MF}), but the entropy realized by the GCs H_{GC} depends on the number of synaptic connections per GC (d), their thresholding ϕ and their numerosity γ. Our approach utilizes Eq. 29, given with its derivation in the Appendix (below), to calculate H_{GC}.

2.6.1 Identification of the sources of error in estimating entropy in uniform binary network models

Since information transmission can be computed by explicit enumeration of input and output codes for a modest number of patterns, we used this approach

to test the validity of our analytical approach for calculating H_{GC} and for examining the effect of reducing the anatomically constrained local network structure to a uniform bipartite network. Connections in the anatomically constrained local network were described by a connection matrix **A**, with elements $a_{ij} = 1$ if MF i connects to GC j and $a_{ij} = 0$ otherwise. In the anatomically constrained local network this matrix was determined by the 3D spatial pattern of connectivity between GCs and glomeruli which was generated as described above, where as in the case of the uniform binary model this was simply a random matrix with the only constraints that GCs could connect each MF only once and each GC connected to exactly d MFs. To simulate network activity, a random binary vector **x** representing an MF activation pattern was multiplied by the connection matrix. Each element of the resulting vector was then thresholded by the GC threshold level to yield the binary GC outputs $\mathbf{y} = \Phi(\mathbf{Ax})$. The mapping from MF patterns to GC patterns ($\mathbf{x} \rightarrow \mathbf{y}$) was therefore random, by virtue of the random network connections, but note that in the event of a repeat input **x** the identical GC pattern **y** would reoccur; this was justified by the assumption that the noise arising from synaptic transmission and firing was negligible over each 30 ms integration window.

Generation of output patterns from input patterns in this manner enables the entropy of the output patterns to be determined. For N events, M unique input patterns may be produced ($M = N$ only if each MF realization is unique for every event), which then map to G unique GC patterns (in general, $G \leq M$ since some of the M MF patterns could map to duplicate GC patterns). The mutual information between these G GC vectors and the N events for a particular network can be characterized by the empirical entropy (Cover & Thomas, 1991; Shannon, 1948) of the GC patterns since our network is noise free,

$$H_{GC} = -\sum_{k=1}^{G} p(\mathbf{y}_k) \log_2[p(\mathbf{y}_k)] \quad (1)$$

where $\mathbf{y}_k$ is a binary GC state vector and $p(\mathbf{y}_k)$ is the empirical probability of that GC state given a particular realization of the random network connections; $p(\mathbf{y}_k) =$

Q_k/N where Q_k is the number of times that GC output vector k occurs over the N events. Note that no undersampling bias correction (Treves and Panzeri, 1995) is required, since given a single network and a single set of MF patterns, the mapping from MFs $\rightarrow$ GCs is deterministic.

The empirical entropy of the MFs (H_{MF}) is determined by the probability of activation of an individual input $p(MF)$, the number of inputs μ and the number of events. The information rate of MF pattern generation is the standard quantity

$$-\mu[p(MF)\log_2(p(MF)) + (1-p(MF))\log_2(1-p(MF))] \quad (2)$$

and is the value that the empirical entropy converges to for an infinite number of events. The maximum information rate of MF pattern generation (occurring when $p(MF) = 0.5$) is the input capacity of the network $C = \mu$ bits and is the upper limit of the event information that *could* be represented by the binary MFs.

To examine error in the estimation of GC entropy using our analytical approach (Eq. (29); Appendix) we compared the GC entropy in binary networks with a directly simulated uniform binary network for a smaller number of MF input patterns, using the direct method. For 4000 MF patterns we are able to estimate GC entropy over the full range of parameter space (Figure S3). The discrepancy between the analytical calculation method and the direct calculation method never exceeded a fraction of a bit for the region of parameter space we explored (Figure S3A,D). However a larger discrepancy was observed between the uniform bipartite graph networks and the anatomically constrained network model, both calculated with the direct method (Figure S3B,E) or with the analytical and direct method, respectively ((Figure S3C,F). Thus the error arising from the the differences in connectivity between the anatomically constrained network model and the bipartite graph is larger than that introduced by our rapid analytical method for calculating entropy in bipartite networks. The maximum error introduced by simplifying network structure is less than 1 bit for networks up to around 10 connections but is more pronounced for networks having many connections (Figure S3B,E).

2.7 Biologically detailed spiking network model

2.7.1 Mossy fiber spike train generation

The anatomically constrained local GCL network structure was used to construct the biologically detailed spiking network models. For each simulation configuration, a variable fraction $p(MF)$ of the 176 total MF synaptic inputs were randomly selected and designated as active. When simulating spatially correlated inputs, MF terminals were activated in groups of 5, starting by selecting one terminal at random and activating it together with its 4 closest neighbors. For each repetition of the simulation configuration, active MF input spike trains were then generated as instances of the Poisson stochastic process with a mean rate of 80Hz, except for simulations in Figure S5 where other rates were explored. For the remaining inactive MFs, Poisson spike trains of 10 Hz rate were generated. The NEURON code generated by neuroConstruct used NEURON's built in NetStim and mcell_ran4 for pseudorandom event generation.

2.7.2 Synaptic conductance models

The active and inactive MF spike trains were then used to build trains of synaptic conductances for each MF input (i.e.rosette). Each individual excitatory synaptic conductance occurred at the time of a MF spike and consisted of both AMPAR- and NMDAR-mediated conductance components (G_{AMPA} and G_{NMDA}, respectively). These synaptic conductances exhibited distinct waveforms and voltage and time dependencies, as well as short term plasticity as for real MF-GC inputs. Values for all the parameters of the synaptic models, together with their provenance (or the source of the relevant experimental data) are reported in Table S3.

AMPAR-mediated synaptic waveform. The total AMPAR conductance component (i.e from direct quantal release and glutamate spillover) arising from a

single MF spike is given by

$$G_{AMPA} = p_{AMPA,d} \cdot g_{AMPA,d} + p_{AMPA,s} \cdot g_{AMPA,s}$$

where

$$g_{AMPA,d} = \sum_{i=1}^{2} a_i^{(AMPA,d)} (e^{-t/\rho^{(AMPA,d)}} - e^{-t/\delta_i^{(AMPA,d)}})$$
$$g_{AMPA,s} = \sum_{i=1}^{3} a_i^{(AMPA,s)} (e^{-t/\rho^{(AMPA,s)}} - e^{-t/\delta_i^{(AMPA,s)}})$$

and $a_i^{(AMPA,d)}$, $\rho^{(AMPAd)}$, $\delta_i^{(AMPA,d)}$ and $a_i^{(AMPA,s)}$,$\rho^{(AMPA,s)}$,$\delta_i^{(AMPA,s)}$ indicate component amplitudes, rise times and decay times for the direct and glutamate spillover-only components of the AMPAR synaptic waveform, respectively. $p_{AMPA,d}$ and $p_{AMPA,s}$ are the independent short-term depression scaling factors for the direct and spillover components, respectively, and are both defined as per the standard NeuroML2 *TsodyksMarkramDepMechanism* component type, as described below.

NMDAR-mediated synaptic waveform. The total unblocked NMDAR conductance component arising from a single MF spike is given by

$$G_{NMDA} = p_{NMDA} \cdot \sum_{i=1}^{2} a_i^{(NMDA)} (e^{-t/\rho^{(NMDA)}} - e^{-t/\delta_i^{(NMDA)}})$$

where $a_i^{(NMDA)}$, $\rho^{(NMDA)}$ and $\delta_i^{(NMDA)}$ indicate conductance amplitudes, rise time and decay times for the two components of the waveform. The p_{NMDA} scaling factor implements short term depression *and* facilitation of the NMDAR component and is defined as per the standard NeuroML2 *TsodyksMarkramDepFacMechanism* component type, as described below.

Mg^{2+} block mechanism. A Mg^{2+} block mechanism for the NMDAR-mediated synaptic conductance component was modeled as a custom LEMS component type by defining an unblock function $b(V)$ with the Woodhull formalism (Rothman & Silver, 2014):

$$b(V) = \frac{C_1 e^{\delta_{bind}\theta V} + C_2 e^{-\delta_{perm}\theta V}}{C_1 e^{\delta_{bind}\theta V} + C_2 e^{-\delta_{perm}\theta V} + [Mg^{2+}]_{out} e^{-\delta_{bind}\theta V}}$$

where V is the postsynaptic membrane potential, and $\theta = zF/RT$, with z as the Mg^{2+} ionic charge, F the Faraday constant, R the ideal gas constant, and T the absolute temperature.

Synaptic plasticity mechanisms. The short term plasticity models in the simulations make use of NeuroML2's standard *TsodyksMarkramDepMechanism*[1] and *TsodyksMarkramDepFacMechanism*[2] component types. These, in turn, are defined as the models formulated in, respectively, Tsodyks & Markram (1997) and Tsodyks et al. (1998), simplified to ignore inactivation. For a synaptic conductance g, a plasticity mechanism defines a factor p which multiplies the contribution of a single synaptic event before it gets added to g.

As defined in the NeuroML2 documentation, *TsodyksMarkramDepFacMechanism* is parametrized by an *initial release probability* r, a *depression recovery time* Δ and a *potentiation recovery time* Π. p is defined as

$$p(t) = U(t) \cdot R(t)$$

where $U(t)$ and $R(t)$ are the internal dynamical variables of the model, which are initialized as

[1] *http://www.neuroml.org/NeuroML2CoreTypes/Synapses.html#tsodyksMarkramDepMechanism*
[2] *http://www.neuroml.org/NeuroML2CoreTypes/Synapses.html#tsodyksMarkramDepFacMechanism*

$$U(0) = r$$

$$R(0) = 1$$

Following a synaptic event, U and R get updated according to

$$U \rightarrow U + r \cdot (1 - U)$$

$$R \rightarrow R \cdot (1 - U)$$

while, in absence of synaptic events, they decay exponentially to their initial values:

$$\frac{dU}{dt} = -\frac{U - r}{\Pi}$$
$$\frac{dR}{dt} = -\frac{R - 1}{\Delta}$$

Finally, *TsodyksMarkramDepMechanism* is a simpler version of *TsodyksMarkramDepFacMechanism* where U is fixed to its initial value.

Derivation of synaptic model parameters from experimental data. Estimates for the parameters for the synaptic waveform shape and plasticity mechanisms were recomputed from the experimental data published in Rothman et al. (2009) with a particle swarm optimization algorithm (Deb & Padhye, 2010) implemented using the inspyred framework (Garrett, 2014). Parameters for the Mg^{2+} block mechanism were set to the values published in Schwartz et al. (2012).

2.7.3 Granule cell model

Granule cells were modeled as standard refractory conductance-based integrate-and-fire neurons as per the *IaFRefCell* component type definition[3] in NeuroML2. For these model GCs the membrane voltage evolves according to

$$-C_m\frac{dV}{dt} = G_m\cdot(V-E_m) + G_{GABAR}\cdot(V-E_{GABAR}) + \\ + \sum_{i=1}^{d} G_{AMPAR}(i;t)\cdot(V-E_{AMPAR}) + b(V)\cdot\sum_{i=1}^{d} G_{NMDAR}(i;t)\cdot(V-E_{NMDAR}) \quad (3)$$

where C_m is the membrane conductance, E_m the reversal potential of the membrane leak conductance, G_{GABAR} and E_{GABAR} the tonic GABAR-mediated conductance and reversal potential, $G_{AMPAR}(i;t)$ and $G_{NMDAR}(i;t)$ the AMPAR and NMDAR-mediated conductance trains computed from the MF input spike trains, E_{AMPAR} and E_{NMDAR} the AMPAR and NMDAR reversal potentials, and $b(V)$ the NMDAR Mg^{2+}unblock function defined above. Upon V reaching the threshold value V_t a spike is emitted, and V is then clamped to the reset potential V_r for a refractory interval τ_r. Values and experimental provenance for all parameters in the model are reported in Table S3.

2.7.4 Simulation management

Single cell and synaptic models were serialized as LEMS/NeuroML2 files. Instantiations of the anatomically detailed network model were generated with Mathematica (Wolfram Research, Champaign, USA-IL) and exported in the GraphML format (Brandes et al., 2002). These were loaded, respectively, through neuroConstruct's Jython scripting interface (Gleeson et al., 2007) and networkX (Hagberg et al., 2008) into custom Python software that generated NEURON simulations (Carnevale and Hines, 2006) through neuroConstruct, distributing the computational load from code generation and simulation across two HPC platforms (the SilverLab's own cluster and UCL's Legion cluster) using the Sun Grid

[3] http://www.neuroml.org/NeuroML2CoreTypes/Cells.html#iafRefCell

Engine job queuing system. Spike time data was stored in compressed hdf5 archives using h5py (Collette, 2013).

2.8 Analysis of spike trains

Mutual information (MI) was calculated between the set of N MF input patterns and N output network activity classes obtained by performing a Voronoi (nearest-neighbor) tessellation of the output space. N was the smallest number of output classes that allowed full recovery of information, given that we assumed a uniform prior over the inputs. The N seed points for the tessellation were the centroids of the clusters obtained by running the k-means algorithm (Lloyd, 1982) as implemented in scikit-learn (Pedregosa et al., 2011) on a training dataset of 30 repetitions per pattern. To avoid being trapped in local minima, the algorithm was re-initialized 10 times using k_means++ (Arthur & Vassilvitskii, 2007), and the best clustering solution was used in the computation of the centroids. Training data was not re-used for the computation of MI. Information, which had an upper bound equal to the input entropy ($\log_2 1024 = 10$ bits for 1024 patterns), was calculated with the pyentropy package (Ince et al., 2009). Undersampling bias in the MI estimate (Panzeri et al., 2007) was accounted for with the *quadratic extrapolation* procedure (Strong et al., 1998) (Figure S4D, bottom). Other bias correction methods (Nemenman et al., 2002; Panzeri & Treves, 1996) were considered, but did not to provide a significantly better performance (data not shown). Population sparseness was computed using the definition in Vinje and Gallant (2000) (see main text). An initial 150ms transient was discarded from all simulations to allow the system to go from resting to steady state for the pattern being simulated. Independent recordings of the response to a pattern were extracted from a single simulation by slicing it in 30ms-long time frames and discarding every other frame, to allow the system state to decorrelate between the frames we kept for analysis. This is justified by the characteristic time of the autocorrelation function of the GC spiking output being of the order of 30ms (Figure S4B).

Table S3, related to Figure 7 and Supplemental Experimental Procedures: Parameter values used in the biologically constrained spiking model, and sources for the relevant experimental information.

Parameter	Value	Source	Parameter	Value	Source
E_m	-79.9 mV	Schwartz et al. (2012)	E_{NMDAR}	0 mV	Rothman et al. (2009)
G_m	1.06 nS		$a_1^{(NMDA)}$	17 nS	Schwartz et al. (2012)
C_m	3.22 pF		$a_2^{(NMDA)}$	2.645 nS	
V_t	-40 mV		$\rho^{(NMDA)}$	0.8647 ms	Rothman et al. (2009)
V_r	-63 mV		$\delta_1^{(NMDA)}$	13.52 ms	
τ_r	2 ms		$\delta_2^{(NMDA)}$	121.9 ms	
E_{GABAR}	-79.1 mV	Seja et al. (2012)	$r^{(NMDA)}$	0.0322	
G_{GABAR}	0.438 nS	Rothman et al. (2009)	$\Delta^{(NMDA)}$	236.1 ms	
E_{AMPAR}	0 mV		$\Pi^{(NMDA)}$	6.394 ms	
$a_1^{(AMPA,d)}$	3.724 nS		z	2	Schwartz et al. (2012)
$a_2^{(AMPA,d)}$	0.3033 nS		T	308.15K	
$\rho^{(AMPA,d)}$	0.3274 ms		$[Mg^{2+}]_{out}$	1 mM	
$\delta_1^{(AMPA,d)}$	0.3351 ms		δ_{bind}	0.35	
$\delta_2^{(AMPA,d)}$	1.651 ms		δ_{perm}	0.53	
$r^{(AMPA,d)}$	0.1249		C_1	2.07 mM	
$\Delta^{(AMPA,d)}$	131 ms		C_2	0.015 mM	
$a_1^{(AMPA,s)}$	0.2487 nS				
$a_2^{(AMPA,s)}$	0.2799 nS				
$a_3^{(AMPA,s)}$	0.1268 nS				
$\rho^{(AMPA,s)}$	0.5548 ms				
$\delta_1^{(AMPA,s)}$	0.4 ms				
$\delta_2^{(AMPA,s)}$	4.899 ms				
$\delta_3^{(AMPA,s)}$	43.1 ms				
$r^{(AMPA,s)}$	0.2792				
$\Delta^{(AMPA,s)}$	14.85 ms				

Note: because of how the STP mechanisms are defined, the maximum amplitude of a conductance pulse isolated in time will be of the order of $r^{(AMPA)} \cdot a^{(AMPA)}$ for AMPA and $b(V) \cdot r^{(NMDA)} \cdot a^{(NMDA)}$ for NMDA. This, in practice, means a maximum peak amplitude of 630pS for both AMPA (Sargent et al., 2005), and unblocked NMDA (Schwartz et al., 2012).

3 Appendix

3.1 Analytical method to compute granule cell empirical entropy in the Uniform Binary Network

The connectivity of the GC layer can be simplified to a random bipartite graph with two sets of nodes, one for the MF rosettes (inputs) and one for the GCs (outputs). Each output node is randomly connected to d inputs, corresponding to each GC having d synaptic connections. If an output node is connected with an input node, the graph is said to have an *edge* between the two. We modeled GCs as linear threshold (binary) units, active if the number of active binary synaptic inputs is greater than or equal to some value ϕ. In this appendix we refer to this spatially uniform binary network model as a UBN (in correspondence to the main text). Our aim in studying the UBN is to understand the properties of random feedforward network encoders.

Statement of mathematical problem: Encoding is performed by the UBN by mapping from N unique samples to M unique patterns on the input layer of the UBN (MFs), which are in turn mapped onto G unique patterns on the output layer (GCs). These mappings are deterministic, but the patterns arising on the input are randomly determined. Parameters of the network model determine both M and G. Since all mappings are deterministic, information is lost whenever the mapping from events to output patterns is not a bijection. We calculated the entropy of the G output patterns to quantify the conservation of the information of the N encoded samples. Hence we quantified how the network parameters impact the conservation of information. For notation, see Tables S4 and S5.

3.1.1 Mossy fiber inputs

Input patterns and symbols: MF synaptic rosettes are represented by the input layer of the UBN model. We denote the number of such input nodes as μ. Each input is either on or off and input patterns to the network are μ element binary vectors $\hat{\chi}$:$\{\chi_\lambda \in \{0,1\}\}$ for all $\lambda \in \{0,...,\mu-1\}$. Let X denote the set of all such vectors. Define $\Omega^{(X)}$ as the index set of N vectors chosen from X, containing the integers associated with each binary input vector in the following manner: for the lth vector $\hat{\chi}^{(l)} \to \omega_l^{(X)} = \sum_{\lambda=0}^{\mu-1} 2^\lambda \chi_\lambda^{(l)}$ where $l \in \{1,...,N\}$ and $\omega_l^{(X)} \in \{0,...,2^{\mu-1}\}$ is a single integer in the index set. Each one of the integers $\omega_l^{(X)}$ is referred to as a *symbol* in order to distinguish it from the *pattern* $\hat{\chi}^{(l)}$ associated with it (which is a vector). $\Omega^{(X)}$ is referred to as the input alphabet. Since there is a one-to-one mapping between patterns and symbols , they are identical from the point of view of Shannon information, Eq. (1). However, patterns can have properties, such as the number of active inputs while symbols should be viewed here as an accounting device to keep track of the number and probability of distinguishable patterns.

Input information and the alphabet: The pattern of activity from the sensory receptors of an animal is unlikely to ever repeat precisely due to the huge numbers of transducers involved in raw sensation, but repeated body movements in familiar environments could result in very similar MF activation patterns. Downstream networks may learn from MF states by clustering them (facilitated by expansion and sparsening in the GCL) thereby flexibly identifying occurrences of similar states. Maximum flexibility is afforded if the MF patterns retain as much of the raw sensory event information as possible prior to downstream learning. To reflect this picture of sensory encoding we consider random activation patterns occurring in MF afferents as a result of sensory motor states during short intervals of time, which we call events. In the biological context the precise input pattern that results from each event is determined by random factors that differ in detail from animal to animal: developmental, morphological and physiological factors such as the microscopic arrangement of sensory afferents carrying impulses from

each somatic area and the precise activity signature from sensory cells. Hence in our model each event (microscopic sensory state) is thought of as mapping to a pattern of activity (MF input vector) that is determined by a single random realization of the MF inputs, assuming that for this realization the binary input variables are independent and identically distributed Bernoulli trials $\hat{\chi} : p(\chi_\lambda = 1) = p_{MF}$ for all $\lambda \in \{0, .., \mu - 1\}$ (p_{MF} denoted $p(MF)$ in the main text). We make the simplifying assumption that the event $\rightarrow$ pattern transduction has no noise, such that the events map *deterministically* to the random MF patterns, i.e. although the MF patterns are themselves random, their mapping to sensory events is constant. We assume that the number of possible events is sufficiently large that in practice the events occurring within the lifetime of the organism are all unique. There are many ways in which N MF patterns can denote N events. We describe each one of these mappings from the events, $1, 2, ..., N$ to the integers in the input alphabet $\Omega^{(X)}$, using a mapping $m = \{1 \rightarrow \omega_1^{(X)}, 2 \rightarrow \omega_2^{(X)}, ..., N \rightarrow \omega_N^{(X)}\}$, where the ordering of events is not considered important. The set of all such mappings $\mathcal{M} = \{m_1, m_2, ..., m_Z\}$ corresponds to the set of all possible input alphabets for the sequence of N events. Each alphabet can be thought of as representing the way that the nervous system of an individual animal responds to events.

The empirical probability of occurrence of an input symbol given some alphabet $p(\omega^{(X)}|m)$: When sampling from a probability distribution, we call the *empirical probability* the fraction of the total outcomes accounted for by each unique outcome (i.e. the normalized frequency of outcomes). Applied to input symbols, the empirical probability only assumes knowledge of the input alphabet, requiring that we make minimal assumptions about the process generating the events themselves. The empirical input symbol probability is

$$p(\omega^{(X)}|m) = \frac{1}{N} \sum_{l=1}^{N} I(\omega_l^{(X)} = \omega^{(X)}, m) \tag{4}$$

where $I \in \{0, 1\}$ is an indicator function that adopts the value 1 if and only if code $\omega_l^{(X)}$ is equal to $\omega^{(X)}$ for $\omega_l^{(X)} \in m$. As the number of events tends to infinity ($N \rightarrow \infty$), the empirical probabilities tend to their true probabilities ($p(\omega^{(X)}|m) \rightarrow p(\hat{\chi})$),

whereupon the empirical symbol probability becomes independent of the alphabet.

Computation of the empirical probability of a symbol $p(\omega^{(X)}, s_X)$: The input success class c_{s_X} is the set of those symbols whose patterns have exactly s_X active inputs. Since the underlying probability measure of the MF patterns is that of μ independent identically distributed binary variables, the number of times a symbol is realized depends only on the success class s_X. Let $u_n^{s_X}$ denote the mean number of unique draws of a symbol from success class c_{s_X} after n samples from that class, where $l_{n-1}(\omega \in c_{s_X})$ is the probability that the nth code taken from success class s_X is unique (i.e. has not yet been sampled) after $n-1$ draws. Symbols are drawn randomly with equal probability, hence $l_{n-1}(\omega \in c_{s_X}) = \left[\binom{\mu}{s_X} - u_{n-1}^{s_X}\right] / \binom{\mu}{s_X}$,

$$
\begin{aligned}
u_n^{s_X} &= u_{n-1}^{s_X} + l_{n-1}(\omega \in c_{s_X}) \\
&= 1 + u_{n-1}^{s_X}\left(\frac{\binom{\mu}{s_X} - 1}{\binom{\mu}{s_X}}\right)
\end{aligned}
\tag{5}
$$

Letting $r_{s_X} = \left[\binom{\mu}{s_X} - 1\right] / \binom{\mu}{s_X}$ we can find a homogeneous recurrence relation that is solved using standard methods,

$$
\begin{aligned}
u_{n+1}^{s_X} &= 1 + u_n r_{s_X} \\
u_{n+1}^{s_X} - u_n^{s_X} &= 1 + u_n^{s_X} r_{s_X} - (1 + u_{n-1}^{s_X} r_{s_X}) \\
&= u_n^{s_X} r_{s_X} - u_{n-1}^{s_X} r_{s_X} \\
u_{n+1}^{s_X} &= u_n^{s_X}(1 + r_{s_X}) - u_{n-1}^{s_X} r_{s_X}
\end{aligned}
\tag{6}
$$

having characteristic equation,

$$
r_{s_X}^2 = (1 + r_{s_X}) r_{s_X} - r_{s_X} \tag{7}
$$

where we used the ansatz $u_n^{s_X} = r_{s_X}^n$ and divided through by $r_{s_X}^{n-1}$. Eq.(7) has

roots,

$$(1/2)\left(1 \pm (r_{s_X} - 1) + r_{s_X}\right) \tag{8}$$

the general solution to the recursion relation is obtained by summing the roots with indefinite weighting,

$$u_n^{s_X} = A + Br^n. \tag{9}$$

Specific solutions can now be obtained by determining coefficients in Eq.(9): solving for $u_0^{s_X} = 0$, we find $B = -A$ and when $u_1^{s_X} = 1$ (since the first draw must be unique), $A = -1/(r_{s_X} - 1)$, $B = 1/(r_{s_X} - 1)$,

$$u_n^{s_X} = \frac{(r_{s_X}^n - 1)}{(r_{s_X} - 1)}. \tag{10}$$

Hence the empirical probability of any single code in success class s_X within a finite sample of N events is $p(\omega^{(s_X)}) = p(s_X)/u_{Np(s_X)}^{s_X} = p(s_X)[(r_{s_X} - 1)/(r_{s_X}^{Np(s_X)} - 1)]$.

3.1.2 Granule cell output

Granule cells: GCs are represented by the output nodes of the network. We denote the number of such output nodes as γ. Each output node is either on or off ($\nu \in \{0, 1\}$) which is determined by the thresholded summed inputs,

$$\alpha = \sum_{i=1}^{\mu} I_i \chi_i \tag{11}$$

$$\nu = \begin{cases} 1 & \text{if } \alpha \geq \phi \\ 0 & \text{if } \alpha < \phi \end{cases} \tag{12}$$

where $I = 1$ if the granule cell is connected to MF input i, $I = 0$ otherwise and ϕ is the minimum number of inputs that must be active for the output to switch from off (0) to on (1). The vector $\hat{\nu}(\hat{\chi})$:$\{\nu_\kappa \in \{0, 1\}\}$ for all $\kappa \in \{0, .., \gamma - 1\}$ thus defines an output pattern for each input pattern $\hat{\chi}$ in X and we denote the set of all such output patterns Y.

Output patterns and symbols: We define the index set of the output patterns as $\Omega^{(Y)}$, containing symbols which are the integers $\omega_l^{(Y)} \in \{0, ..., 2^{\gamma-1}\}$ for all $l \in \{1, ..., N\}$ derived from output patterns using the identical method used to map the input patterns to integer symbols. The network therefore performs a mapping of a set of input symbols on to a set of output symbols $f : \omega_l^{(X)} \rightarrow \omega_l^{(Y)}$ for all l. This mapping determines the empirical output symbol probability (and hence how much information is captured) and the relative properties of the input and output patterns such as their activity levels. Our model contains no transmission noise so each output pattern occurs deterministically in response to an input pattern (which is itself deterministically mapped to an event by the event mapping). However, the random connections between inputs and outputs in combination with the variable output thresholding ϕ mean that in general not all input patterns are mapped to unique output patterns, permitting the empirical entropy of the output symbols to differ from the entropy of the input symbols in a manner that depends on network parameters. For a network to encode all of the event information, i) the activity level of the inputs must be within a range that allows their variation to fully encode the events and ii) the parameters of the network should ensure a one to one mapping from input symbols to output symbols.

Output information: For a given set of input symbols and a given network realization a measure of the information of the output symbols can be determined directly from the empirical entropy of the output symbols conditional on the network (Cover & Thomas, 1991),

$$H(\Omega^{(Y)}|\theta, m) = -\sum_{\omega^{(Y)} \in \Omega^{(Y)}} p(\omega^{(Y)}|\theta, m) \log_2[p(\omega^{(Y)}|\theta, m)] \tag{13}$$

where the empirical entropy of the output alphabet is conditional on the network realization θ and event input alphabet m. Even given an identical set of input symbols having identical probabilities of occurrence, Eq. (13) will evaluate differently for different networks. The conditional entropy itself is therefore a random variable. For this reason we seek the mean conditional entropy over network realizations and event mappings

$$\left\langle H(\Omega^{(Y)}|\theta,m)\right\rangle_{\Theta,\mathcal{M}} = \left\langle -\sum_{\omega^{(Y)}\in\Omega^{(Y)}} p(\omega^{(Y)}|\theta,m)\,\mathrm{log}_2[p(\omega^{(Y)}|\theta,m)]\right\rangle_{\Theta,\mathcal{M}}$$
$$= -\sum_{\omega^{(Y)}\in\Omega^{(Y)}} \left\langle p(\omega^{(Y)}|\theta,m)\,\mathrm{log}_2[p(\omega^{(Y)}|\theta,m)]\right\rangle_{\Theta,\mathcal{M}}$$

We calculate this average in the “annealed” approximation, i.e. substituting

$$\left\langle p(\omega^{(Y)}|\theta,m)\,\mathrm{log}_2[p(\omega^{(Y)}|\theta,m)]\right\rangle \simeq \left\langle p(\omega^{(Y)}|\theta,m)\right\rangle \mathrm{log}_2\left[\left\langle p(\omega^{(Y)}|\theta,m)]\right\rangle\right]$$

We have checked the validity of this approximation numerically and by examining the Taylor expansion of Eq. (13); deviations from the annealing assumption are small if $p(\omega^{(Y)}|\theta,m)\in[0,1]$, which is always true as p is a probability.

Approximation of the granule cell output entropy : We require the expectation of Eq. (13) with respect to the network realization and input symbols. Let Θ denote the index set of all possible networks existing between the outputs and the inputs. Each member θ of this set gives rise to a different mapping of input symbols onto output symbols. In analogy to the symbols, θ can be thought of as the integers associated with each binary vector indexing a given network realization. This vector consists of $\mu\gamma$ dimensions (it is an 'unrolled' connection matrix), with each value being equal to one if there is an edge between the corresponding input/output pair and zero otherwise. Given a single realization of the network and event mapping we can determine with certainty whether for that realization, some input symbol $\omega^{(X)}$ maps to some output symbol $\omega^{(Y)}$, since this mapping is deterministic. Hence we can define an indicator function on the sets $\Omega^{(Y)}$, $\Omega^{(X)}$, Θ and $\mathcal{M}$, which takes the value 1 if and only if output code $\omega^{(X)}$ occurs as a result of alphabet m, and gives rise to $\omega^{(Y)}$ for network

configuration θ. Let $I(\omega^{(Y)}, \omega^{(X)}, \theta, m)$ denote this function. Given a single network realization θ the probability of an output symbol must be the sum of the probabilities of the input symbols giving rise to it since the outputs are deterministic, $p(\omega^{(Y)}|\theta, m) = \sum_{\omega^{(X)} \in c_{\omega^{(Y)}}} p(\omega^{(X)}|m) = \sum_{\omega^{(X)}=0}^{2^{\mu}-1} p(\omega^{(X)}|m) I(\omega^{(Y)}, \omega^{(X)}, \theta, m)$, where $c_{\omega^{(Y)}}$ is the set of all input symbols that give rise to output symbol $\omega^{(Y)}$ for network θ and event mapping m. In Eq.(13) the logarithm is defined only for $\omega^{(Y)}$ which occur. Therefore we determine the mean empirical symbol probability in the case where $\omega^{(Y)}$ always exists in the output alphabet given the network realization θ and alphabet m. We develop this quantity further in due course, but for now we denote it $\{p(\omega^{(Y)}|\theta, m)\}_{\Theta,\mathcal{M}}$ where the curly braces indicate that this is an average over Θ and $\mathcal{M}$ under the probability measure in which $\omega^{(Y)}$ must exist. The expected conditional entropy is therefore,

$$
\begin{aligned}
H(\Omega^{(Y)}|\Theta, \mathcal{M}) \quad = \quad & - \sum_{\omega^{(Y)}=0}^{2^{\gamma}-1} \sum_{\omega^{(X)}=0}^{2^{\mu}-1} \left\langle p(\omega^{(X)}|m) I(\omega^{(Y)}, \omega^{(X)}, \theta, m) \right\rangle_{\Theta,\mathcal{M}} \cdot \\
& \cdot \log_2 [\left\{ \sum_{\omega^{(X)}=0}^{2^{\mu}-1} p(\omega^{(X)}|m) I(\omega^{(Y)}, \omega^{(X)}, \theta, m) \right\}_{\Theta,\mathcal{M}}]. \qquad (14)
\end{aligned}
$$

The remainder of the appendix is concerned with determining the components of Eq. (14) in terms of the macroscopic properties of the network only, such as the number of inputs, the number of outputs and the activity level of the inputs.

Determination of $\langle p(\omega^{(X)}|m) I(\omega^{(Y)}, \omega^{(X)}, \theta, \mathcal{M}_j) \rangle_{\Theta,\mathcal{M}}$ in Eq. (14): We first make the following observations: i) In the uniform binary model, the outputs are conditionally independent given the input $\omega^{(X)}$ since they are not themselves directly connected by any graph edges, hence

$$
\sum_{\theta=0}^{2^{\mu\gamma}-1} p(\theta) \prod_{j=1}^{\gamma} I(\nu_j = g_j, \omega^{(X)}, \theta, m) = p(\underline{\nu} = \underline{g}, \omega^{(X)}, m) = \prod_{j=1}^{\gamma} p(\nu_j = g_j | \omega^{(X)}, m) \qquad (15)
$$

ii) The input alphabet makes no difference to the probability of an output being active given some input symbol, $p(\nu_1 = g_1 | \omega^{(X)}, m) = p(\nu_1 = g_1 | \omega^{(X)})$ because only

the empirical probability of $\omega^{(X)}$ is affected by the choice of alphabet, not the state of a single output given that $\omega^{(X)}$ has occurred. Applying these observations,

$$\sum_{m\in\mathcal{M}} p(m)p(\omega^{(X)}|m) \sum_{\theta=0}^{2^{\mu\gamma}-1} p(\theta)I(\omega^{(Y)},\omega^{(X)},\theta,m) =$$

$$= \sum_{m\in\mathcal{M}} p(m)p(\omega^{(X)}|m) \sum_{\theta=0}^{2^{\mu\gamma}-1} p(\theta)\prod_{j=1}^{\gamma} I(\nu_j = g_j,\omega^{(X)},\theta,m) \quad \text{[applying (i) above]}$$

$$= \sum_{j=1}^{Z} p(m)p(\omega^{(X)}|m)\prod_{j=1}^{\gamma} p(\nu_j = g_j|\omega^{(X)},m) \quad \text{[applying (ii) above]}$$

$$= p(\omega^{(X)})p(\nu_1 = g_1|\omega^{(X)})...p(\nu_\gamma = g_\gamma|\omega^{(X)}) \quad (16)$$

where to perform the desired computation of Eq. (16) we must determine the conditional output activation probability $p(\nu = 1|\omega^{(X)})$ for a single output given presentation of an input symbol.

Determination of the conditional output activation probability $p(\nu = 1|\omega^{(X)})$ in Eq. (16): Every input symbol $\omega^{(X)}$ can be placed into a class c_{s_X} having some number s_X of active inputs. Consider the state of the UBN when a single input pattern from c_{s_X} is applied to the inputs. Each output is connected at random to the inputs and therefore samples both active and inactive inputs. Since every output makes d connections to the inputs, there can be no more than max(d, s_X) active inputs connected to each output and we wish to know how many active inputs are sampled. This can be seen as a sampling problem, where given a population of μ variables in which s_X are active, we wish to know how many active variables we sample after taking d samples without replacement; this is precisely the sampling described by the hypergeometric distribution. Therefore, the probability that each output is activated given s_X active inputs is the mass of the hypergeometric distribution between the threshold ϕ and the maximal active units max(d, s_X),

$$p(\nu = 1|\omega^{(s_X)}) = \sum_{z=\phi}^{\max(d,s_X)} \frac{\begin{pmatrix} s_X \\ z \end{pmatrix} \begin{pmatrix} \mu - s_X \\ d - z \end{pmatrix}}{\begin{pmatrix} \mu \\ d \end{pmatrix}} \tag{17}$$

where the notation $p(\nu = 1|\omega^{(s_X)})$ indicates the probability of activation of an output given presentation of a single input symbol from success class s_X.

Determination of $\left\{\sum_{\omega^{(X)}=0}^{2^\mu-1} p(\omega^{(X)}|m) I(\omega^{(Y)}, \omega^{(X)}, \theta, \mathcal{M}_j)\right\}_{\Theta,\mathcal{M}}$ in Eq. (14): Before derivation of this quantity we make some observations to guide us. Recall that this is defined as the expectation of the output symbol $\omega^{(Y)}$ probability given the input symbol $\omega^{(X)}$ running over only those input alphabet and connectivity combinations for which the output code $\omega^{(Y)}$ is realized. Imagine that we take a UBN with a fixed number of inputs and a fixed input alphabet and we attach a single output with random connections. We 'display' all input patterns on the MF inputs and evaluate the resulting empirical entropy of the output symbols after all N patterns are presented. We then randomly connect another output and repeat the procedure, before adding another and again repeating the procedure, thus growing the size of the output layer one unit at a time. As the number of network outputs increases, more entropy is recovered by the outputs from the inputs. Eventually, when all entropy is recovered the mapping f is one to one and the input symbol is fully determined by the output symbol. At this point, the empirical probability of some output symbol $p(\omega^{(Y)}|\theta, m)$ is either 0 (i.e. symbol $\omega^{(Y)}$ does not occur) or $p(\omega^{(X)}|m)$ where $\omega^{(X)}$ is the necessarily unique input symbol giving rise to $\omega^{(Y)}$. As we noted above, however, the average $\langle p(\omega^{(Y)})\rangle_{\Theta\mathcal{M}}$ inclusive of $\omega^{(Y)}$ that do not occur for a given θ and m tends to zero as $\gamma \to \infty$ for every symbol $\omega^{(Y)}$. This leads to an incorrect entropy since if we sum entropy over all possible $\omega^{(Y)} \in \Omega^{(Y)}$, $\langle H\rangle \to \infty$ which is incorrect since the entropy of the outputs cannot exceed the entropy of the inputs, which is bounded by the raw entropy of the events (where this bound is achieved if all $\omega^{(X)}$ are unique). For a small number of GCs every output symbol appears with finite probability (i.e.

all possible GC patterns are observable) and each of those symbols occurs in response to many input symbols. Hence, in this regime, output symbol probabilities are largely independent of the the connections and event mapping. However as the number of GCs increases, their output symbols appear only in response to a smaller number of input symbols. Hence output symbol occurrence becomes constrained by the specific connectivity of the network and choice of event mapping, until eventually single input symbols map uniquely to single output symbols in a manner that is completely determined by the specific network realization. As γ increases and this regime is approached, $\{p(\omega^{(Y)}|\theta, m)\} \sim p(\omega^{(X)}|m)$ becomes appropriate. Hence our definition of this quantity should exhibit this behavior in the limits of small and large γ. We first determine the conditional output symbol probability in the case that the output code exists,

$$\begin{aligned} p(\omega^{(Y)}|\theta, m) &= \sum_{\omega^{(X)}=0}^{2^{\mu}-1} p(\omega^{(X)}|m) I(\omega^{(Y)}, \omega^{(X)}, \theta, m) \\ &= \sum_{s_X=0}^{\mu} \sum_{\omega^{(X)} \in c_{\omega^{(Y)}}^{(s_X)}} p(\omega^{(X)}|m) \qquad \text{if } \omega^{(Y)} \text{exists; 0 otherwise} \end{aligned} \tag{18}$$

where $c_{\omega^{(Y)}}^{(s_X)}$ is the set of input symbol indices for patterns in class s_X that map to $\omega^{(Y)}$. The number of symbols in this set is $n_c = \left| c_{\omega^{(Y)}}^{(s_X)} \right|$. Therefore to determine the expected conditional probability of occurrence of output code $\omega^{(Y)}$ (assuming that $\omega^{(Y)}$ exists for network θ and input alphabet m) the expectation of the non-zero contribution to Eq. (18) is computed,

$$\left\{ \sum_{\omega^{(X)}=0}^{2^{\mu}-1} p(\omega^{(X)}|m) I(\omega^{(Y)}, \omega^{(X)}, \theta, m) \right\}_{\Theta,\mathcal{M}} = \sum_{\theta=0}^{2^{\mu\gamma}-1} p(\theta) \sum_{m\in\mathcal{M}} p(m) \sum_{s_X=0}^{\mu} \sum_{\omega^{(X)}\in c^{(s_X)}_{\omega^{(Y)}}} p(\omega^{(X)}|m)$$
$$= \sum_{\theta=0}^{2^{\mu\gamma}-1} p(\theta) \sum_{s_X=0}^{\mu} n_c p(\omega^{(s_X)})$$
$$= \sum_{s_X=0}^{\mu} p(s_X) \frac{\langle n_c \rangle_\Theta}{u^{s_X}_{Np(s_X)}}$$
$$= \sum_{s_X=0}^{\mu} p(s_X) a^{(s_X)}_{\gamma/s_Y} \quad (19)$$

where $a^{(s_X)}_{\gamma/s_Y}$ is the fraction of the input symbols in class s_X that lead to an output symbol $\omega^{(Y)}$ in class s_Y for a network having γ outputs ($u^{s_X}_{Np(s_X)}$ is defined in Eq. (10)). The average fraction of input symbols leading to some output symbol $\omega^{(Y)}$ over all network connectivities ($a^{(s_X)}_{\gamma/s_Y}$) depends on the number of active outputs (s_Y) in the output symbol (which is determined by each $\omega^{(Y)}$), and on the total number of outputs (γ), rather than on the specific connectivity θ or symbol $\omega^{(Y)}$. In the limit that $\gamma \to \infty$ only one input symbol can map to the output symbol ($a^{(s_X)}_{\gamma/s_Y} = 1/u^{s_X}_{Np(s_X)}$) and in this limit contributions to the output symbol probability from other input symbols must fall to zero. Therefore we may express this probability as a sum of the contribution due to the class of input symbols that $\omega^{(s_Y)}$ converges on as $\gamma \to \infty$, $a^{(s_X=s^*_X)}_{\gamma/s_Y}$ and contributions from the input classes whose contributions tend to zero this limit, $a^{(s_X\neq s^*_X)}_{\gamma/s_Y}$. Then,

$$\left\{ \sum_{\omega^{(X)}=0}^{2^{\mu}-1} p(\omega^{(X)}|m) I(\omega^{(Y)}, \omega^{(X)}, \theta, \mathcal{M}_j) \right\}_{\Theta,\mathcal{M}} = p(s^*_X) a^{(s_X=s^*_X)}_{\gamma/s_Y} + \sum_{\beta\neq s_X} p(\beta) a^{(s_X\neq s^*_X)}_{\gamma/s_Y} \quad (20)$$

To simplify the initial problem of determining $a^{(s_X=s^*_X)}_{\gamma/s_Y}$ and $a^{(s_X\neq s^*_X)}_{\gamma/s_Y}$ we consider the case where $\gamma = s_Y$ such that we consider the output symbol for which all outputs are activated ($\omega^{(Y)} = 2^{\gamma-1}$). Following our preceding arguments we compute $a^{(s_X)}_{\gamma/s_Y}$ as $s_Y = \gamma \to \infty$. For only one output, $a^{(s_X)}_{1/1} = p(\nu = 1|\omega^{(s_X)})$; and assuming only that every additional output unit reduces $a^{(s_X)}_{\gamma/s_Y}$ by some constant proportion we can

specify the recursion relation (dropping the $a^{(s_X)}_{\gamma/s_Y}$ notation using $a^{(s_X)}_{s_Y}$ here instead since $s_Y = \gamma$),

$$a^{(s_X)}_{s_Y} = a^{(s_X)}_{s_Y-1} - r(a^{(s_X)}_{s_Y-1} - b) \tag{21}$$

where r is the proportion of input symbols eliminated from $c^{(s_X)}_{\omega^{(Y)}}$ (for $\omega^{(Y)} \in$ success class s_Y) by each active output and b is some minimal fraction remaining in the limit $\gamma \to \infty$. Differencing and then solving the relation in the same manner as in the previous section,

$$\begin{aligned} a^{(s_X)}_{s_Y} &= a^{(s_X)}_{s_Y-1}(2-r) - a^{(s_X)}_{s_Y-2}(1-r) \\ &= A + B(1-r)^{s_Y} \end{aligned} \tag{22}$$

where, when $s_Y = \gamma = 0$, $a^{(s_X)}_0 = A + B$, while when $s_Y = \gamma \to \infty$, $a^{(s_X)}_{s_Y} \to A$. Therefore, to compute $a^{(s_X=s^*_X)}_{s_Y}$ let $A = p(\omega^{(X)}|s^*_X) = 1/u^{s^*_X}_{Np(s^*_X)}$ and hence $B = a^{(s_X=s^*_X)}_0 - 1/u^{s^*_X}_{Np(s^*_X)}$, while to compute $a^{(s_X \neq s^*_X)}_{s_Y}$ let $A = 0$ and hence $B = a^{(s_X)}_0$. Setting the fraction of input symbols removed from the set that causes output symbol $\omega^{(Y)} = 2^{\gamma-1}$ as those input symbols that do not permit activation of the $s_Y = (\gamma+1)$th output, $r = 1 - p(\nu_{s_Y} = 1|\omega^{(s_X)})$ (i.e. the complement of the probability that the output is active given the input) and setting the initial fraction of input symbols equal to the fraction that activate the first output $a^{(s_X)}_0 = p(\nu_1 = 1|\omega^{(s_X)})$,

$$a^{(s_X=s^*_X)}_{s_Y} = 1/u^{s_X}_{Np(s_X)} + [p(\nu = 1|\omega^{(s_X)}) - 1/u^{s_X}_{Np(s_X)}]p(\nu = 1|\omega^{(s_X)})^{s_Y} \tag{23}$$

$$a^{(s_X \neq s^*_X)}_{s_Y} = p(\nu = 1|\omega^{(s_X)})^{s_Y}. \tag{24}$$

Note that the solution for the case in which the output symbol converges on some

input symbol satisfies the requirements we set earlier, whereas the alternative solution is exactly the standard solution for the probability of the realization of $\{1, 1, ..., 1\}$ throughout a group of uncorrelated random binary variables. Eq. (23) applies to the case where all outputs are active and takes as its initial condition $a_0^{(s_X)}$, the proportion of symbols in class s_X that cause a single output to be activated ($p(\nu = 1|\omega^{(s_X)})$).

We now adapt the expression to apply to any output symbol success class where $s_Y \neq \gamma$. For a pattern having s_Y active outputs and $\gamma - s_Y$ inactive outputs we apply Eq. (23) with the initial condition $a_0^{(s_X)}$ taken as the fraction of input symbols in class s_X causing $\omega^{(Y)}$, assuming s_Y active outputs only (i.e. the initial condition for the recurrence for the $\gamma - s_Y$ inactive outputs is the end condition $a_{s_Y}^{(s_X)}$ of Eq. (23) in the case of s_Y active outputs). Hence

$$
\begin{aligned}
a_{\gamma/s_Y}^{(s_X = s_X^*)} &= 1/u_{Np(s_X)}^{s_X} + \left[[a_0^{(s_X)} - 1/u_{Np(s_X)}^{s_X}]p(\nu = 1|\omega^{(s_X)})^{s_Y}\right]\left(1 - p(\nu = 1|\omega^{(s_X)})\right)^{(\gamma - s_Y)} && (25)\\
a_{\gamma/s_Y}^{(s_X \neq s_X^*)} &= p(\nu = 1|\omega^{(s_X)})^{s_Y}\left(1 - p(\nu = 1|\omega^{(s_X)})\right)^{(\gamma - s_Y)}. && (26)
\end{aligned}
$$

Therefore, where subscript in refers to the inputs and subscript out refers to the outputs,

$$
\begin{aligned}
\left\{\sum_{\omega^{(X)}=0}^{2^\mu - 1} p(\omega^{(X)}) I(\omega^{(Y)}, \omega^{(X)}, \theta, \mathcal{M}_j)\right\}_{\Theta, \mathcal{M}} &= p(s_X^*) a_{s_Y}^{(s_X^*)} \\
&\quad + \sum_{\beta \neq s_X^*} p(\beta) p(\nu = 1|\omega_{in}^{(\beta)})^{s_Y}\left(1 - p(\nu = 1|\omega_{in}^{(\beta)})\right)^{(\gamma - s_Y)} \\
&= p(s_X^*) a_{s_Y}^{(s_X^*)} + \sum_{\beta \neq s_X^*} \binom{\mu}{\beta}\binom{\gamma}{s_Y} p(\omega_{out}^{(s_Y)}, \omega_{in}^{(\beta)}) / \binom{\gamma}{s_Y} \\
&= p(s_X^*) a_{s_Y}^{(s_X^*)} + \sum_{\beta \neq s_X^*} p(s_Y, \beta) / \binom{\gamma}{s_Y} && (27)
\end{aligned}
$$

The Entropy of the output symbols: For independent random binary inputs, $p(\omega^{(X)})$ depends only on the number of successes in each input pattern and the

number of events. Hence regrouping the input symbols in the sum of Eq. (16) across $\omega^{(X)}$ into input symbol success classes,

$$
\begin{aligned}
&\sum_{\omega^{(X)}=0}^{2^{\mu}-1} p(\omega^{(X)})p(\nu_1 = x_1|\omega^{(X)})...p(\nu_\gamma = x_\gamma|\omega^{(X)}) = \\
&\quad = \sum_{s_x=0}^{\mu}\left(\sum_{\omega^{(X)}\in c^{(s_X)}_{\omega^{(Y)}}} p(\omega^{(s_X)})p(\nu_1 = x_1|\omega^{(s_X)})...p(\nu_\gamma = x_\gamma|\omega^{(s_X)})\right) \\
&\quad = \sum_{s_x=0}^{\mu}\binom{\mu}{s_X}p(\omega^{(s_X)})p(\nu_1 = x_1|\omega^{(s_X)})...p(\nu_\gamma = x_\gamma|\omega^{(s_X)}) \\
&\quad = \sum_{s_x=0}^{\mu}\binom{\mu}{s_X}p(\omega^{(s_X)})p(\nu = 1|\omega^{(s_X)})^{s_Y}[1 - p(\nu = 1|\omega^{(s_X)})]^{(\gamma - s_Y)} \qquad (28)
\end{aligned}
$$

Combining this and other previous results,

$$
\begin{aligned}
H(\Omega^{(Y)}|\Theta,\mathcal{M}) &= -\sum_{\omega^{(Y)}=0}^{2^{\gamma}-1}\sum_{\omega^{(X)}=0}^{2^{\mu}-1}\left\langle p(\omega^{(X)})I(\omega^{(Y)},\omega^{(X)},\theta,m)\right\rangle_{\Theta,\mathcal{M}} \cdot \\
&\quad \cdot \log_2[\left\{\sum_{\omega^{(X)}=0}^{2^{\mu}-1} p(\omega^{(X)})I(\omega^{(Y)},\omega^{(X)},\theta,m)\right\}_{\Theta,\mathcal{M}}] \\
&= -\sum_{s_Y=0}^{\gamma}\binom{\gamma}{s_Y}\sum_{s_x=0}^{\mu}\binom{\mu}{s_X}p(\omega^{(s_X)})p(\nu = 1|\omega^{(s_X)})^{s_Y}[1 - p(\nu = 1|\omega^{(s_X)})]^{(\gamma - s_Y)} \cdot \\
&\quad \cdot \log_2[p(s_X)a_{s_Y}^{(s^*_X)} + \sum_{s_X\neq\beta} p(\beta, s_Y)/\binom{\gamma}{s_Y}] \\
&= -\sum_{s_Y=0}^{\gamma}\sum_{s_x=0}^{\mu} p(s_X, s_Y)\log_2[p(s_X)a_{s_Y}^{(s^*_X)} + \sum_{\beta\neq s_X} p(\beta, s_Y)/\binom{\gamma}{s_Y}]. \qquad (29)
\end{aligned}
$$

The direct method for computing the outputs of a UBN given an input (as outlined in section 2.6.1, above) takes a time (τ_M) proportional to the product of the dimensions of the connectivity matrix, $\mu\gamma$, and the number of events; hence $\tau_M \sim N\mu\gamma$. When implemented in optimized form the time (τ_A) required to compute the entropy with Eq. (29) scales as $\tau_A \sim (\mu+\gamma)^3$. For small numbers of patterns, $\tau_M < \tau_A$ and the direct method is the fastest way to determine the UBN

entropy. However, for a network of the size used in this study, taking $\tau_M = \tau_A$ and solving for N indicates that Eq. (29) outperforms the naive method when the number of events exceeds around 4000. In practice this value is much lower because τ_M does not account for the overhead in generating the random inputs or creating the empirical distribution and computing the Shannon information over that distribution. Since we computed our entropy estimate for 1×10^9 events, Eq. (29) provided a significant advantage. To gather our full dataset, however, still required significant computational resources due to our explicit search over a large number of points in parameter space; so we deployed our method using custom parallelized C code running on a compute cluster. Due to the large values of the arguments of the binomial coefficients in our calculation, standard floating point arithmetic would experience overflow errors. To prevent this we utilized multi-precision arithmetic. We avoided using an approximation such as Stirling's formula due to concern about accumulation of numerical errors when one or both arguments of the binomial coefficient are small.

3.2 Mathematical definitions used in the derivation of the entropy

Table S4, related to the Appendix to the Supplemental Experimental Procedures: Mathematical notation for the input layer

Symbol:	Meaning	Illustrative value
μ	Number of MF input nodes	176
$\hat{\chi}$	An **input pattern** (vector of binary MF values)	$0, 0, 1, 0, ..., 1$
X	The set of all possible input patterns	$\{\{0, 0, ..., 0\}, \{1, 0, ..., 0\}, ...\}$
N	Number of events encoded	1×10^9
$\omega^{(X)}$	**Input symbol:** Integer representation of an input pattern	12
$\Omega^{(X)}$	**Input alphabet:** Integer index set of some subset of input patterns	$\{12, 14, ..., 2\}$
p_{MF}	Probability that MF is active when a pattern is generated	0.1
$p(\hat{\chi})$	Probability of an input pattern sampled in response to an event	$0.1 \times 0.1 \times ... \times 0.9$
m	**Event mapping:** mapping from events to input symbols	$\{1 \to 12, ..., N \to 2\}$
$\mathcal{M}$	The set of all event mappings	$\{m_1, m_2, ..., m_Z\}$
$p(\omega^{(X)})$	Probability of occurrence of input symbol $\omega^{(X)}$	0.0001
$p(\omega^{(X)}\|m)$	Empirical input symbol probability given event mapping m	0.0001
I	Indicator function	$[0, 1]$
s_X	**Input success class:** Number active MFs in an input pattern	10
$u^{s_X}_{Np(s_X)}$	Mean number of unique codes in MF success class s_X after N events	174.2

Table S5, related to the Appendix to the Supplemental Experimental Procedures: Mathematical notation for the output layer

Symbol:	Meaning	Illustrative value
γ	Number of GC output nodes	509
$\hat{\nu}$	An **output pattern** (vector of binary GC values)	$0, 0, 1, 0, ..., 1$
d	**Number of GC synaptic connections:** Number MFs connected	4
ϕ	**GC threshold:** Number of active MF inputs for GC to activate	2
Y	Set of all possible output patterns	$\{\{0, 0, ..., 0\}, \{1, 0, ..., 0\}, ...\}$
$\omega^{(Y)}$	**Output symbol:** Integer representation of an output pattern	37
$\Omega^{(Y)}$	**Output alphabet:** Integer index set of some subset of output patterns	$37, 14, ..., 76$
$p(\nu = 1 \vert \omega^{(X)})$	GC activation probability given input symbol $\omega^{(X)}$	0.1
f	Mapping from input symbols to output symbols	$\Omega^{(X)} \to \Omega^{(Y)}$
θ	Integer indexing a single network realization	Large integers
$p(\omega^{(Y)} \vert \theta, m)$	Conditional empirical probability of $\omega^{(Y)}$ given network θ and m	0.0001
$H(\Omega^{(Y)} \vert \theta, m)$	Conditional entropy of $\Omega^{(Y)}$ given network θ and and m	29.9 bits
Θ	The set of all network realizations	$\{0, 1, ..., 2^{\mu\gamma-1}\}$
$H(\Omega^{(Y)} \vert \Theta, \mathcal{M})$	Expected conditional entropy of $\Omega^{(Y)}$ given networks Θ and event mappings $\mathcal{M}$	29.9 bits
$c_{\omega^{(Y)}}$	The set of input symbols that cause output symbol $\omega^{(Y)}$	$24, 10, ..., 17$
$c^{(s_X)}_{\omega^{(Y)}}$	The set of input symbols in class s_X that cause output symbol $\omega^{(Y)}$	$1, 4, ..., 64$
s_Y	**Output success class:** The number of active GCs in an output pattern	10
$a^{(s_X)}_{\gamma/s_Y}$	Mean fraction of input codes in s_X leading to output codes in s_Y for γ GCs	0.001
$a^{(s^*_X)}_{\gamma/s_Y}$	As $a^{(s_X)}_{\gamma/s_Y}$ when output symbol occurs for input symbol in s_X when f is $1:1$	0.001
$a^{(s_X \emptyset)}_{\gamma/s_Y}$	As $a^{(s^*_X)}_{\gamma/s_Y}$ but output symbol does not occur for s_X when f is $1:1$	0
$p(s_X, s_Y)$	The joint distribution of input and output pattern success classes	Matrix

Supplemental References

Arthur, D. & Vassilvitskii, S. (2007). K-means++: The advantages of careful seeding. In Proceedings of the Eighteenth Annual ACM-SIAM Symposium on Discrete Algorithms, SODA '07, pp. 1027–1035. (Philadelphia, PA, USA: Society for Industrial and Applied Mathematics).

Brandes, U., Eiglsperger, M., Herman, I., Himsolt, M., & Marshall, M. (2002). Graphml progress report structural layer proposal. In Graph Drawing, P. Mutzel, M. Jünger, & S. Leipert, eds., vol. 2265 of *LNCS*. (Springer Berlin Heidelberg), pp. 501–512.

Collette, A. (2013). Python and HDF5. (O'Reilly Media).

Cover, T. M. & Thomas, J. A. (1991). Elements of information theory. (New York: Wiley).

Deb, K. & Padhye, N. (2010). Development of efficient particle swarm optimizers by using concepts from evolutionary algorithms. In Proceedings of the 12th annual conference on Genetic and evolutionary computation, pp. 55–62. ACM.

Garrett, A. L. (2014). inspyred. http://inspyred.github.com/.

Gundersen, H., Bagger, P., Bendtsen, T., Evans, S., Korbo, L., Marcussen, N., Moller, A., Neilsen, K., Nyengaard, J., & Pakkenberg, B. (1988). The new stereological tools: disector, fractionator, nucleator and point sampled intercepts and their use in pathological research and diagnosis. Apmis, 96, 857–881.

Hagberg, A. A., Schult, D. A., & Swart, P. J. (2008). Exploring network structure, dynamics, and function using NetworkX. In Proceedings of the 7th Python in Science Conference (SciPy2008), pp. 11–15. Pasadena, CA USA.

Ince, R. A. A., Petersen, R. S., Swan, D. C., & Panzeri, S. (2009). Python for information theoretic analysis of neural data. Front. Neuroinform., 3, 4.

Lloyd, S. (1982). Least squares quantization in pcm. IEEE Trans. Inf. Theory, 28, 129–137.

Nemenman, I., Shafee, F., & Bialek, W. (2002). Entropy and inference, revisited. In NIPS 14. (MIT Press).

Panzeri, S., Senatore, R., Montemurro, M. A., & Petersen, R. S. (2007). Correcting for the sampling bias problem in spike train information measures. J Neurophysiol, 98, 1064–1072.

Panzeri, S. & Treves, A. (1996). Analytical estimates of limited sampling biases in different information measures. Network - Comp. Neural., 7, 87–107.

Pedregosa, F., Varoquaux, G., Gramfort, A., Michel, V., Thirion, B., Grisel, O., Blondel, M., Prettenhofer, P., Weiss, R., Dubourg, V., Vanderplas, J., Passos, A., Cournapeau, D., Brucher, M., Perrot, M., & Duchesnay, E. (2011). Scikit-learn: Machine learning in Python. JMLR, 12, 2825–2830.

Rothman, J. S. & Silver, R. A. (2014). Data-driven modeling of synaptic transmission and integration. In Computational Neuroscience, K. T. Blackwell, ed., vol. 123 of *Progress in Molecular Biology and Translational Science*. (Academic Press), pp. 305 – 350.

Sargent, P. B., Saviane, C., Neilsen, T. A., DiGregorio, D. A., & Silver, R. A. (2005). Rapid vesicular release, quantal variability, and spillover contribute to the precision and reliability of transmission at a glomerular synapse. J. Neurosci., 25, 8173–8187.

Sawtell, N. B. (2010). Multimodal integration in granule cells as a basis for associative plasticity and sensory predication in a cerebellum-like circuit. Neuron, 66, 573–584.

Strong, S. P., Koberle, R., de Ruyter van Steveninck, R. R., & Bialek, W. (1998). Entropy and information in neural spike trains. Phys. Rev. Lett., 80, 197–200.

Tsodyks, M. & Markram, H. (1997). The neural code between neocortical pyra-

midal neurons depends on neurotransmitter release probability. PNAS, 94, 719–723.
